# Genome-Wide, Non-Invasive Prenatal Testing for rare chromosomal abnormalities: A systematic review and meta-analysis of diagnostic test accuracy

**DOI:** 10.1371/journal.pone.0308008

**Published:** 2024-11-05

**Authors:** Marton Konya, Agnes Czimbalmos, Lotti Loczi, Tamas Koi, Caner Turan, Rita Nagy, Nandor Acs, Peter Hegyi, Szabolcs Varbiro, Aniko Gal

**Affiliations:** 1 Centre for Translational Medicine, Semmelweis University, Budapest, Hungary; 2 Czeizel Institute, Budapest, Hungary; 3 Department of Obstetrics and Gynecology, Semmelweis University, Budapest, Hungary; 4 Department of Anesthesiology and Intensive Therapy, Semmelweis University, Budapest, Hungary; 5 Heim Pál National Pediatric Institute, Budapest, Hungary; 6 Institute of Pancreatic Diseases, Semmelweis University, Budapest, Hungary; 7 Department of Obstetrics and Gynecology, University of Szeged, Szeged, Hungary; 8 Workgroup for Science Management, Doctoral School, Semmelweis University, Budapest, Hungary; 9 Institute of Genomic Medicine and Rare Disorders, Semmelweis University, Budapest, Hungary; Wachemo University, INDIA

## Abstract

Genome-Wide Non-Invasive Prenatal Testing (GW-NIPT) can provide positive results not only for common autosomal aneuploidies but also for rare autosomal trisomies (RATs) and structural chromosomal abnormalities (StrCAs). Due to their rarity, there is currently insufficient information on positive predictive value PPV of RAT and StrCA-positive cases in the literature. In this study, the screening accuracy and pregnancy outcomes of cases positive for rare chromosomal abnormalities were examined based on publications in which GW-NIPT testing was performed. True positive cases were determined using two different methodologies. One was a confirmed methodology, where only cases validated by genetic testing were considered true positives with a definite diagnosis, and the other was an extended methodology, where, in addition to cases confirmed by genetic testing, intrauterine fetal death and termination of pregnancy due to an abnormality confirmed by ultrasound examination were also considered true positives, where no diagnosis had been made but the fetus was probably affected. Seventeen studies were analyzed, with a total GW-NIPT population of 740,076. Of these, 1,738 were RAT positive. Using the confirmed method, we found the highest rates of true positives in T16, followed by T22, and T2, using the extended method, the highest rate of true positives in T15, T16 and T22. This is the first meta-analysis to determine the frequency of rare chromosomal abnormalities, test-positive rates, and the PPV of each chromosomal abnormality with high precision. Our results could aid pre- and post-test genetic counselling and help patients and clinicians in their decision-making.

## 1. Introduction

Over the lIn Gast decade, molecular biology technologies and, with them, genetic diagnostic methods have undergone a significant transformation. This rapid development is therefore also present in prenatal screening, where combined screening based on biochemical methods and later PCR-based analysis of the most common chromosomal abnormalities will be replaced by targeted or genome-wide screening using next-generation sequencing from cell-free DNA (cfDNA). In general, genome-wide sequencing appears to be efficient and cost-effective alternative to current genetic testing. With the improvement of analytical methods over the last decade, its clinical utility will continue to grow and may in the future be incorporated into daily clinical practice in an increasing number of medical disciplines [1]. In recent years, genome sequencing for prenatal diagnostics has become available, offering a comprehensive and genome-wide approach to screening for fetal genetic disorders. The implementation of diagnostic exome and genome sequencing screening tests on invasively and non-invasively collected fetal DNA samples has transformed prenatal genetic diagnosis [2]. NIPT is a screening test that analyzes cfDNA in the blood of pregnant women to detect chromosomal abnormalities in the developing fetus.

Small cfDNA molecules are released into the circulation by apoptosis, necrosis, or secretion [3]. They can be obtained from the fetus, tumors, and circulating mitochondrial and viral DNA molecules [4]. This method was first applied in 1997 to detect the Y-chromosome and thus the sex of the fetus [5]. The first study on cfDNA in fetal screening for Down syndrome was published in 2008 [6]. Cell-free fetal DNA (cffDNA) is a subset of cell-free DNA (cfDNA) derived from placental trophoblasts and circulates freely in maternal blood. It is found in maternal blood as early as five to seven weeks of gestation and increases as the pregnancy progresses, with approximately 10 to 20% of the cell-free DNA in maternal blood being of fetal origin. The difference in size between cffDNA fragments (approximately 200 base pairs) and maternal DNA fragments allows cffDNA to be distinguished from maternal DNA [7]. The use of genome-wide screening of prenatal testing has several advantages, including early detection because it can be performed as early as 10 weeks of pregnancy and provides results without invasive procedures. NIPT can provide early and accurate detection of a wide range of chromosomal abnormalities with high sensitivity and specificity, while minimizing the need for invasive procedures and reducing maternal anxiety with false positive results [8].

Nowadays, cfDNA from plasma samples of pregnant women is widely used to investigate fetal chromosomal aneuploidies, including Down syndrome (trisomy 21, T21), Edwards syndrome (trisomy 18, T18), Patau’s syndrome (trisomy 13, T13) and sex chromosomal aneuploidies (SCA). For common chromosomal abnormalities (T21, T13, T18, and SCAs), NIPT has higher sensitivity, lower false positive rate, and higher PPV compared to conventional serum screening [9]. In recognition of these preliminary results, both the American College of Obstetricians and Gynecologists and the American College of Medical Genetics and Genomics have supported the routine implementation of NIPT screening in prenatal diagnostics [10]. The potential of NIPT, particularly in countries with higher GDP, has rapidly transformed global prenatal screening, and the test is increasingly being performed as part of the first-trimester screening of pregnancies worldwide. In 2015, laboratories started to use GW-NIPT technology, which can screen not only common aneuploidies but also rare autosomal aneuploidies and StrCAs. In the field of genetic diagnostics, knowledge of the sensitivity and specificity of the tests and methods used is crucial, the most appropriate parameter being positive predictive value (PPV) [11]. In the case of rare abnormalities, the PPV is the best predictor of the risk of test-positive cases. This value indicates the probability that a screening test will be true positive for a given disorder.

The aim of the present study was to evaluate the specificity and accuracy of GW-NIPT for rare autosomal trisomies (RATs) on a chromosome-by-chromosome basis StrCAs such as duplications and deletions on all chromosomes. to determine its clinical utility. The findings of our meta-analysis may help clinicians in pre- and post-test genetic counselling during prenatal diagnostics and support further decision-making in rare chromosomal abnormalities.

## 2. Methods

We performed a meta-analysis in accordance with the Cochrane Handbook for Systematic Reviews of Interventions [8]. This protocol was registered in PROSPERO, the International Database of Prospectively Registered Systematic Reviews, under the identification number CRD42022377120.

### 2.1 Eligibility criteria

All analytical studies with original research data were included in this systematic review and meta-analysis.

To determine the appropriateness of the primary research question, the population, intervention, and diagnostic test (PID) framework followed, where the target population was pregnant women who had undergone GW-NIPT screening, an index test where patients with GW-NIPT positive results were referred to for RATs and/or StrCAs. The diagnosis was confirmed by genetic testing of amniotic fluid, chorionic villus sampling, aborted fetuses, or postnatal peripheral blood. The diagnosis was established by karyotyping, chromosomal microarray analysis, ultrasound diagnosis, or pregnancy outcome. In all selected papers, the NIPT methodology used massive parallel shotgun sequencing.

The primary outcome was to test the PPV of GW-NIPT for all rare chromosomal aneuploidies and pooled for StrCAs. Secondary outcomes included the relative frequency of GW-NIPT-positive cases and true positive cases for each rare aneuploidy.

The following studies were excluded: case-control studies, case reports, case serial reports, cross-sectional studies, reviews, animal studies, cost-effectiveness studies, and studies with only data for frequent chromosome abnormalities. Studies that did not confirm RATs by invasive prenatal testing and/or postnatal karyotyping were also excluded.

### 2.2 Selection process

Two independent review authors (M.K. and A.C.) selected the articles using the EndNote X9 (Clarivate Analytics, Philadelphia, PA, USA) reference management program. Publications were first screened by title and abstract, then by full text according to eligibility criteria. A third independent review author (A.G.) resolved disagreements during the selection process.

### 2.3 Data collection process

The following data were extracted: title, first author, year of publication, study design, population screened, inclusion and exclusion criteria, and details of PID.

To estimate the accuracy of the test, we recorded the number of women screened with GW-NIPT true-positive and false-positive results overall for RATs and StrCAs.

### 2.4 Information sources and search strategy

The systematic search was carried out in 2 stages. In the first, using MEDLINE (via PubMed) and Embase databases from November 2022 to August 2023, and in the second, updated in stages up to February 2024, supplemented by the Web of Science database. The systematic search was carried out with the following predefined search key: (nipt OR nips OR nipd OR non invasive prenatal OR cell-free) AND (rare OR aneuploidy OR trisomy OR autosomal).

No filters or language restrictions were applied during the search. Seventeen studies were available in English and one in Chinese.

For studies from the same country, we checked for overlaps and used the most recent version where found. For two studies from Australia, we were not sure that there was no overlap, so we contacted the corresponding author by email who confirmed that there was no overlap in the two databases.

### 2.5 Study risk of bias assessment

The risk of bias was assessed (M.K. and A.C) based on the recommendations of the Cochrane Collaboration, using the Cochrane risk-of-bias tool for systematic reviews of diagnostic accuracy studies (QUADAS-2). Disagreements between data extractors were resolved by involving a third reviewer (A.G).

### 2.6 Statistical method

Statistical analyses were performed using the package `meta`of the R statistical software (version 4.1.2.). The statistical analyses followed the advice of [12] For all statistical analyses, a p-value less than 0.05 was considered significant. All the applied analyses were random-effects meta-analyses.

To pool the proportions (including the PPV), we used the generalized mixed-effects approach [13]. For small or zero proportions, this approach is definitely a better choice for meta-analysis than the classical inverse variance approach.

We calculated means and standard deviations in the true positive (TP) and false positive (FP) groups and performed a meta-analysis using the restricted maximum likelihood approach for mean differences with the Hartung-Knapp adjustment. In the calculations, the mean of the FP group was subtracted from the mean of the TP group.

For prevalence, the effect size and the standard error are dependent. For this reason, following the suggestion of [14], we created a modified funnel plot to visually assess publication bias: on the y-axis, we plotted the study size instead of the standard error. In addition, instead of Egger’s test, we used Peters’ test [15] to test whether publication bias was present or not. Due to data sparsity, we only performed publication bias analysis in all trisomy-positive cases.

In addition to the prediction interval, heterogeneity was assessed by calculating the univariate I^2^ measure. I^2^ values of 25%, 50%, and 75% were considered as low, moderate, and high heterogeneity, respectively.

### 2.7 Synthesis methods

#### 2.7.1 Assessing the level of evidence

The quality of evidence was assessed following the recommendations of the “Grades of Recommendation, Assessment, Development, and Evaluation (GRADE)” workgroup [16].

### 2.8 Analysis methods

In this meta-analysis, GW-NIPT results were examined on all chromosomes. We excluded the most common trisomies (T13, T18, and T21) and SCAs from the analysis, and focused only on RATs and StrCAs.

In this study, true positive cases were determined using two different methodologies. One was a confirmed methodology, where only cases confirmed by genetic testing were considered true positives, and the other one was an extended methodology, where, in addition to cases confirmed by genetic testing, intrauterine fetal death and termination of pregnancy due to an abnormality confirmed by ultrasonography were also considered true positives, as these cases were also likely to involve fetuses.

## 3. Results

### 3.1 Search and selection

**Fig 1:**
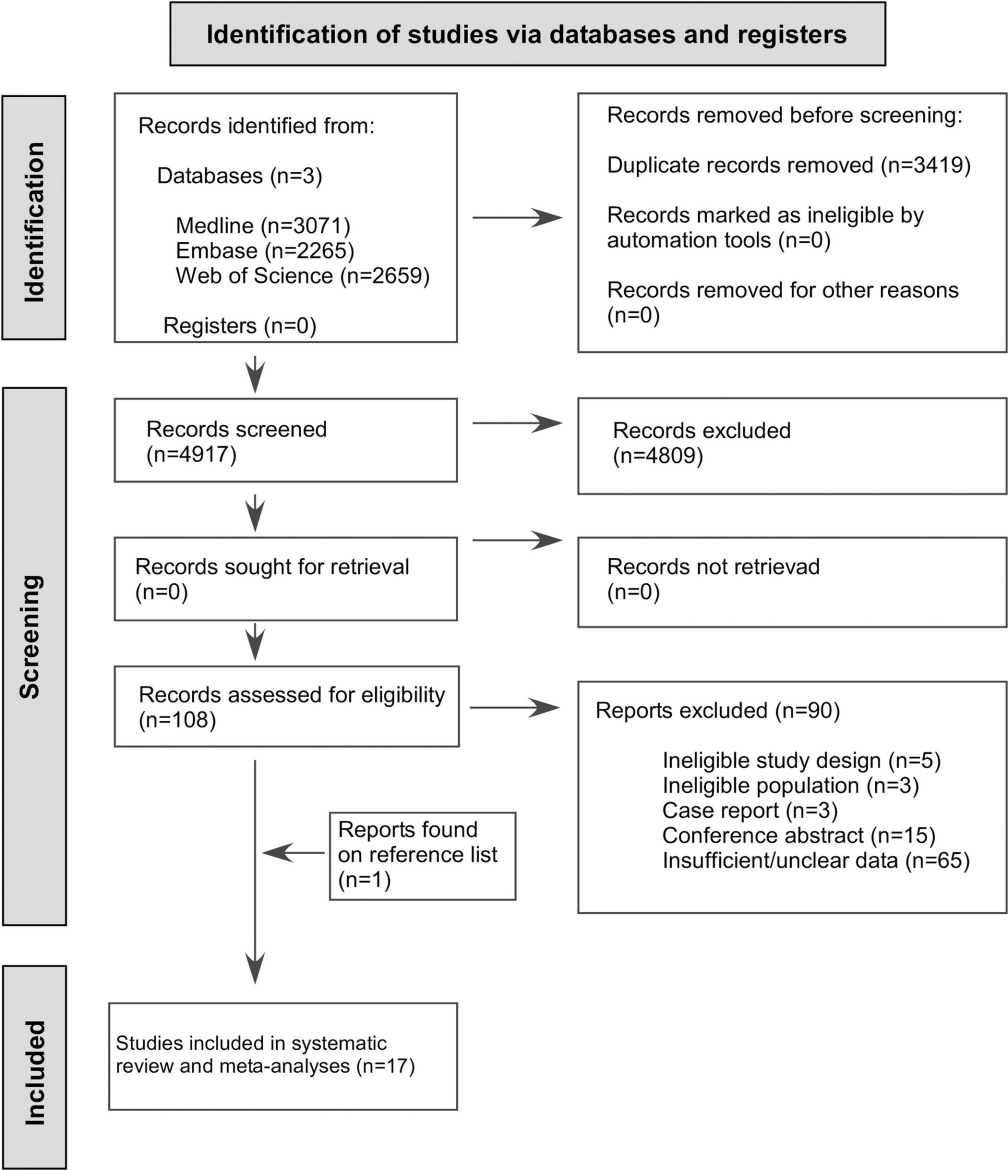
PRISMA 2020 flowchart representing the study selection process.

Of the 8,336 records, 17 studies were included in the meta-analysis with 740,076 GW-NIPT test analyses (Fig 1) [17–33].

### 3.2 Basic characteristics of studies included

The basic characteristics of studies included are detailed in Table 1. All studies used a massively parallel sequencing technology. All 17 studies reported rare trisomies, 10 of them StrCAs as well. Mean maternal age was available from 10 studies and was 33.55 years (SD: 2.06), and gestational age was 13.5 SD (2.73) weeks, also from 10 studies (Table 1).

**Table 1:** Basic characteristics of the included studies.

Study (year) / Study period	Country	Target population	NIPT primary screening or secondary screening	Article type	Structural variation tested	Number of investigated patients	Age mean	GA mean
Lingshan, 2020/ March 2017 to February 2020	China	high-risk pregnancies, pregnancies whose conception used ART, history of adverse reproductive outcome	primary screening	RS	no	18,016	30,0	18,0
Mossfield, 2022/ May 2021 to October 2021	Australia, Canada, Argentina, South Africa	general or high-risk	NA	RS	no	NA	36,1	11,1
Scott, 2018/ March 2015 to August 2018	Australia	singleton pregnancy, with no obvious abnormality, at a minimum of 10 weeks’ gestation at sample collection	NA	PS	no	23,388	35,5	11,4
Harasim, 2022/ October 2019 to September 2021	Germany	mixed risk profile	NA	RS	yes	3,664	34,3	13,0
Schuurman, 2022/ April 2017—April 2019	Netherland	high-risk pregnancies were not included	primary screening	RS	yes	149,318	32,9	12,4
Bogaert, 2021/ 2018 January—2019 June	Belgium	higher-order pregnancies were not included	primary screening	RS	no	153,575	30,7	NA
Wan, 2018/ February 2015 to 2018 January	China	the pregnancy had to be above 12 gestational weeks	secondary screening	RS	no	15,362	33,0	15,0
Pertile,2017/ NA	Australia	systematically analyzed WGS data from all chromosomes in two independent clinical laboratories	NA	RS	yes	89,817	34,6	13,8
Pescia, 2018/ NA	Switzerland	two consecutive data sets based on test reports by board-certified laboratory geneticists were retrieved from the clinical database	NA	RS	yes	6,388	NA	NA
Opstal, 2018/ April 2014 and April 2015	Netherland	high-risk pregnancies	secondary screening	RS	yes	2,527	NA	NA
Lin, 2021/ January 2014 and December 2020	China	pregnancy with a high-risk RATs report and complete clinical information	NA	RS	no	65,752	31,6	17,7
Brady, 2016/ NA	Belgium	high risk and low risk groups	NA	PS	yes	4	NA	NA
Fiorentino, 2017/ December 2015 and May 2016	Italy	pregnant women undergoing conventional cfDNA-based NIPT for common fetal aneuploidy	secondary screening	PS	yes	12,078	35,3	12,3
Basaran, 2022/ November 2013 and November 2022	Turkey	consecutive cases	NA	RS	yes	NA	34,6	17,1
Xiang, 2023/ March 2021 and March 2022	China	NIPT is a routine screening test for pregnant women at these hospitals and centers after 12 weeks of gestation	primary screening	RS	no	89,242	31,0	17,0
Zhang, 2023/ May 2018 and March 2022	China	singleton pregnancies, critical risk value of serological screening for pregnant women and high-risk value of maternal serological screening, single fetal soft markers identified by ultrasound	secondary screening	RS	yes	81,518	NA	NA
Xiaoxiao, 2023/ January 2019 and April 2023	China	high-risk cases	NA	RS	no	25,282	31,8	NA

Inclusion and exclusion criteria for the studies included are summarized in S1 Table in S1 File. A summary table of the study population and the technical details of the tests applied can be found in S2 Table in S1 File.

### 3.3 Proportion and PPV of GW-NIPT positive results for RATs

**Fig 2:**
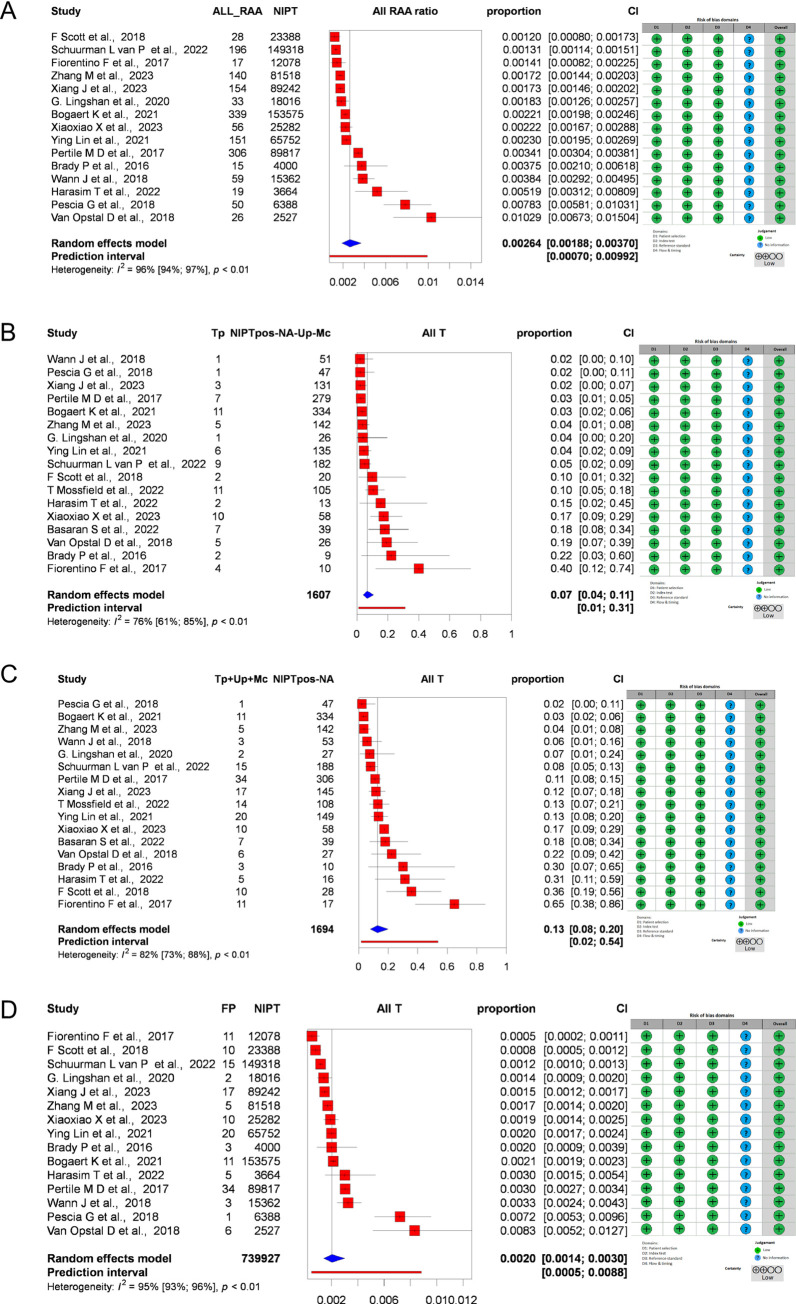
Forest plots representing the diagnostic test accuracy of rare trisomies. A: Forest plot representing the pooled frequency of GW-NIPT positive results for all rare autosomal trisomies (RATs), B: Forest plot representing the PPV with a confirmed method for all RATs, C: Forest plot representing the PPV with an extended method for all RATs, D: Forest plot representing the pooled false positive RAT rate, (Abbreviations: NIPT: non-invasive prenatal test, ALL_RAA: all rare autosomal aneuploidies, Tp: true positive cases, Up: Ultrasound positive cases, Mc: Miscarriage, NA: not available).

For an proportion analysis, 15 studies were analyzed, and 2 studies that included only positive NIPT cases were excluded. In these 15 studies, the total GW-NIPT population was n = 739,927, and 1,589 were RAT positive. The pooled proportion was 0.0026 (95% CI: [0.00196, 0.0037] PI: 0.0007; 0.0099 I^2^ = 96 [94%, 97%]) (Fig 2A). A total of 17 studies were analyzed for PPV for all autosomal trisomies. Using the confirmed method, we found that the pooled PPV was 0.07 (95% CI: [0.04, 0.11] PI: 0.01, 0.31 I^2^ = 76% [61%, 85%]) (Fig 2B). Using an extended method, we found that the pooled PPV was 0.13 (95% CI: [0.08, 0.20] PI: 0.02, 0.54). Heterogeneity was high, I^2^ = 82% [73%, 85%] (Fig 2C). We also examined the proportion of false positives among all NIPT cases. The pooled FP rate among all NIPT cases was 0.0020 (95% CI: 0.0014, 0.0030] PI: 0.0005; 0.0088). Heterogeneity was high, I^2^ = 95%[93%,96%] (Fig 2D).

### 3.4 Proportion and PPV of GW-NIPT positive results for StrCAs

**Fig 3:**
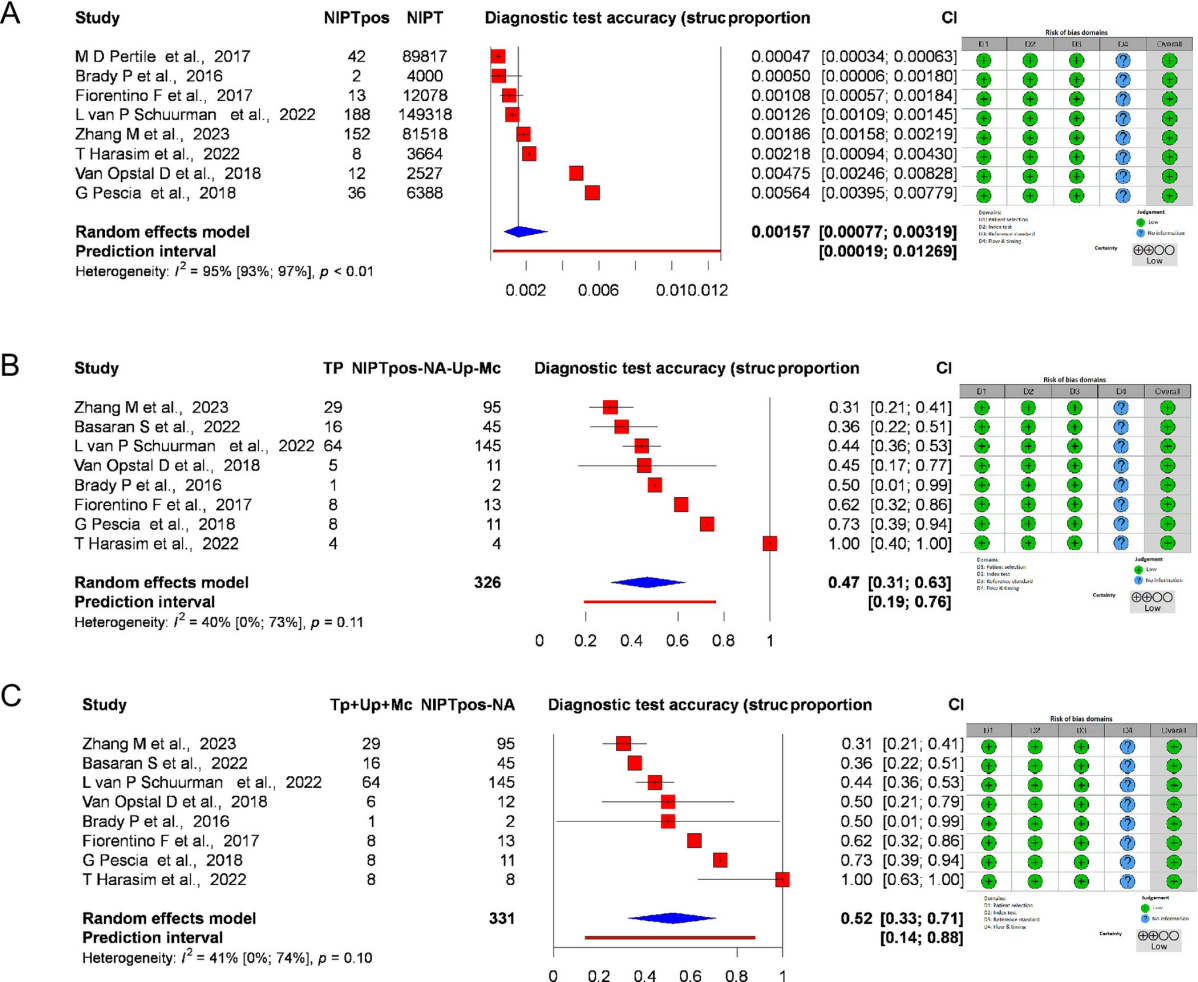
Forest plots representing the diagnostic test accuracy of StrCAs. A: Forest plot representing the pooled frequency of GW-NIPT positive results for StrCAs, B: Forest plot representing the PPV with a confirmed method for StrCAs, C: Forest plot representing the PPV with an extended method for StrCAs, (Abbreviations: NIPT: non-invasive prenatal test, Tp: true positive cases, Up: Ultrasound positive cases, Mc: Miscarriage, NA: not available).

StrCAs were investigated in 10 studies (20, 21, 24–26, 28–30, 32) with 388.357 cases, of which 593 were GW-NIPT positive. The pooled proportion was 0.00157 (95% CI: [0.00077, 0.00319] PI: 0.00019, 0–0.01269). Heterogeneity was high, I2 = 95% [93%, 97%] (Fig 3A). The pooled PPV for StrCAs using the strict method was 0.47 (95% CI: [0.31, 0.63] PI: 0.19, 0.76). Heterogeneity was I2 = 40% [0%, 73%] (Fig 3B). Using an extended method, we found that the pooled ratio was 0.52 (95% CI: [0.33, 0.71] PI: 0.14, 0.88). Heterogeneity was I2 = 41% [0%, 74%] (Fig 3C).

### 3.5 Distribution of chromosomal aberrations by each chromosome

The highest number of GW-NIPT positive results was obtained for chromosome 7 trisomy (n 480 = 480), with a pooled positive ratio among all the combined T positive results of 0.27 (95% CI: [0.22, 0.31] PI: 0.154, 0.43) (S26 Fig in S1 File). Our results show that T7 trisomy was followed by T16 (n = 199), with a random-effects pooled proportion of 0.11 (95% CI: [0.09, 0.1] PI: 0. 007, 0.18) (S34 Fig in S1 File), T8 (n = 169) with a random-effects pooled proportion of 0.09 (95% CI: [0.08, 0.12] PI: 0.05, 0.16) (S27 Fig in S1 File), T20 (n = 137) with a random-effects pooled proportion of 0.07 (95% CI: [0.05, 0.09] PI: 0.04,0.13) (S37 Fig in S1 File), T22 (n = 134) with a random-effects pooled proportion of 0.08 (95% CI: [0.065, 0.10] PI:0.04,0.16) (S38 Fig in S1 File), and T15 (n = 122) with a random-effects pooled proportion of 0.06 (95% CI: [0.04, 0.08] PI:0.017, 0.18) (S33 Fig in S1 File), which is considered more common.

Interestingly, T1, T17, and T19 GW-NIPT positivity was very rare. The random-effects pooled proportion for T1(n = 6) was 0.003 (95% CI: [0.00, 0.0] PI: 0.00, 0.03) (S20 Fig in S1 File); T17 (n = 8) with a random-effects pooled proportion of 0.00 (95% CI: [0.00, 0.01] PI: 0.00,0.13) (S35 Fig in S1 File) and T19 (n = 1) with a random-effects pooled proportion of 0.00 (95% CI: [0.00, 0.20] PI: 0.00, 0.34) (S36 Fig in S1 File).

**Fig 4:**
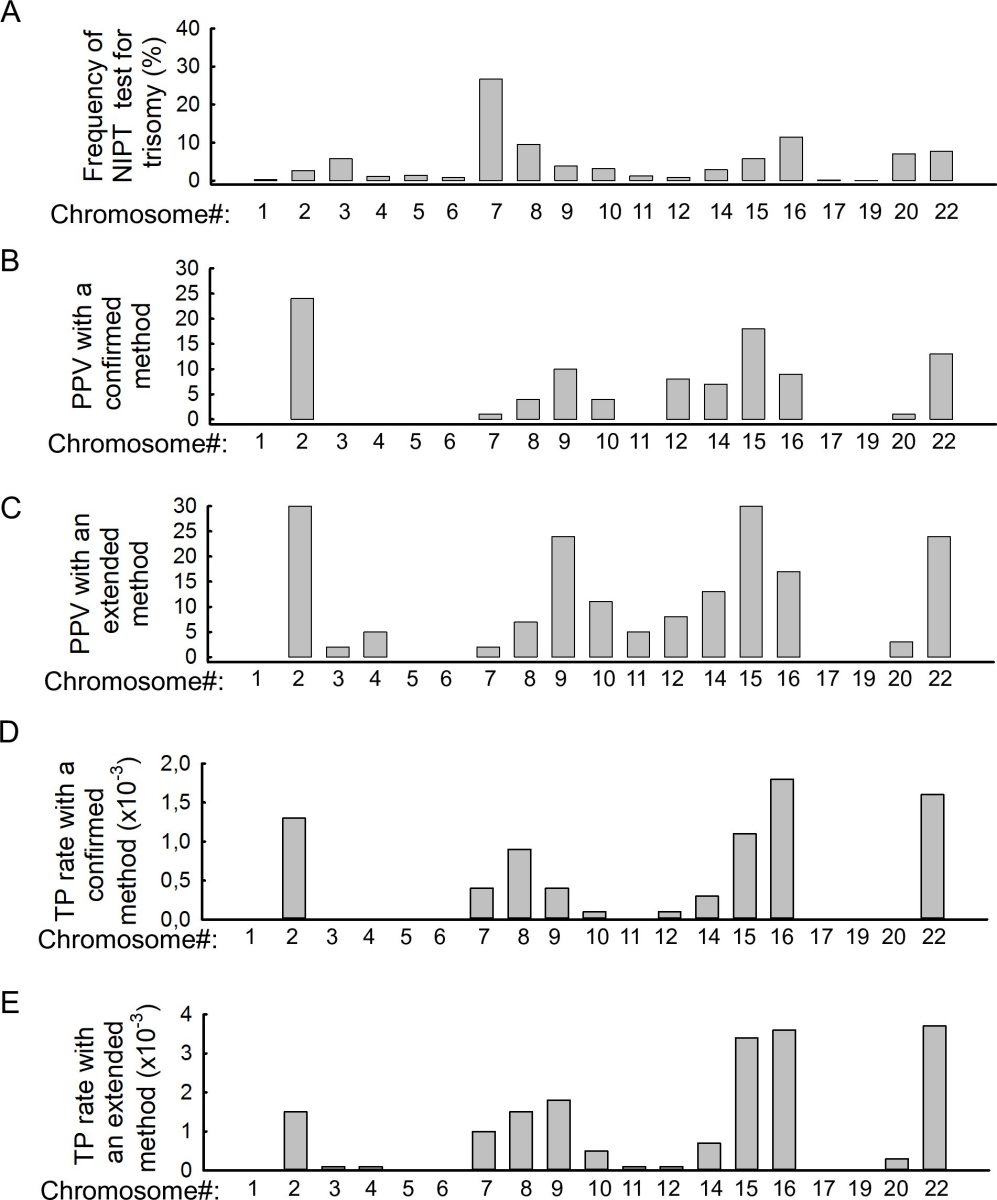
Column plot for visualization of the habitat of each chromosome. A: Column plot representing the pooled frequency of GW-NIPT positive results for all rare autosomal trisomies (RATs), B: Column plot representing the PPV with a confirmed method for all RATs, C: Column plot representing the PPV with an extended method for all RATs, D: Column plot representing the pooled true positive RAT rate, E: Column plot representing the pooled true positive RAT rate. (Abbreviations: NIPT: non-invasive prenatal test, TP: true positive cases, PPV: positive predictive value.

Of a total of 1,697 GW-NIPT RAT positive cases, 87 were true positives using the confirmed method and 174 with the extended method. With the confirmed method, the highest number of true positives was found in T16, with a random-effects pooled TP proportion among all NIPT cases of 1.7 x10^-5^ (95% CI: [0. 9x10^-5^, 3.0 x10^-5^] PI: 0. 9x10^-5^, 3.0x10^-5^), followed by T22, with a random-effects pooled proportion of 1.5 x10^-5^ (95% CI: [0. 8 x10^-5^, 2.8 x10^-5^] PI: 0. 8 x10^-5^, 2.9 x10^-5^), and T2, with a random-effects pooled proportion of 1.5 x10^-5^ (95% CI: [0.8 x10^-5^, 3.0 x10^-5^] PI: 0. 5 x10^-5^, 4.2 x10^-5^) (Fig 4D). With an extended method, the highest number of true positives was confirmed in T15, with a random-effects pooled proportion of 2.9 x10^-5^ (95% CI: [1.0 x10^-5^, 8.2 x10^-5^] PI: 0. 1 x10^-5^, 7,9 x10^-5^), followed by T16, with a random-effects pooled proportion of 3.5 x10^-5^ (95% CI: [2.3 x10^-5^, 5.2 x10^-5^] PI: 2.3 x10^-5^, 5.2 x10^-5^) and T22, with a random-effects pooled proportion of 3.2 x10^-5^ (95% CI: [1.3 x10^-5^, 7.9 x10^-5^] PI: 2.0 x10^-5^, 4.33 x10^-5^) (Fig 4E).

### 3.6 Positive predictive values per chromosome

The pooled PPV with the confirmed method was 0.07 (95% CI: [0.04, 0.11] PI: 0.01, 0.31). Heterogeneity was high, I^2^ = 76%[61%,85%], p<0.01 (Fig 4B), whereas with the extended methodology, it was 0.13 (95% CI: [0.08, 0.20]). Heterogeneity was high, I^2^ = 82% [73%,88%], p<0.01 (Fig 4C).

For StrCAs, follow-up was possible for 331 out of 498 positive cases. For GW-NIPT, 140 cases were true positive, and 191 cases were false positive. Two cases were aborted on ultrasonography and three cases due to intrauterine fetal death. The pooled PPV with the confirmed methodology (true positive cases: confirmed by genetic testing) was 0.47 (95% CI: [0.31, 0.63] PI: 0.19,0.76). Heterogeneity was I^2^ = 40% [0%,73%], p = 0.11 (Fig 3B), whereas with the extended methodology (true positive cases: confirmed by genetic testing, intrauterine fetal death, and termination of pregnancy for an ultrasound abnormality), it was 0.52 (95% CI: [0.33, 0.71] PI: 0.14, 0,88). Heterogeneity was, I^2^ = 41% [41%,74%] (Fig 3C). The pooled PPV for RAT varied from chromosome to chromosome.

For the confirmed methodology, the highest PPV was observed for T2 (24%, CI: 0.13, 0.42), followed by T15 (18%, CI: 0.04, 0.54). (Fig 4B). Using an extended methodology, we found the highest PPV for T15 (38%, CI: 0.18, 0.63), followed by T2 (30%, CI: 0.17, 0.46), T22 (25%, CI: 0.12, 0.44), T9 (24%, CI: 0.11, 0.46) and then T16 (17% CI: 0.12, 0.24) (Fig 4C).

As the number of true positive cases was the product of the GW-NIPT positive case count and the PPV, the true positive case count was again different. The most frequent actual positive rare trisomy cases were T15 (CI: 3.4 x10^-5^ [1.2 x10^-5^, 9.3 x10^-5^]), T22 (CI: 3.7 x10^-5^ [1.6 x10^-5^, 8.7 x10^-5^]), and T16 (CI: 3.6 x10^-5^ [2.4 x10^-5^, 5.5 x10^-5^]). Although the most common GW-NIPT positive was T7, only the 7th most common among the actually positive cases (Fig 4A, 4D and 4E). No true positive T1, T5, T6, T17, and T19 cases were found in our study (Fig 4D and 4E).

### 3.7 Assessment of risk of bias and level of certainty of evidence

A summary of the Quadas-2 risk of bias assessment is presented in S3 Table in S1 File. There was no bias in patient selection in the index tests. Flow and timing were not clear in all studies, as follow-up of cases was highly variable, with amniocentesis, ultrasound, and outcome follow-up.

We found significant publication bias in both cases. For the confirmed and extended methods, the p-values were 0.0023 and 0.0026, respectively. See also the funnel plots in S43 and S44 Figs in S1 File. It appears that the largest studies published smaller PPV results. Note also that the effect was milder for the extended method case. The effect of the study by Fiorentino et al. was quite large compared to the other studies. To exclude the possibility that this study distorted the results we repeated the analysis without this study. (Fiorentino, Bono et al. 2017). Although publication bias remained significant, the p-value increased somewhat (0.0151 and 0.0150, respectively.

All outcomes had low levels of evidence, due to the limited number of positive cases and considerable heterogeneity.

### 3.8 Individual results

Individual data from three studies could be statistically interpreted as true positive and false positive cases [19, 21, 27]. Individual analyses were performed to analyze maternal age and gestational age. In this case, we also applied the confirmed and extended methods described in Section 2.8. There were 23 true positive cases and 248 false positives using the confirmed method, whereas there were 27 true positive and 248 false positive cases with the extended method. The pooled mean difference in gestational age was -4.63 days (CI: -22.75, 13.49) for the confirmed method and -3.88 days (CI: -23.95, 16.19) for the extended method. The pooled mean difference in maternal age was 2.83 years (CI: -1.88, 7.54) for the confirmed method and 2.71 years (CI:1.57, 6.99) for the extended method. (S39-S41 Figs in S1 File) The mean GA was not significantly lower (pooled mean difference (confidence) in the true positive cases.

## 4. Discussion

This study is the first meta-analysis to examine GW-NIPT test results for individual chromosomes. NIPT testing is a growing field in the era of prenatal diagnostics. cfDNA-based technologies were introduced in 2011 and until the GW-NIPT tests became available, only the most common trisomies (T21, T13, and T18) and sex chromosome abnormalities were investigated. Thus, their accuracy and specificity are now supported by a large number of studies in the current literature [32]. For common trisomies (T13, T18 and T21), the detection rate (DR) is high (>97.5%) and the false positive rate is low at 0.04% [29]. Next-generation sequencing (NGS) technologies have made a big difference in NIPT testing, achieving 99% sensitivity and specificity [34].

Currently, little data are available on the specificity, sensitivity, and PPV of testing for chromosomal aberrations in rare autosomal aneuploidies. Therefore, in this study, we aimed to collect the most relevant knowledge on some chromosomal abnormalities published in the literature so far, which could be of major help in genetic counselling in the future.

The PPV for rare chromosomal abnormalities in previous studies was mainly based on the pooled value. Its value varied between 6 and 58.8% [35]. In GW-NIPT studies, RAT cases represent a major challenge in genetic counselling.

Our study shows that for RATs, most of the abnormal GW-NIPT test results were for trisomy 7, but the true positive rate was negligible and in most cases, the GW-NIPT result was false positive (Fig 4A, 4D and 4E). As a consequence, the PPV value for chromosome 7 was very low. Of the 480 GW-NIPT cases collected, true positivity was confirmed in four cases with the confirmed method and in 10 cases with the extended method (S7a, S7b Fig in S1 File). Trisomy 7 is extremely rare at birth and is usually considered lethal in embryogenesis. All surviving children are mosaics with variable and nonspecific clinical features [36]. Similarly, according to data from recently published papers that are not part of our meta-analysis, most GW-NIPT-positive cases were found in T7. None of these cases confirmed true positivity, so the calculated PPV according to their data was zero, but the overall incidence of adverse perinatal events such as intrauterine growth failure, preterm birth, perinatal maladaptation, and other–placental function-related—pregnancy complications was 42.11% [32]–these may be the consequence of placental mosaicism and should be considered as a placental disease.

In addition, we found the highest number of true positive cases and the highest true positive rate for T16 (n = 18 with the confirmed methodology, n = 32 with extended methodology). The incidence of trisomy 16 in all registered pregnancies was approximately 1.5% [37], although this was significantly higher than the value calculated from our meta-analysis, which was only 0.0046%. Trisomy 16 is the most common chromosomal abnormality found in early miscarriages. Full trisomy 16 is incompatible with life and affected fetuses typically do not survive beyond the first trimester [38]. According to the literature, complete trisomy 16 is responsible for about 6% of miscarriages between weeks 8 and 15 [39]. In contrast, the mosaic form of trisomy 16 is compatible with life; therefore, the presence of a mosaic form of trisomy 16 is a major challenge in genetic counselling. Clinical symptoms of mosaic trisomy 16 can include intellectual disability, delayed growth and development, facial abnormalities, congenital heart defects, skeletal abnormalities, low muscle tone, poor nutrition, seizures, hearing loss, and vision problems [37], but the severity of clinical symptoms depends on the degree of mosaicism. Recent literature suggests that 66% of prenatally diagnosed cases are born alive, of which 93% survive the neonatal period. Eleven percent of pregnancies ended in intrauterine death (IUD), whereas 22% of pregnancies were terminated [37]. After chromosome 16, the second highest number of confirmed chromosomal trisomy cases in our cohort was chromosome 22 (Fig 4D and 4E). T22 has no ultrasound abnormalities in the first trimester, with characteristic abnormalities only appearing in the second and third trimesters, which may include IUGR, increased nuchal translucency, congenital heart defects, hydrocephaly, hydrothorax, and even cleft lip and palate [40]. As trisomy 22 cannot be detected by imaging before the second trimester, GW-NIPT is of great importance in identifying these cases, aiding early detection and pregnancy management [37].

On the basis of our meta-analysis data, it is very striking that for chromosome 15, the pooled PPV value was almost 18% when following the strict methodology, whereas it was almost 38% when using the less strict methodology (Fig 4B and 4C). Of the 122 NIPT positives, only 10 cases were confirmed as true positives, 6 cases were aborted due to abnormal ultrasound images, 19 cases ended in spontaneous abortion, and 11 cases had no information on the outcome (Table 2). This would suggest that T15 cases almost certainly end in miscarriage, so it is very likely that this will occur in many cases between the time the NIPT test is performed and the time the test result is announced. In addition, the rate of false positives was also very high, but further comprehensive analysis is needed to determine the significance of the possible underlying placental mosaicism.

**Table 2:** Number of alterations detected.

	Number of NIPT positive	Number of FP	Number of TP	Number of US	Number of Mis	Number of NA
**Chr1**	6	3	0	0	0	3
**Chr2**	48	31	10	2	1	4
**Chr3**	116	98	4	1	1	12
**Chr4**	22	21	0	0	1	0
**Chr5**	25	21	0	0	0	4
**Chr6**	15	14	0	0	0	1
**Chr7**	480	423	7	2	1	47
**Chr8**	169	150	7	2	2	8
**Chr9**	71	48	6	4	5	8
**Chr10**	55	44	4	1	1	5
**Chr11**	23	19	0	1	0	3
**Chr12**	15	10	3	0	0	2
**Chr14**	51	42	3	1	2	3
**Chr15**	122	76	10	6	19	11
**Chr16**	199	156	18	4	10	11
**Chr17**	8	7	0	0	0	1
**Chr19**	1	1	0	0	0	0
**Chr20**	137	116	1	1	1	18
**Chr22**	134	92	14	4	14	10
**∑**	**1697**	**1372**	**87**	**29**	**58**	**151**

Abbreviations: FP–False positive cases; TP–True positive cases; US–Ultrasound positive cases; Mis–miscarriages; NA–non-available)

Our data show that there are no genetically verified true positive cases for chromosomes 1, 4, 5, 6, 11, 17, and 19, only miscarriages, or abnormal ultrasound findings, so these chromosomal abnormalities all lead to premature fetal death (Fig 4D and 4E).

For individual analyses, the results obtained are not statistically significant and are highly limited due to the small number of cases. The relationships between maternal age, gestational age, and false and true positivity require further investigation using much more data. (S39-S42 Figs in S1 File).

There is a large amount of data available on common autosomal trisomies and sex chromosomal aneuploidies; the frequency, specificity, sensitivity, and PPV are relatively well-defined. For rare autosomal trisomies, data for individual chromosomes are not available. Prospective and retrospective studies do not include enough cases to be statistically meaningful for RATs. In the present meta-analysis, we aimed to solve this problem by analyzing a large number of cases. As the sensitivity and specificity of very rare cases are difficult to interpret, we used frequency values and PPVs in this study. The primary aim of GW-NIPT tests is to detect fetal chromosomal abnormalities. Although all NIPT tests have relatively low percentages of RAT positivity and even lower percentages of true positives, our results suggest that it is worth considering performing GW-NIPT in the clinical setting. In particular, in cases where no or only non-specific ultrasound abnormalities are detected in the first trimester (e.g., T22), GW-NIPT is the only option for early detection of genetic abnormalities. This may provide a satisfactory answer to the often-questionable opinions about the usefulness of GW-NIPT and supports its place in prenatal diagnostics. Sequencing tests are very efficient, so false positive NIPT results are most likely to be due to biological factors, such as localized mosaicism in the placenta, rather than technical problems. A number of studies have shown that placental mosaicism increases the incidence of certain pregnancy complications such as preeclampsia, intrauterine growth failure and preterm birth [41]. Therefore, in cases when a positive NIPT test cannot be confirmed by invasive testing, placental mosaicism should be suspected in the first instance and the pregnant woman should be referred to closer obstetric care. Some studies have shown that positive NIPT results increase the risk not only of chromosomal abnormalities but also of other genetic abnormalities such as uniparental disomy.

### 4.1 Principal findings

This is the first study to provide single results for rare GW-NIPT findings with a large number of cases. The clinical implementation of GW-NIPT testing was based on the societal need to provide patients with more and more genetic information about the fetus. Testing for RATs and StrCAs not only increases the efficiency of screening for abnormal pregnancies, but also the number of false positive cases. Our study provides individualized data for each chromosome, allowing for more accurate genetic counselling and prediction of pregnancy outcomes, which can have a significant impact on prenatal care.

### 4.2 Implications for practice and research

This meta-analysis has determined the frequency of rare chromosomal abnormalities, test-positive rates, and the PPV of each chromosomal abnormality with high accuracy. These data are very important for pre- and post-test consultation and patient information leaflets. It is crucial that new scientific findings are translated into clinical routine as quickly as possible [42, 43].

With the development of GW-NIPT technology and the higher number of cases, tests are detecting in an increasing number of rare chromosomal abnormalities, which are causing many difficulties in genetic counselling and in pregnancy care. Although the 2022 American College of Medical Genetics and Genomics guidelines on NIPT state that there is not yet sufficient evidence to support the routine use of GW-NIPT [44], recent literature suggests that NIPT abnormalities may be associated with three main factors, such as fetal abnormality [3], placental causes [4], and maternal origin [5]. The detection of fetal abnormalities leading to miscarriage can provide valuable insights into the etiology of pregnancy loss and contribute to the development of more effective strategies for miscarriage prevention. By identifying these anomalies, we can better understand the genetic factors underlying miscarriage. Knowledge of all these may help to design a personalized reproductive medicine therapy for the couple concerned. Similarly, the knowledge of adverse pregnancy complications can help to support prenatal care, because the confirmed placental mosaicism increases the risk of IUGR, preterm birth, and preeclampsia; therefore, our results suggest that if NIPT abnormality is not confirmed by amniocentesis, it should be managed as suspected placental mosaicism, and therefore a more rigorous prenatal care is recommended. Finally, the most common causes of maternal origin are maternal mosaicism, maternal tumors, and maternal copy number variations. Cytogenetic testing of pregnant women is recommended to detect maternal mosaicism and balanced translocations. An onco-hematological consultation is recommended to exclude tumor diseases during pregnancy.

### 4.3 Strengths and limitations

A limitation of this analysis was the high heterogeneity, with GW-NIPT tests being widely available only from data of countries with higher GDP, so global prevalence could not be calculated. The high heterogeneity might be explained by the inverse relationship between population size and TP effect in the populations studied. This may be due to the fact that the higher the cohort size is, the lower the rate of follow-up is. (S43-S46 Figs in S1 File) Maternal age higher than average should also influence the results.

The strength of the study lies in the fact that we used a parallel protocol, which was both confirmed and extended. No language restrictions were used. Because of the large number of NIPT results in the literature, our data on each chromosome could be used to make recommendations to support clinical decision-making, which might lead to changes in the guidelines currently used. The current highest analysis of GW-NIPT samples examines rare chromosomal abnormalities on a chromosome-by-chromosome basis and includes analysis of individual data.

## 5. Conclusion

Screening with GW-NIPT can help in the early detection of rare chromosomal abnormalities. GW-NIPT has a minimal increase in the false positive rate but can detect any chromosomal abnormality where the size of the mutation exceeds the limit of detection. With the increasing availability of GW-NIPT tests, the detection of rare chromosomal abnormalities poses a particular challenge for the interpretation of test results and appropriate genetic counselling. Confirmation of a positive NIPT result with an invasive diagnostic test is key to verifying fetal status. This can be performed from chorionic villus sampling or amniocentesis, of which amniocentesis provides a more robust result for the fetus. A high percentage of NIPT RAT cases are not confirmed in the fetus, but these cases are largely due to placental mosaicism. Women with confirmed placental mosaicism have an increased risk of IUGR, preterm birth, and preeclampsia. Our results suggest that if the NIPT abnormality is not confirmed by amniocentesis, it should be managed as suspected placental mosaicism, and therefore more rigorous prenatal care is recommended (e.g., ultrasonography every two weeks to check for IUGR and other placental abnormalities). Genetic counselling for RAAs during prenatal screening should include the possibility of growth restriction, mosaicism (fetal and confirmed placental), and uniparental disomy. In the rare case of mosaic trisomy, when first-trimester ultrasonography shows no significant abnormality, GW-NIPT may be useful for early detection, e.g., in T22. Screening positivity and the frequency of PPV vary widely between chromosomes. Accurate knowledge of the risk can help healthcare professionals in the post-test consultation and patients in the decision-making process.

## Supporting information

S1 Checklist PRISMA checklist. (DOCX)

S1 File (DOCX)
