# Supplementary material for: Genome-Wide, Non-Invasive Prenatal Testing for rare chromosomal abnormalities: A systematic review and meta-analysis of diagnostic test accuracy
Source: PLoS One. 2024 Nov 5;19(11):e0308008. doi: 10.1371/journal.pone.0308008 (PMC11537411; doi:10.1371/journal.pone.0308008)

**SUPPLEMENTARY MATERIAL**

**TABLE OF CONTENT**

**Figure S1a.** Forest plots representing the accuracy of NIPT test for trisomy chromosome 1 using a confirmed method. The confirmed methodology is to consider only what has been confirmed by genetic testing as a true positive case.

**Figure S1b.** Forest plots representing the accuracy of NIPT test for trisomy chromosome 1 using an extended method. The extended methodology is to consider as true positive cases, in addition to those confirmed by genetic testing, intrauterine fetal death and cases terminated by ultrasound.

**Figure S2a**. Forest plots representing the accuracy of NIPT test for trisomy chromosome 2 using a confirmed method. The confirmed methodology is to consider only what has been confirmed by genetic testing as a true positive case.

**Figure S2b.** Forest plots representing the accuracy of NIPT test for trisomy chromosome 2 using an extended method. The extended methodology is to consider as true positive cases, in addition to those confirmed by genetic testing, intrauterine fetal death and cases terminated by ultrasound.

**Figure S3a.** Forest plots representing the accuracy of NIPT test for trisomy chromosome 3 using a confirmed method. The confirmed methodology is to consider only what has been confirmed by genetic testing as a true positive case.

**Figure S3b.** Forest plots representing the accuracy of NIPT test for trisomy chromosome 3 using an extended method. The extended methodology is to consider as true positive cases, in addition to those confirmed by genetic testing, intrauterine fetal death and cases terminated by ultrasound.

**Figure S4a**. Forest plots representing the accuracy of NIPT test for trisomy chromosome 4 using a confirmed method. The confirmed methodology is to consider only what has been confirmed by genetic testing as a true positive case.

**Figure S4b.** Forest plots representing the accuracy of NIPT test for trisomy chromosome 4 using an extended method. The extended methodology is to consider as true positive cases, in addition to those confirmed by genetic testing, intrauterine fetal death and cases terminated by ultrasound.

**Figure S5a.** Forest plots representing the accuracy of NIPT test for trisomy chromosome 5 using a confirmed method. The confirmed methodology is to consider only what has been confirmed by genetic testing as a true positive case.

**Figure S5b.** Forest plots representing the accuracy of NIPT test for trisomy chromosome 5 using an extended method. The extended methodology is to consider as true positive cases, in addition to those confirmed by genetic testing, intrauterine fetal death and cases terminated by ultrasound.

**Figure S6a.** Forest plots representing the accuracy of NIPT test for trisomy chromosome 6 using a confirmed method. The confirmed methodology is to consider only what has been confirmed by genetic testing as a true positive case.

**Figure S6b.** Forest plots representing the accuracy of NIPT test for trisomy chromosome 6 using an extended method. The extended methodology is to consider as true positive cases, in addition to those confirmed by genetic testing, intrauterine fetal death and cases terminated by ultrasound.

**Figure S7a.** Forest plots representing the accuracy of NIPT test for trisomy chromosome 7 using a confirmed method. The confirmed methodology is to consider only what has been confirmed by genetic testing as a true positive case.

**Figure S7b.** Forest plots representing the accuracy of NIPT test for trisomy chromosome 7 using an extended method. The extended methodology is to consider as true positive cases, in addition to those confirmed by genetic testing, intrauterine fetal death and cases terminated by ultrasound.

**Figure S8a.** Forest plots representing the accuracy of NIPT test for trisomy chromosome 8 using a confirmed method. The confirmed methodology is to consider only what has been confirmed by genetic testing as a true positive case.

**Figure S8b.** Forest plots representing the accuracy of NIPT test for trisomy chromosome 8 using an extended method. The extended methodology is to consider as true positive cases, in addition to those confirmed by genetic testing, intrauterine fetal death and cases terminated by ultrasound.

**Figure S9a.** Forest plots representing the accuracy of NIPT test for trisomy chromosome 9 using a confirmed method. The confirmed methodology is to consider only what has been confirmed by genetic testing as a true positive case.

**Figure S9b.** Forest plots representing the accuracy of NIPT test for trisomy chromosome 9 using an extended method. The extended methodology is to consider as true positive cases, in addition to those confirmed by genetic testing, intrauterine fetal death and cases terminated by ultrasound.

**Figure S10a.** Forest plots representing the accuracy of NIPT test for trisomy chromosome 10 using a confirmed method. The confirmed methodology is to consider only what has been confirmed by genetic testing as a true positive case.

**Figure S10b.** Forest plots representing the accuracy of NIPT test for trisomy chromosome 10 using an extended method. The extended methodology is to consider as true positive cases, in addition to those confirmed by genetic testing, intrauterine fetal death and cases terminated by ultrasound.

**Figure S11a.** Forest plots representing the accuracy of NIPT test for trisomy chromosome 11 using a confirmed method. The confirmed methodology is to consider only what has been confirmed by genetic testing as a true positive case.

**Figure S11b.** Forest plots representing the accuracy of NIPT test for trisomy chromosome 11 using an extended method. The extended methodology is to consider as true positive cases, in addition to those confirmed by genetic testing, intrauterine fetal death and cases terminated by ultrasound.

**Figure S12a.** Forest plots representing the accuracy of NIPT test for trisomy chromosome 12 using a confirmed method. The confirmed methodology is to consider only what has been confirmed by genetic testing as a true positive case.

**Figure S12b.** Forest plots representing the accuracy of NIPT test for trisomy chromosome 12 using an extended method. The extended methodology is to consider as true positive cases, in addition to those confirmed by genetic testing, intrauterine fetal death and cases terminated by ultrasound.

**Figure S13a.** Forest plots representing the accuracy of NIPT test for trisomy chromosome 14 using a confirmed method. The confirmed methodology is to consider only what has been confirmed by genetic testing as a true positive case.

**Figure S13b.** Forest plots representing the accuracy of NIPT test for trisomy chromosome 14 using an extended method. The extended methodology is to consider as true positive cases, in addition to those confirmed by genetic testing, intrauterine fetal death and cases terminated by ultrasound.

**Figure S14a.** Forest plots representing the accuracy of NIPT test for trisomy chromosome 15 using a confirmed method. The confirmed methodology is to consider only what has been confirmed by genetic testing as a true positive case.

**Figure S14b.** Forest plots representing the accuracy of NIPT test for trisomy chromosome 15 using an extended method. The extended methodology is to consider as true positive cases, in addition to those confirmed by genetic testing, intrauterine fetal death and cases terminated by ultrasound.

**Figure S15a.** Forest plots representing the accuracy of NIPT test for trisomy chromosome 16 using a confirmed method. The confirmed methodology is to consider only what has been confirmed by genetic testing as a true positive case.

**Figure S15b.** Forest plots representing the accuracy of NIPT test for trisomy chromosome 16 using an extended method. The extended methodology is to consider as true positive cases, in addition to those confirmed by genetic testing, intrauterine fetal death and cases terminated by ultrasound.

**Figure S16a.** Forest plots representing the accuracy of NIPT test for trisomy chromosome 17 using a confirmed method. The confirmed methodology is to consider only what has been confirmed by genetic testing as a true positive case.

**Figure S16b.** Forest plots representing the accuracy of NIPT test for trisomy chromosome 17 using an extended method. The extended methodology is to consider as true positive cases, in addition to those confirmed by genetic testing, intrauterine fetal death and cases terminated by ultrasound.

**Figure S17a.** Forest plots representing the accuracy of NIPT test for trisomy chromosome 19 using a confirmed method. The confirmed methodology is to consider only what has been confirmed by genetic testing as a true positive case.

**Figure S17b.** Forest plots representing the accuracy of NIPT test for trisomy chromosome 19 using an extended method. The extended methodology is to consider as true positive cases, in addition to those confirmed by genetic testing, intrauterine fetal death and cases terminated by ultrasound.

**Figure S18a.** Forest plots representing the accuracy of NIPT test for trisomy chromosome 20 using a confirmed method. The confirmed methodology is to consider only what has been confirmed by genetic testing as a true positive case.

**Figure S18b.** Forest plots representing the accuracy of NIPT test for trisomy chromosome 20 using an extended method. The extended methodology is to consider as true positive cases, in addition to those confirmed by genetic testing, intrauterine fetal death and cases terminated by ultrasound.

**Figure S19a.** Forest plots representing the accuracy of NIPT test for trisomy chromosome 22 using a confirmed method. The confirmed methodology is to consider only what has been confirmed by genetic testing as a true positive case.

**Figure S19b.** Forest plots representing the accuracy of NIPT test for trisomy chromosome 22 using an extended method. The extended methodology is to consider as true positive cases, in addition to those confirmed by genetic testing, intrauterine fetal death and cases terminated by ultrasound.

**Figure S20.** Forest plots representing the frequency of NIPT test for trisomy chromosome 1.

**Figure S21.** Forest plots representing the frequency of NIPT test for trisomy chromosome 2.

**Figure S22.** Forest plots representing the frequency of NIPT test for trisomy chromosome 3.

**Figure S23.** Forest plots representing the frequency of NIPT test for trisomy chromosome 4.

**Figure S24.** Forest plots representing the frequency of NIPT test for trisomy chromosome 5.

**Figure S25.** Forest plots representing the frequency of NIPT test for trisomy chromosome 6.

**Figure S26.** Forest plots representing the frequency of NIPT test for trisomy chromosome 7.

**Figure S27.** Forest plots representing the frequency of NIPT test for trisomy chromosome 8.

**Figure S28.** Forest plots representing the frequency of NIPT test for trisomy chromosome 9.

**Figure S29.** Forest plots representing the frequency of NIPT test for trisomy chromosome 10.

**Figure S30.** Forest plots representing the frequency of NIPT test for trisomy chromosome 11.

**Figure S31.** Forest plots representing the frequency of NIPT test for trisomy chromosome 12.

**Figure S32.** Forest plots representing the frequency of NIPT test for trisomy chromosome 14.

**Figure S33.** Forest plots representing the frequency of NIPT test for trisomy chromosome 15.

**Figure S34.** Forest plots representing the frequency of NIPT test for trisomy chromosome 16.

**Figure S35.** Forest plots representing the frequency of NIPT test for trisomy chromosome 17.

**Figure S36.** Forest plots representing the frequency of NIPT test for trisomy chromosome 19.

**Figure S37.** Forest plots representing the frequency of NIPT test for trisomy chromosome 20.

**Figure S38.** Forest plots representing the frequency of NIPT test for trisomy chromosome 22.

**Figure S39.** Forest plot for individual analysis for gestational age at sampling with confirmed methods

**Figure S40.** Forest plot for individual analysis for gestational age at sampling with extended methods

**Figure S41.** Forest plot for individual analysis for maternal age at sampling with confirmed methods

**Figure S42.** Forest plot for individual analysis for maternal age at sampling with extended methods

**Figure S43.** Linear regression test of funnel plot asymmetry for confirmed method

**Figure S44.** Linear regression test of funnel plot asymmetry for confirmed method without biggest outlyer

**Figure S45.** Linear regression test of funnel plot asymmetry for extended method

**Figure S46.** Linear regression test of funnel plot asymmetry for confirmed method without the largest outlier

**Table S1.** Eligibility criteria of each included studies

**Table S2.** Intervention and diagnostics in each group

**Table S3.** Risk of bias assessment using the Risk of Bias Quadas-2 tool

**Figure S1a.** Forest plots representing the accuracy of NIPT test for trisomy chromosome 1 using a confirmed method. The confirmed methodology is to consider only what has been confirmed by genetic testing as a true positive case.


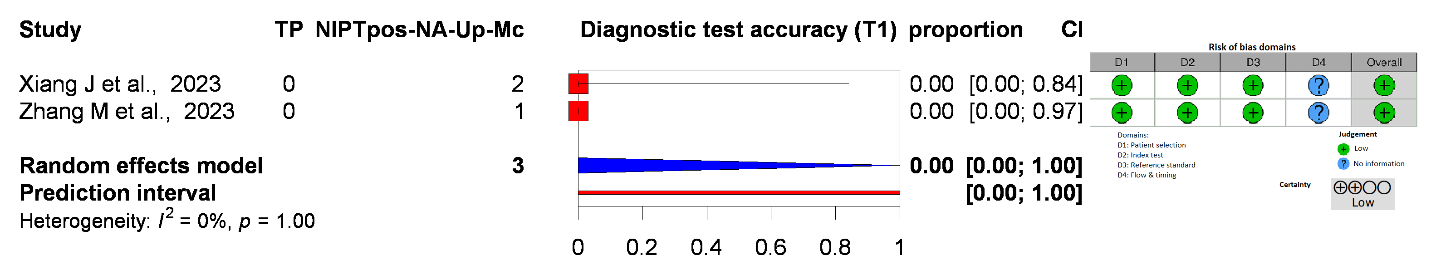


**Figure S1b.** Forest plots representing the accuracy of NIPT test for trisomy chromosome 1 using an extended method. The extended methodology is to consider as true positive cases, in addition to those confirmed by genetic testing, intrauterine fetal death and cases terminated by ultrasound.


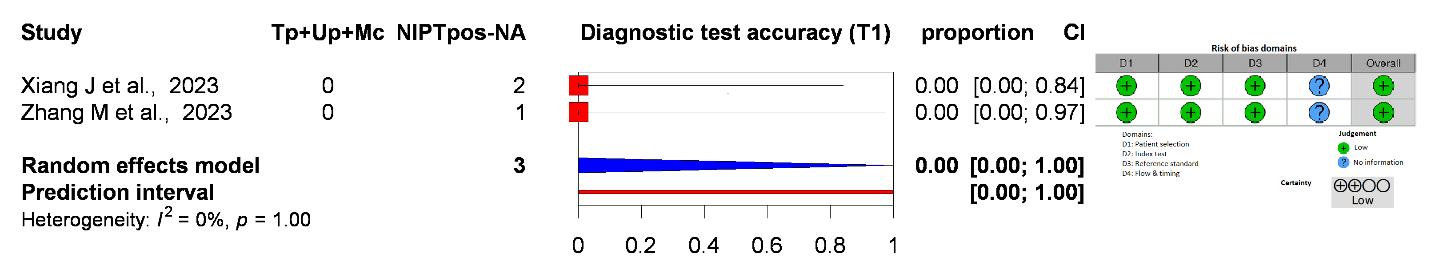


**Figure S2a.** Forest plots representing the accuracy of NIPT test for trisomy chromosome 2 using a confirmed method. The confirmed methodology is to consider only what has been confirmed by genetic testing as a true positive case.


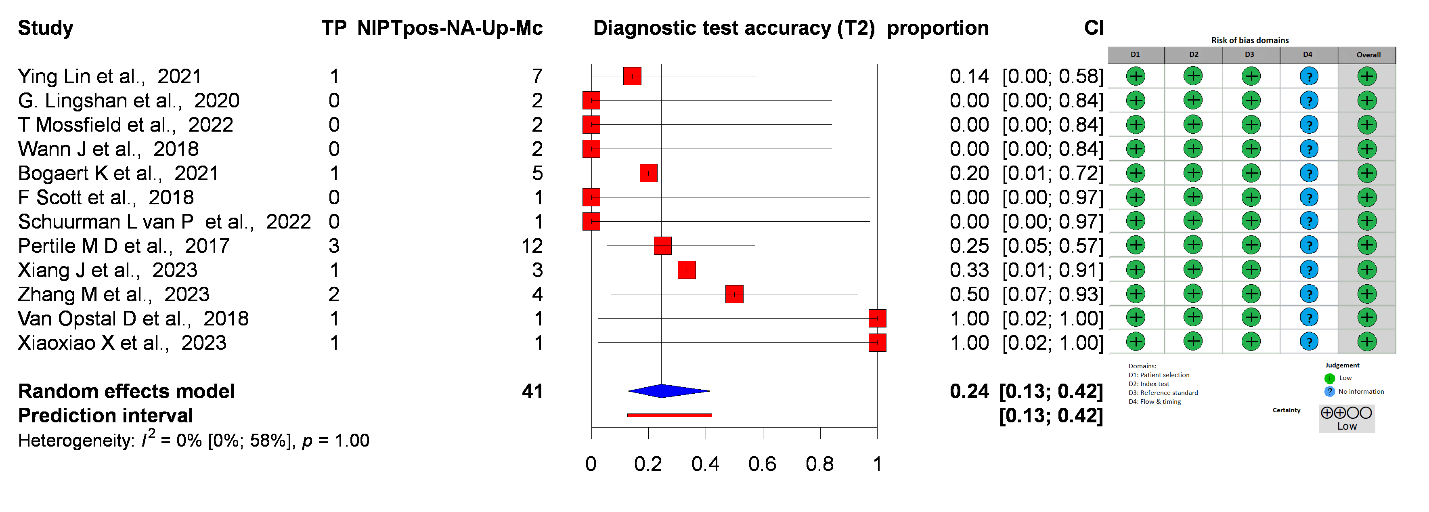


**Figure S2b.** Forest plots representing the accuracy of NIPT test for trisomy chromosome 2 using an extended method. The extended methodology is to consider as true positive cases, in addition to those confirmed by genetic testing, intrauterine fetal death and cases terminated by ultrasound.


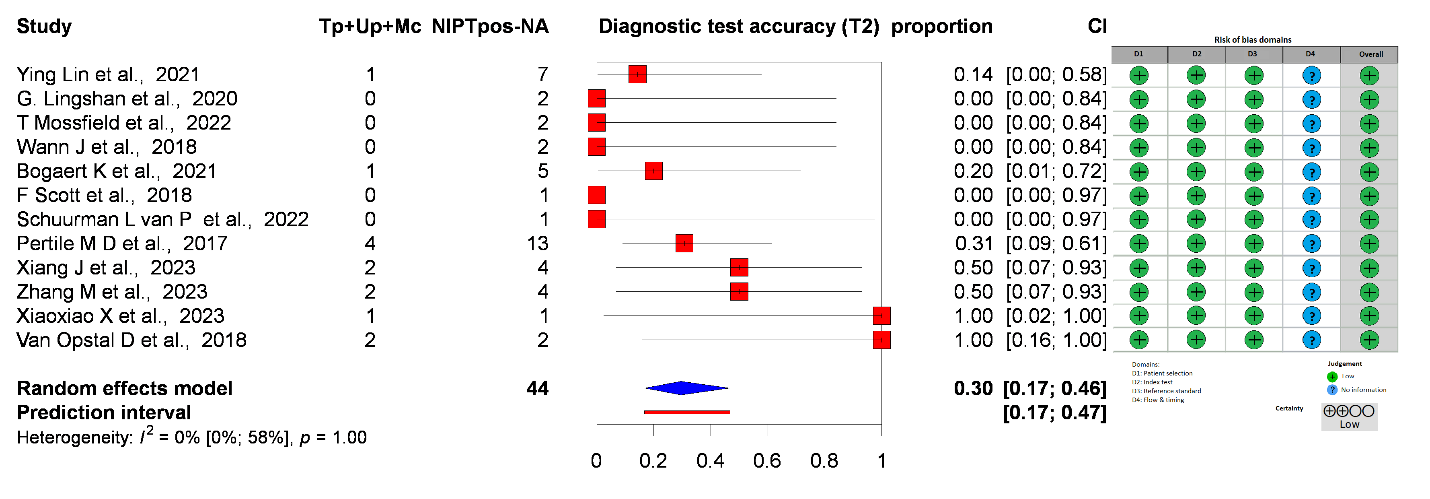


**Figure S3a.** Forest plots representing the accuracy of NIPT test for trisomy chromosome 3 using a confirmed method. The confirmed methodology is to consider only what has been confirmed by genetic testing as a true positive case.


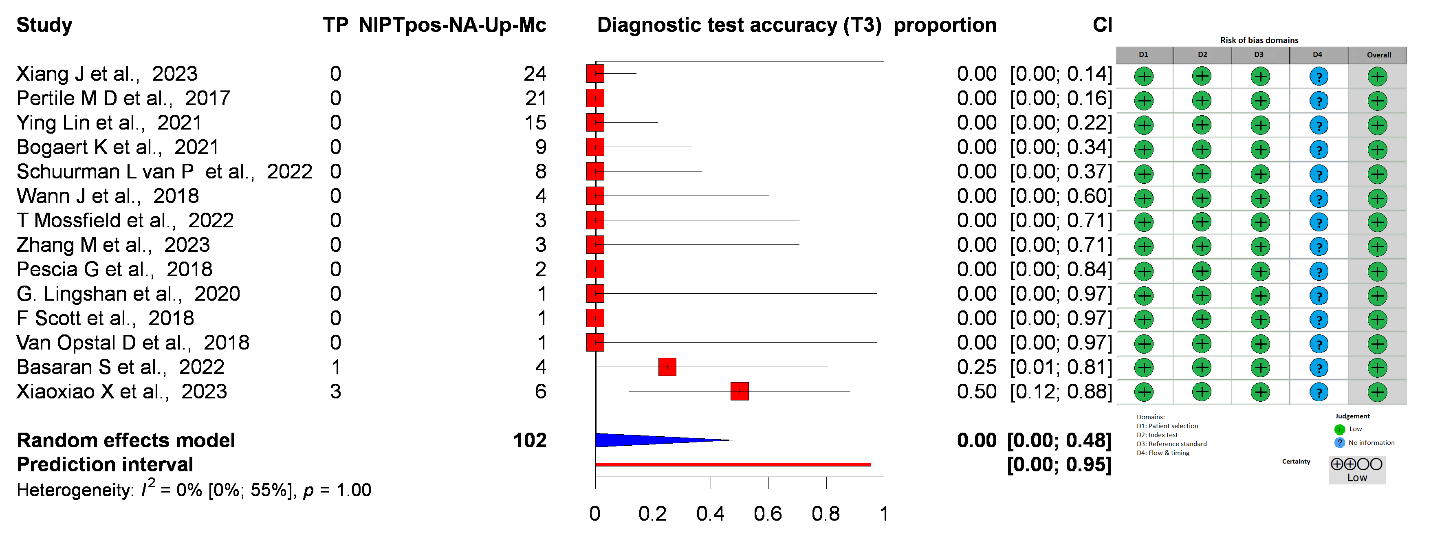


**Figure S3b.** Forest plots representing the accuracy of NIPT test for trisomy chromosome 3 using an extended method. The extended methodology is to consider as true positive cases, in addition to those confirmed by genetic testing, intrauterine fetal death and cases terminated by ultrasound.


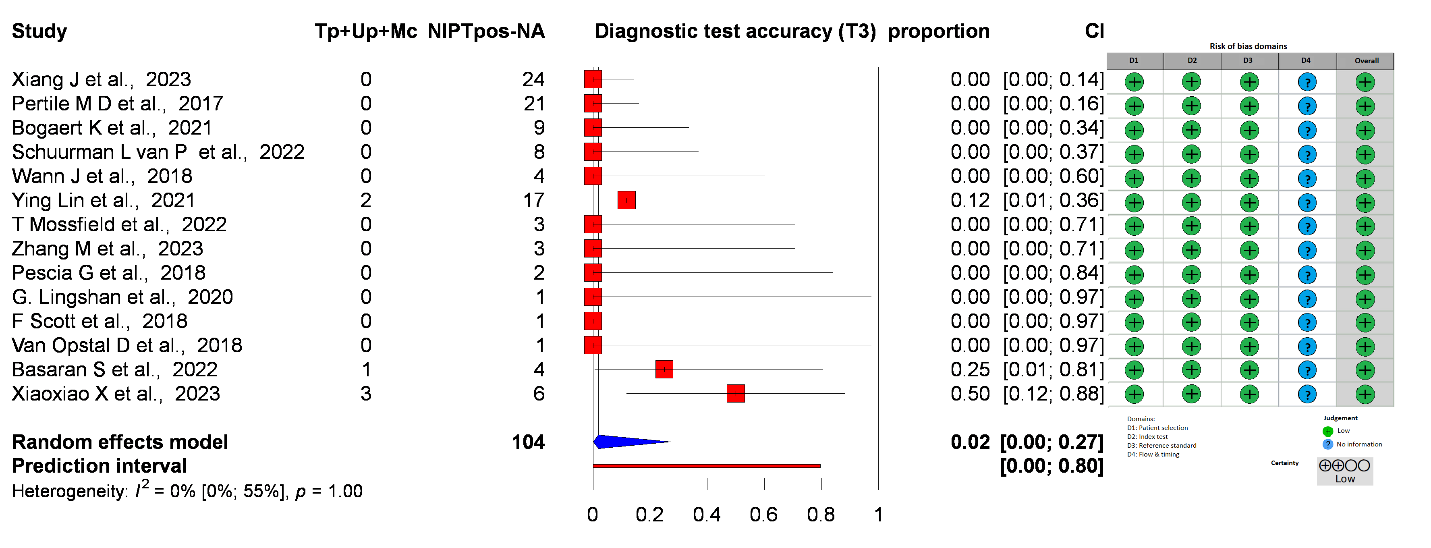


**Figure S4a.** Forest plots representing the accuracy of NIPT test for trisomy chromosome 4 using a confirmed method. The confirmed methodology is to consider only what has been confirmed by genetic testing as a true positive case.


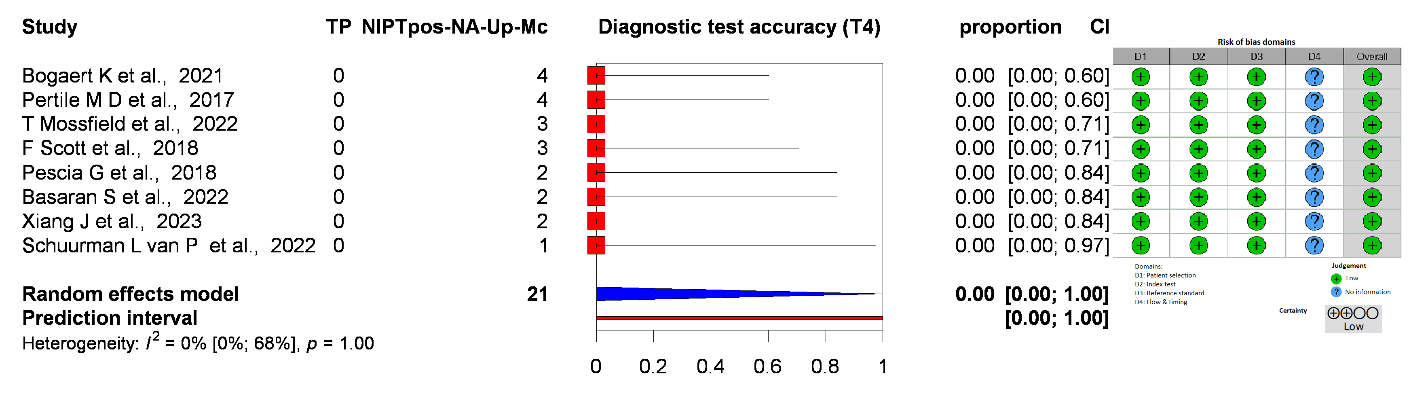


**Figure S4b.** Forest plots representing the accuracy of NIPT test for trisomy chromosome 4 using an extended method. The extended methodology is to consider as true positive cases, in addition to those confirmed by genetic testing, intrauterine fetal death and cases terminated by ultrasound.


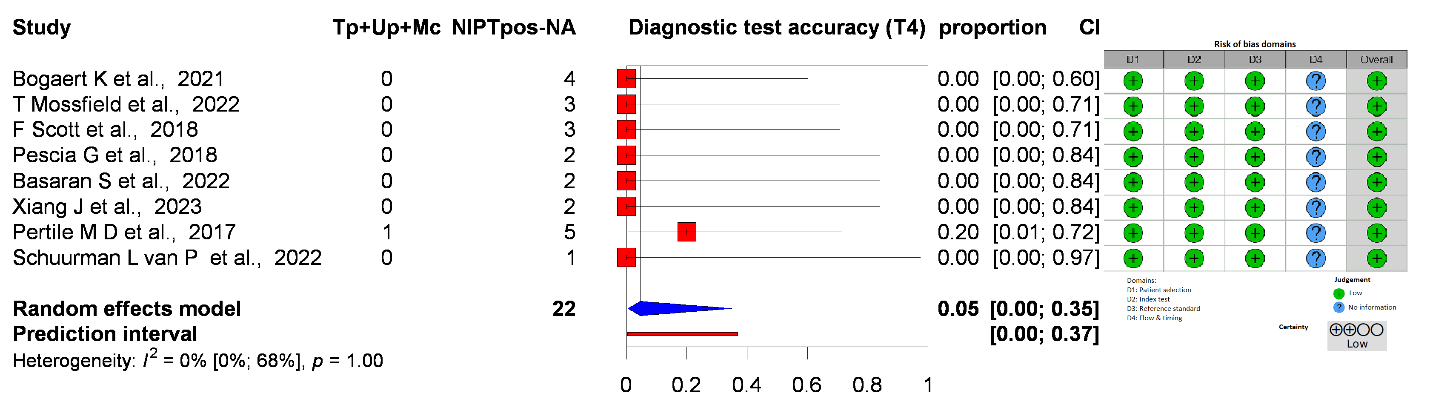


**Figure S5a.** Forest plots representing the accuracy of NIPT test for trisomy chromosome 5 using a confirmed method. The confirmed methodology is to consider only what has been confirmed by genetic testing as a true positive case.


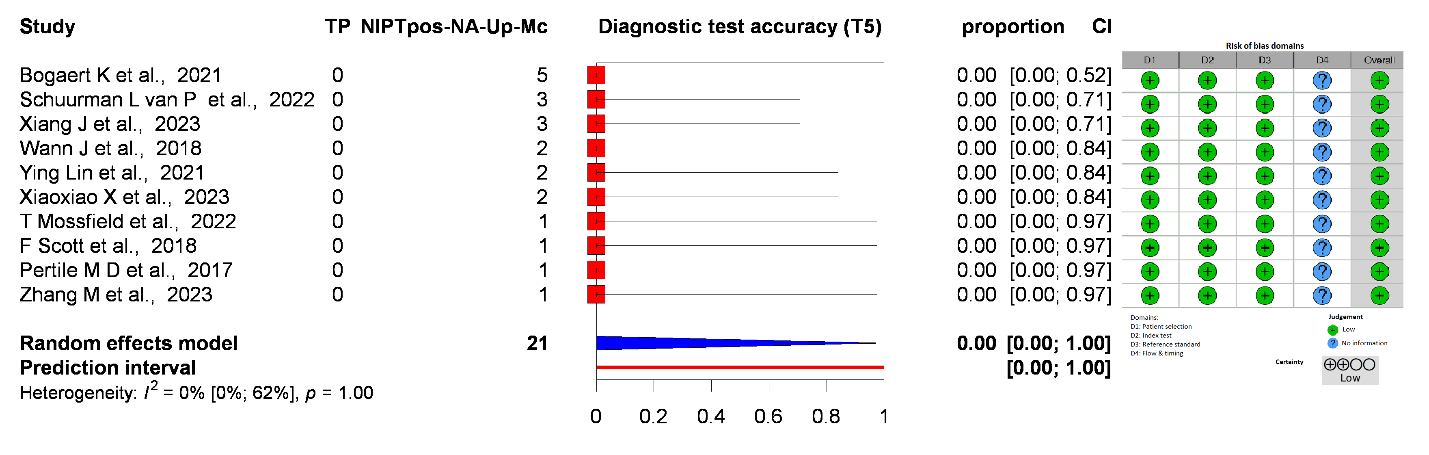


**Figure S5b.** Forest plots representing the accuracy of NIPT test for trisomy chromosome 5 using an extended method. The extended methodology is to consider as true positive cases, in addition to those confirmed by genetic testing, intrauterine fetal death and cases terminated by ultrasound.


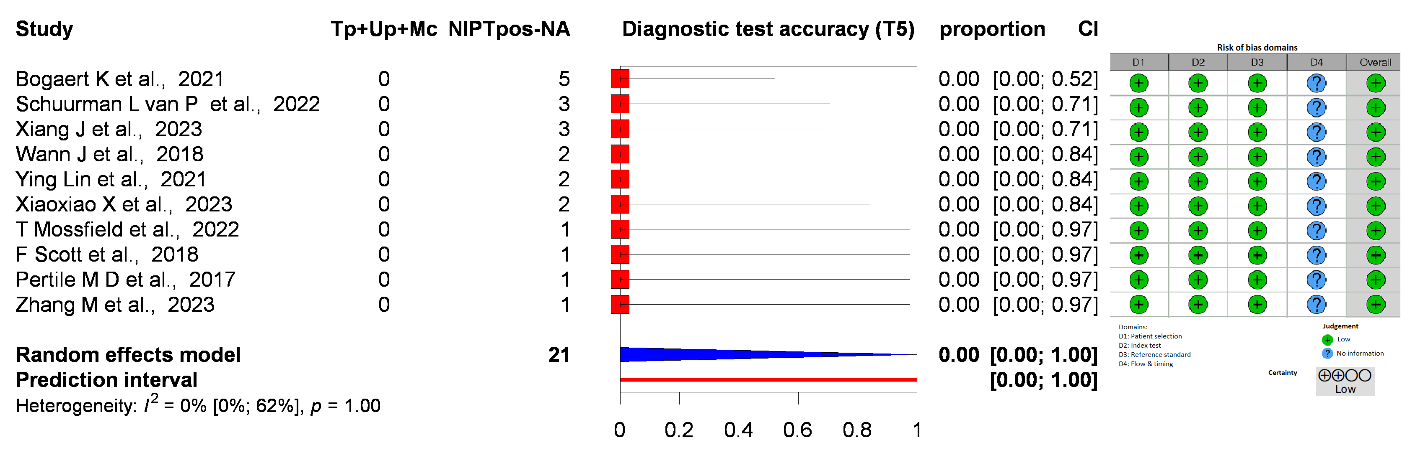


**Figure S6a.** Forest plots representing the accuracy of NIPT test for trisomy chromosome 6 using a confirmed method. The confirmed methodology is to consider only what has been confirmed by genetic testing as a true positive case.


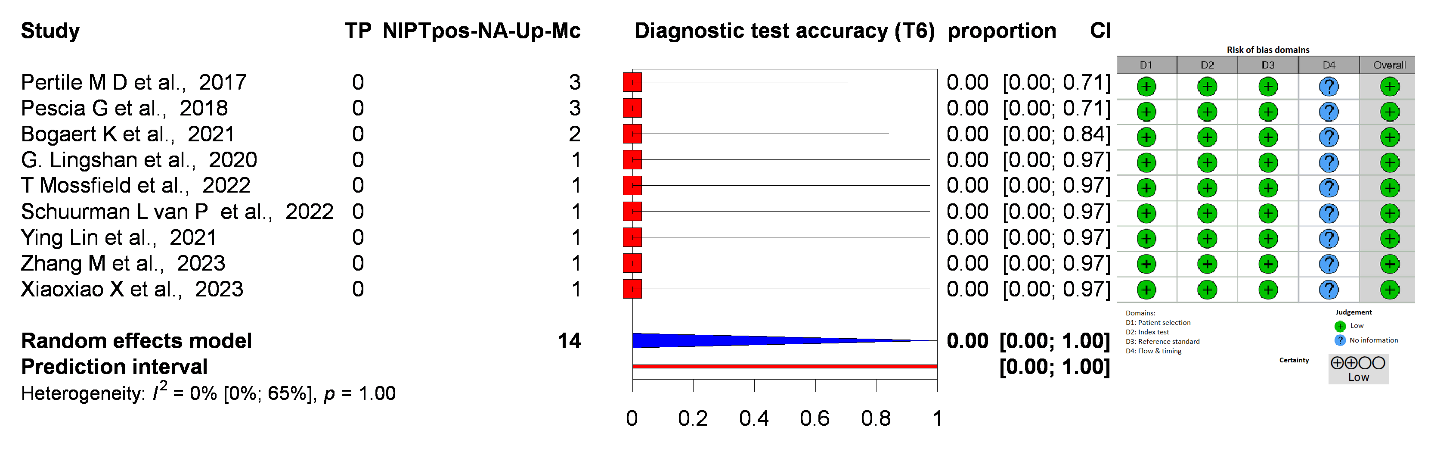


**Figure S6b.** Forest plots representing the accuracy of NIPT test for trisomy chromosome 6 using an extended method. The extended methodology is to consider as true positive cases, in addition to those confirmed by genetic testing, intrauterine fetal death and cases terminated by ultrasound.


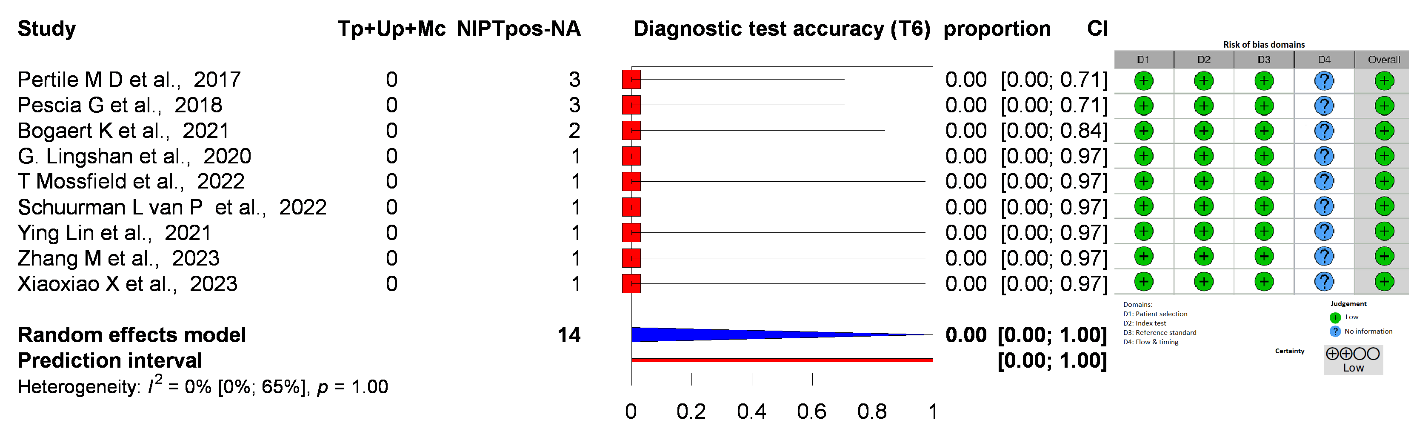


**Figure S7a.** Forest plots representing the accuracy of NIPT test for trisomy chromosome 7 using a confirmed method. The confirmed methodology is to consider only what has been confirmed by genetic testing as a true positive case.


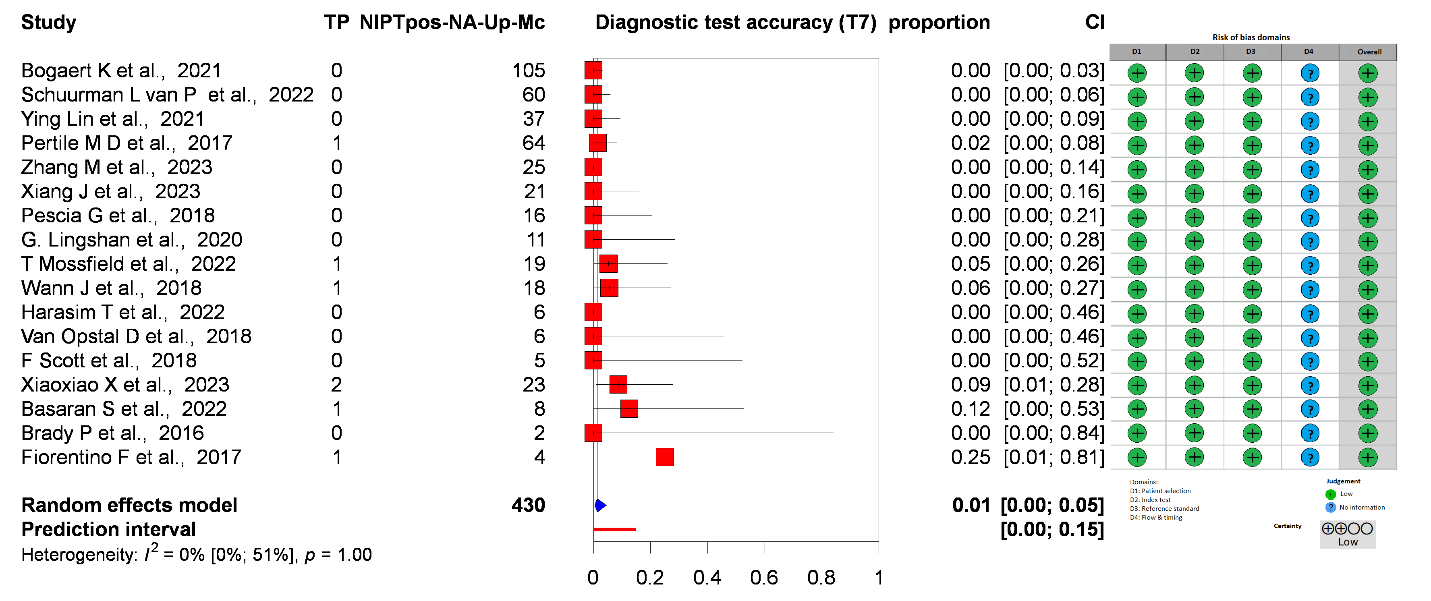


**Figure S7b.** Forest plots representing the accuracy of NIPT test for trisomy chromosome 7 using an extended method. The extended methodology is to consider as true positive cases, in addition to those confirmed by genetic testing, intrauterine fetal death and cases terminated by ultrasound.


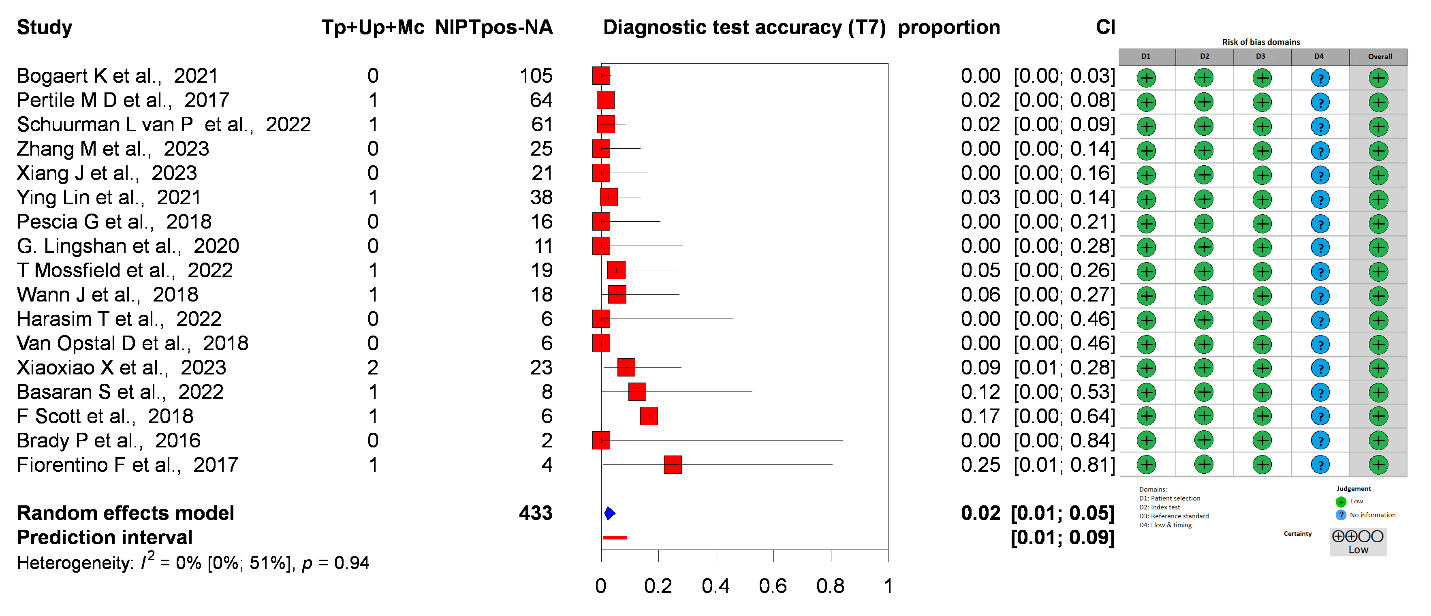


**Figure S8a.** Forest plots representing the accuracy of NIPT test for trisomy chromosome 8 using a confirmed method. The confirmed methodology is to consider only what has been confirmed by genetic testing as a true positive case.


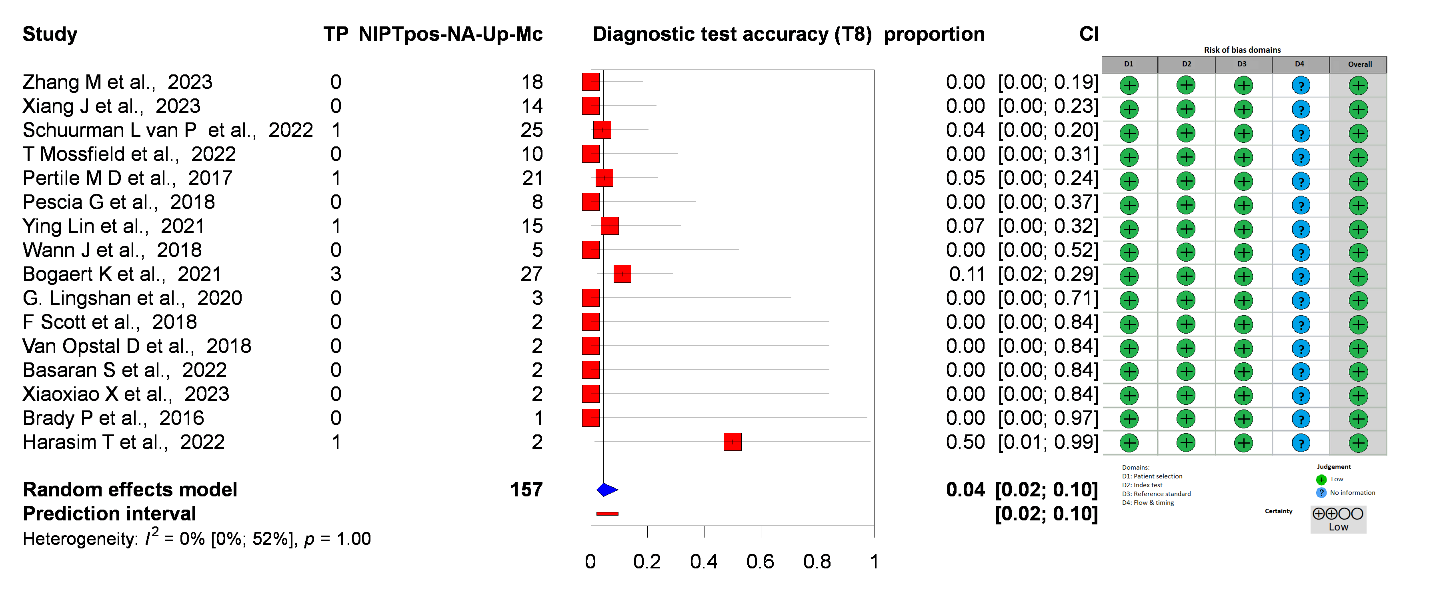


**Figure S8b.** Forest plots representing the accuracy of NIPT test for trisomy chromosome 8 using an extended method. The extended methodology is to consider as true positive cases, in addition to those confirmed by genetic testing, intrauterine fetal death and cases terminated by ultrasound.


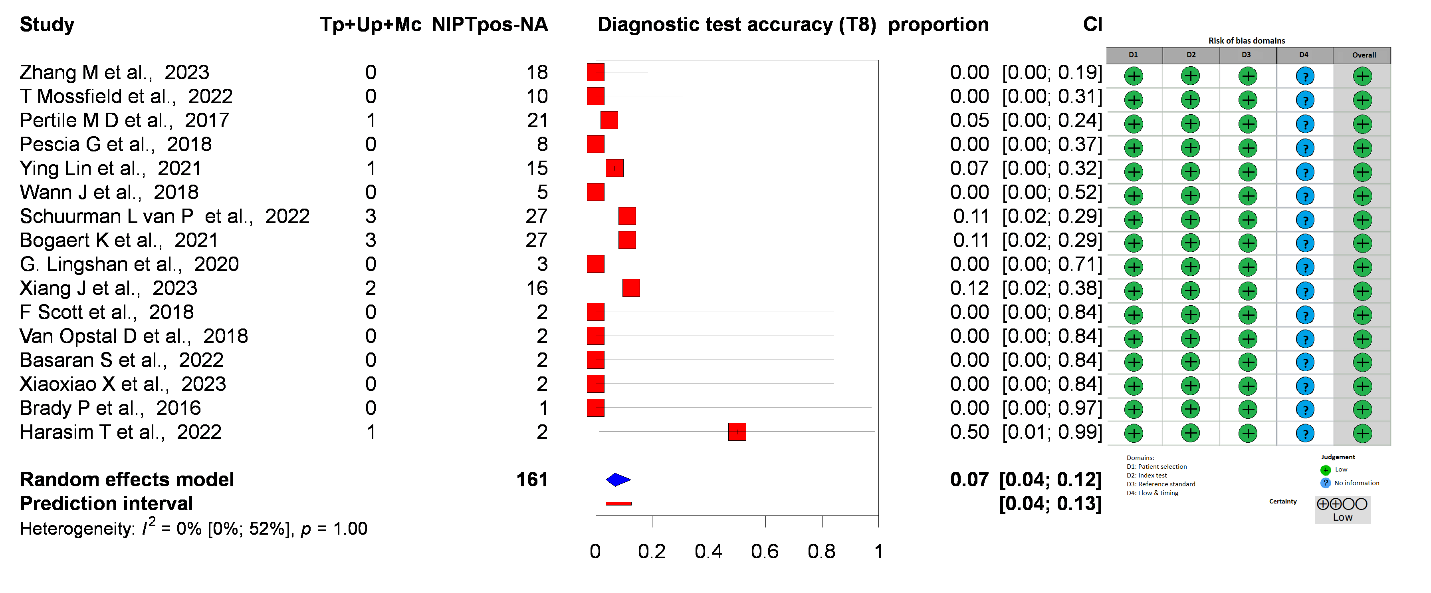


**Figure S9a.** Forest plots representing the accuracy of NIPT test for trisomy chromosome 9 using a confirmed method. The confirmed methodology is to consider only what has been confirmed by genetic testing as a true positive case.


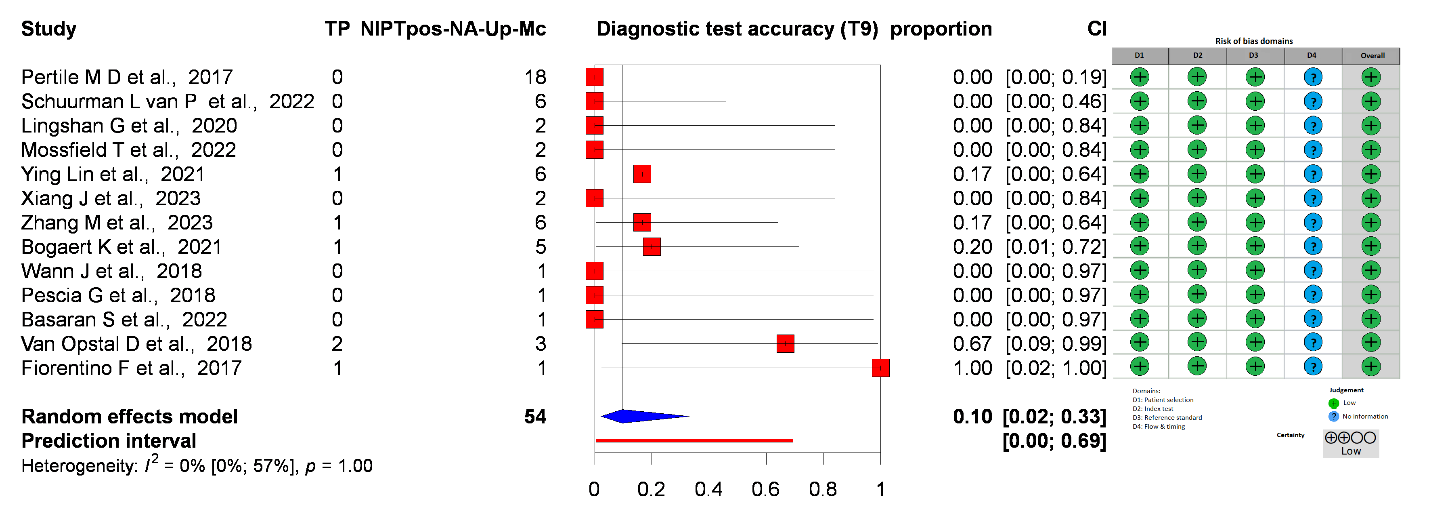


**Figure S9b.** Forest plots representing the accuracy of NIPT test for trisomy chromosome 9 using an extended method. The extended methodology is to consider as true positive cases, in addition to those confirmed by genetic testing, intrauterine fetal death and cases terminated by ultrasound.


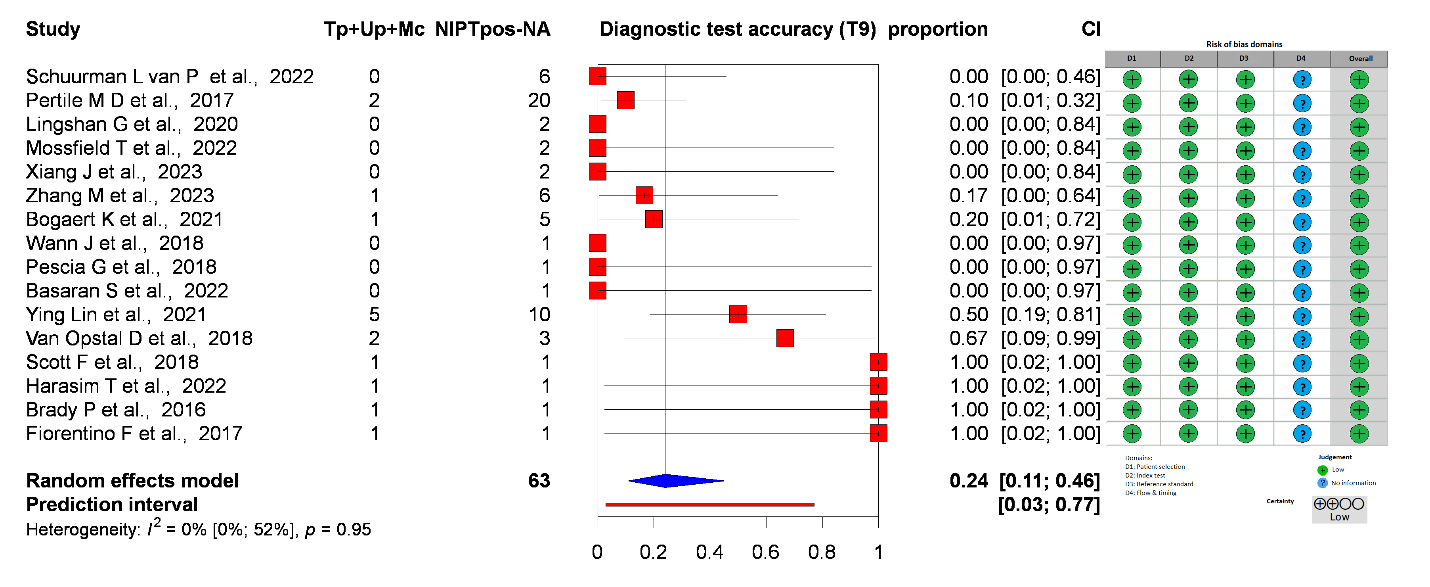


**Figure S10a.** Forest plots representing the accuracy of NIPT test for trisomy chromosome 10 using a confirmed method. The confirmed methodology is to consider only what has been confirmed by genetic testing as a true positive case.


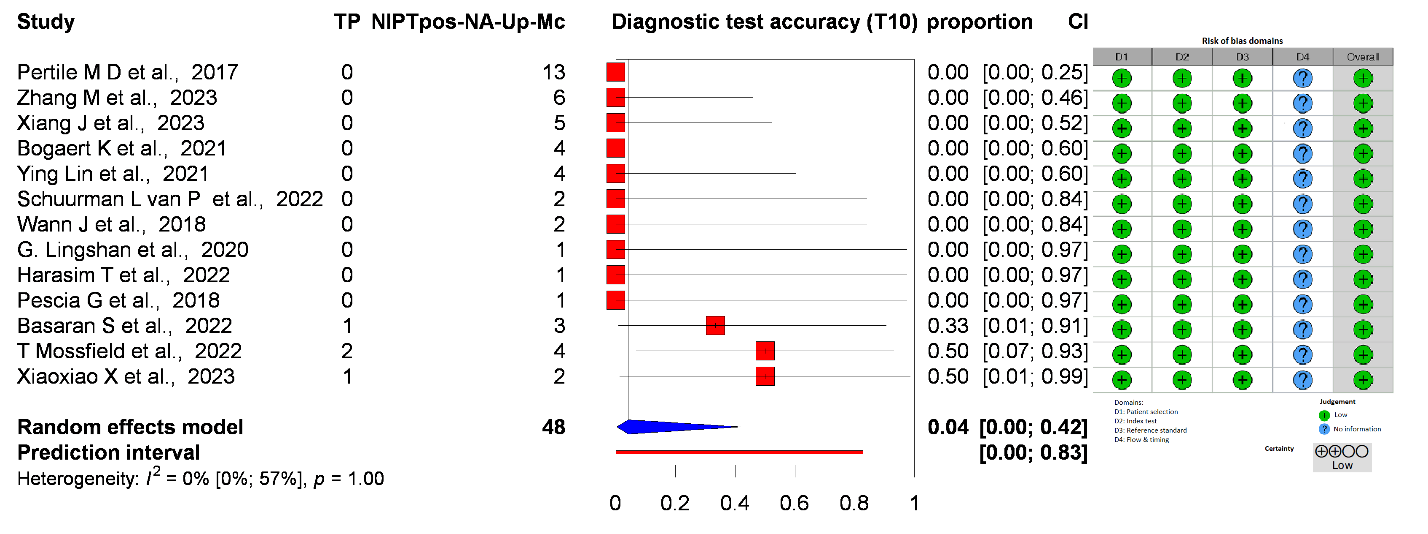


**Figure S10b.** Forest plots representing the accuracy of NIPT test for trisomy chromosome 10 using an extended method. The extended methodology is to consider as true positive cases, in addition to those confirmed by genetic testing, intrauterine fetal death and cases terminated by ultrasound.


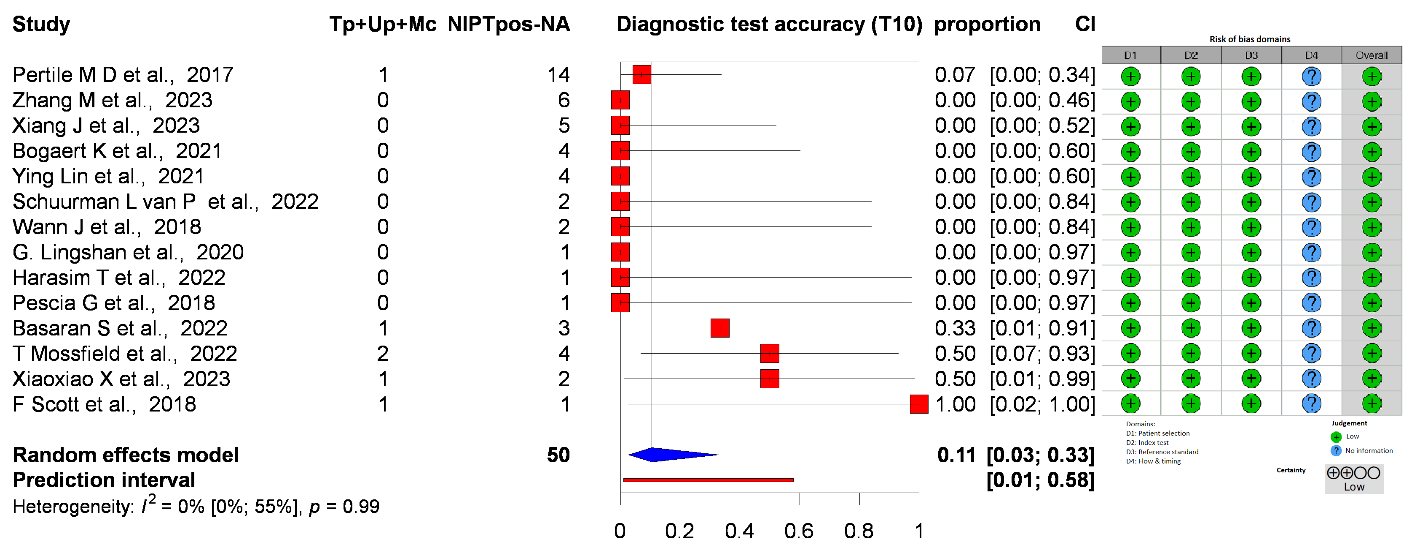


**Figure S11a.** Forest plots representing the accuracy of NIPT test for trisomy chromosome 11 using a confirmed method. The confirmed methodology is to consider only what has been confirmed by genetic testing as a true positive case.


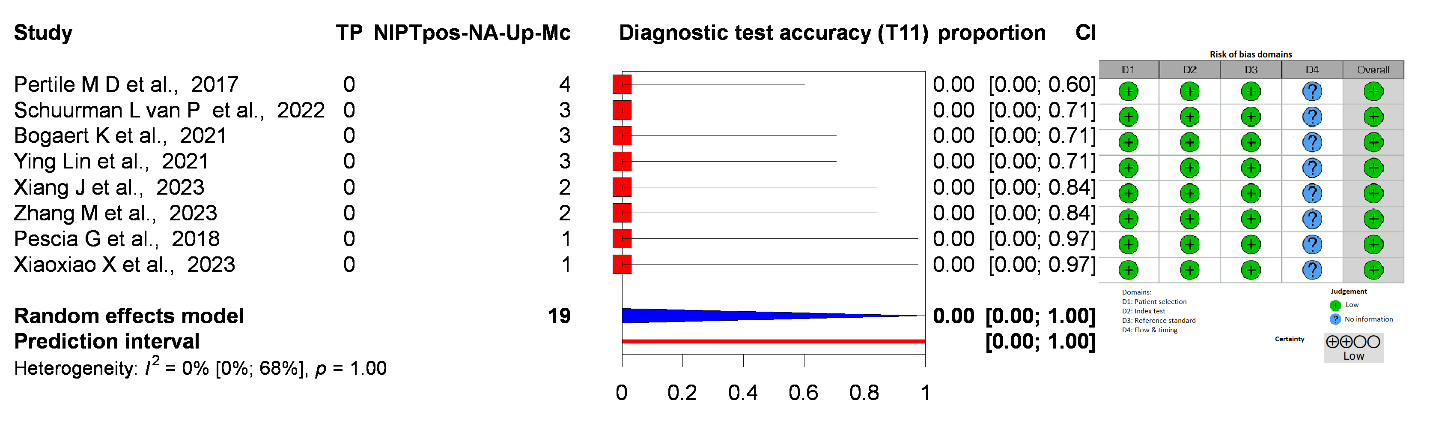


**Figure S11b.** Forest plots representing the accuracy of NIPT test for trisomy chromosome 11 using an extended method. The extended methodology is to consider as true positive cases, in addition to those confirmed by genetic testing, intrauterine fetal death and cases terminated by ultrasound.


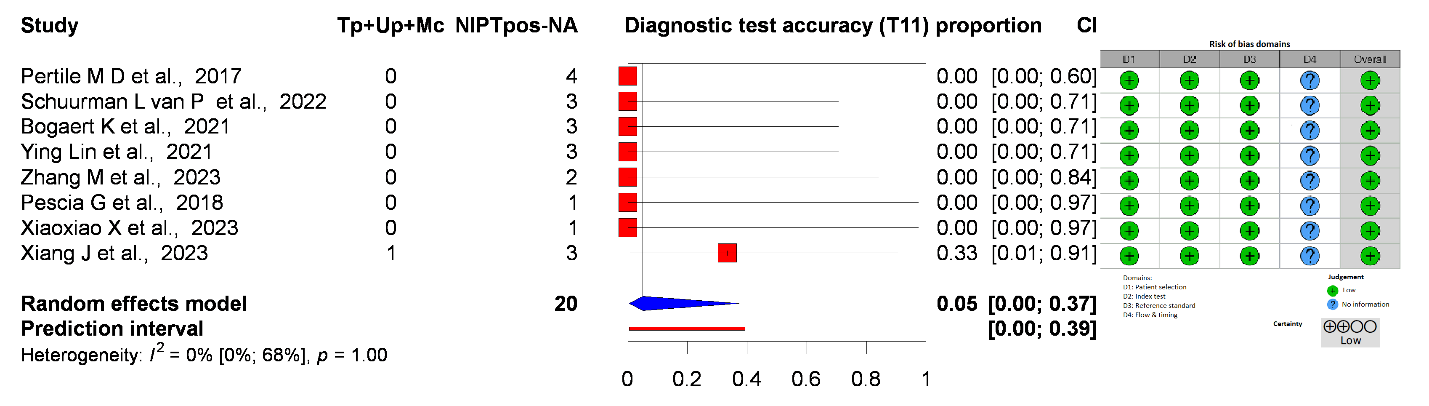


**Figure S12a.** Forest plots representing the accuracy of NIPT test for trisomy chromosome 12 using a confirmed method. The confirmed methodology is to consider only what has been confirmed by genetic testing as a true positive case.


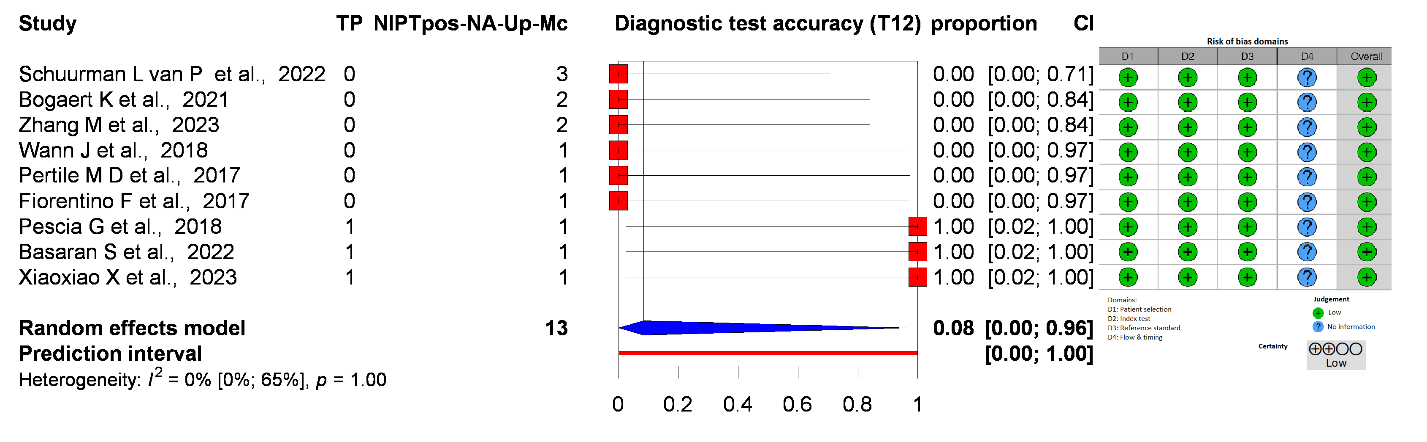


**Figure S12b**. Forest plots representing the accuracy of NIPT test for trisomy chromosome 12 using an extended method. The extended methodology is to consider as true positive cases, in addition to those confirmed by genetic testing, intrauterine fetal death and cases terminated by ultrasound.


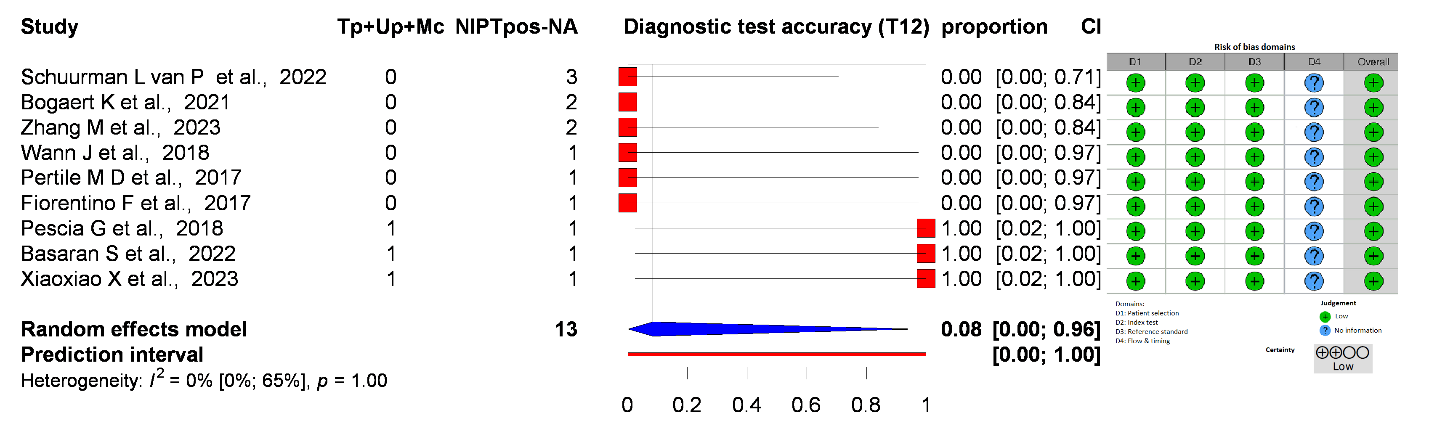


**Figure S13a.** Forest plots representing the accuracy of NIPT test for trisomy chromosome 14 using a confirmed method. The confirmed methodology is to consider only what has been confirmed by genetic testing as a true positive case.


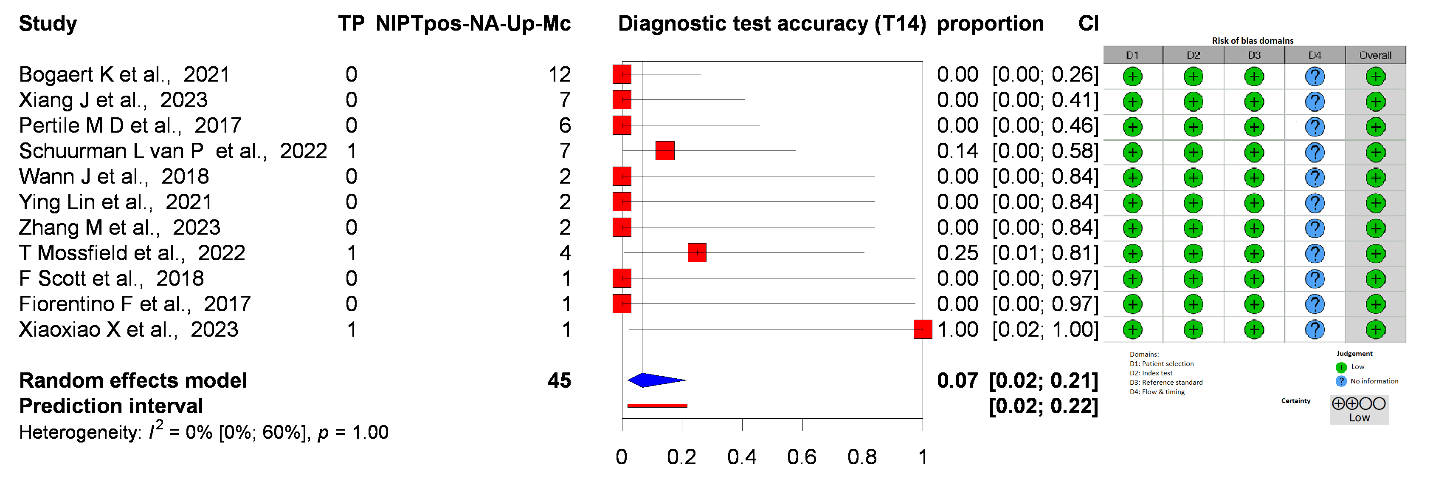


**Figure S13b.** Forest plots representing the accuracy of NIPT test for trisomy chromosome 14 using an extended method. The extended methodology is to consider as true positive cases, in addition to those confirmed by genetic testing, intrauterine fetal death and cases terminated by ultrasound.


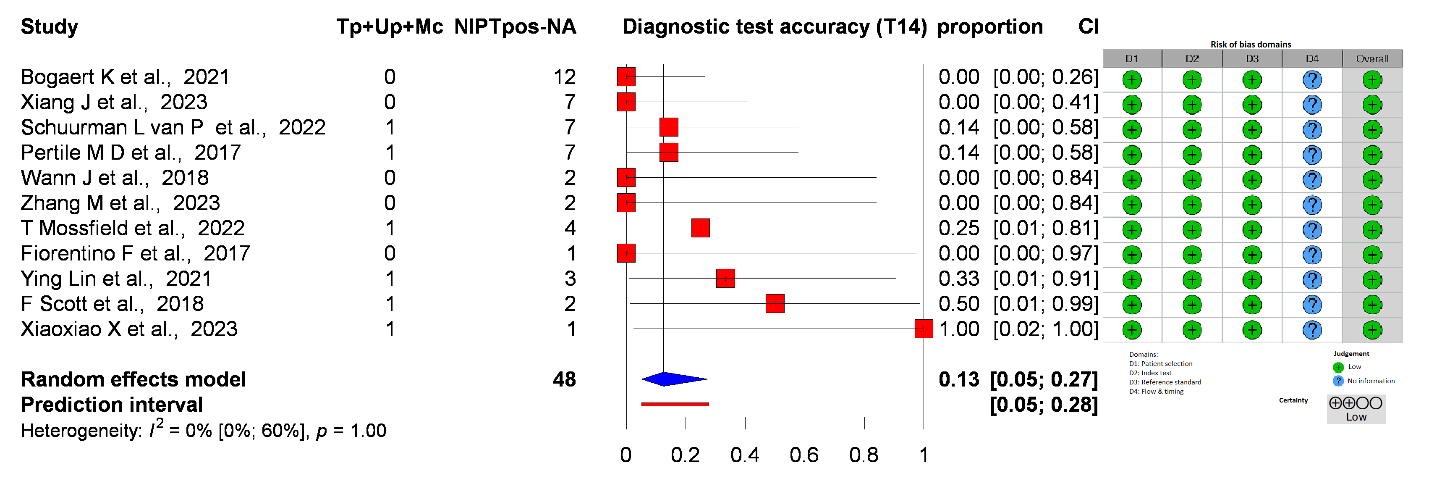


**Figure S14a.** Forest plots representing the accuracy of NIPT test for trisomy chromosome 15 using a confirmed method. The confirmed methodology is to consider only what has been confirmed by genetic testing as a true positive case.


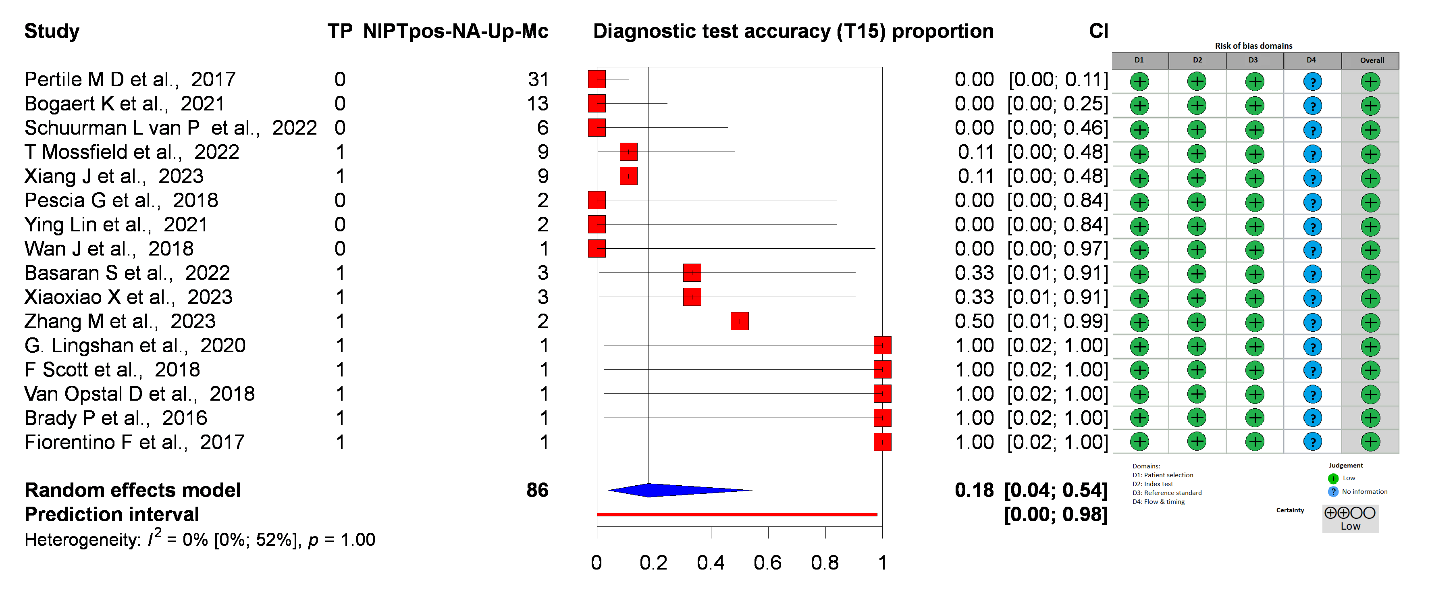


**Figure S14b.** Forest plots representing the accuracy of NIPT test for trisomy chromosome 15 using an extended method. The extended methodology is to consider as true positive cases, in addition to those confirmed by genetic testing, intrauterine fetal death and cases terminated by ultrasound.


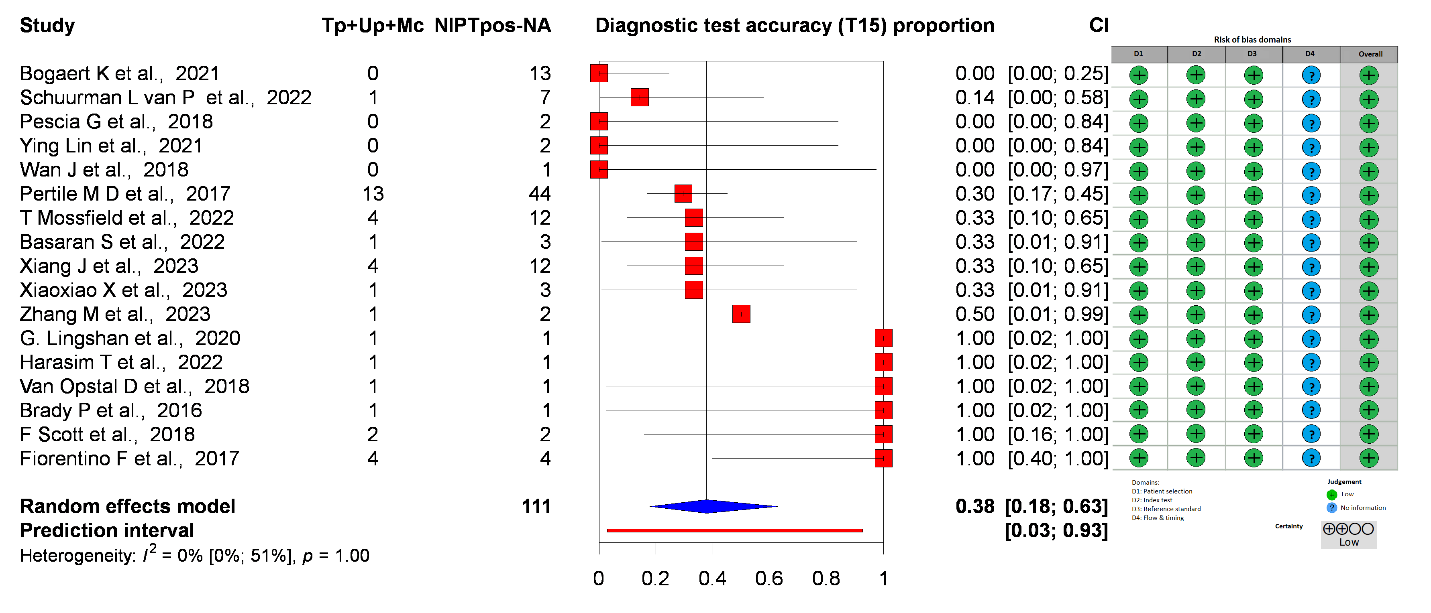


**Figure S15a.** Forest plots representing the accuracy of NIPT test for trisomy chromosome 16 using a confirmed method. The confirmed methodology is to consider only what has been confirmed by genetic testing as a true positive case.


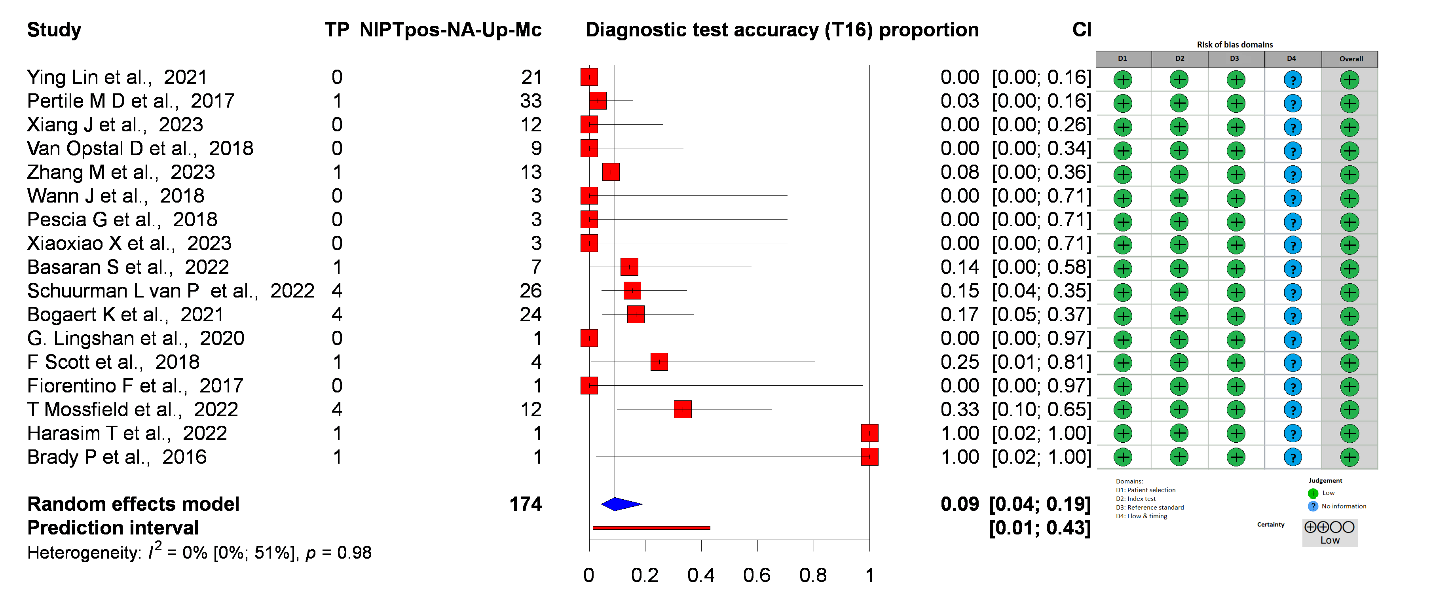


**Figure S15b.** Forest plots representing the accuracy of NIPT test for trisomy chromosome 16 using an extended method. The extended methodology is to consider as true positive cases, in addition to those confirmed by genetic testing, intrauterine fetal death and cases terminated by ultrasound.


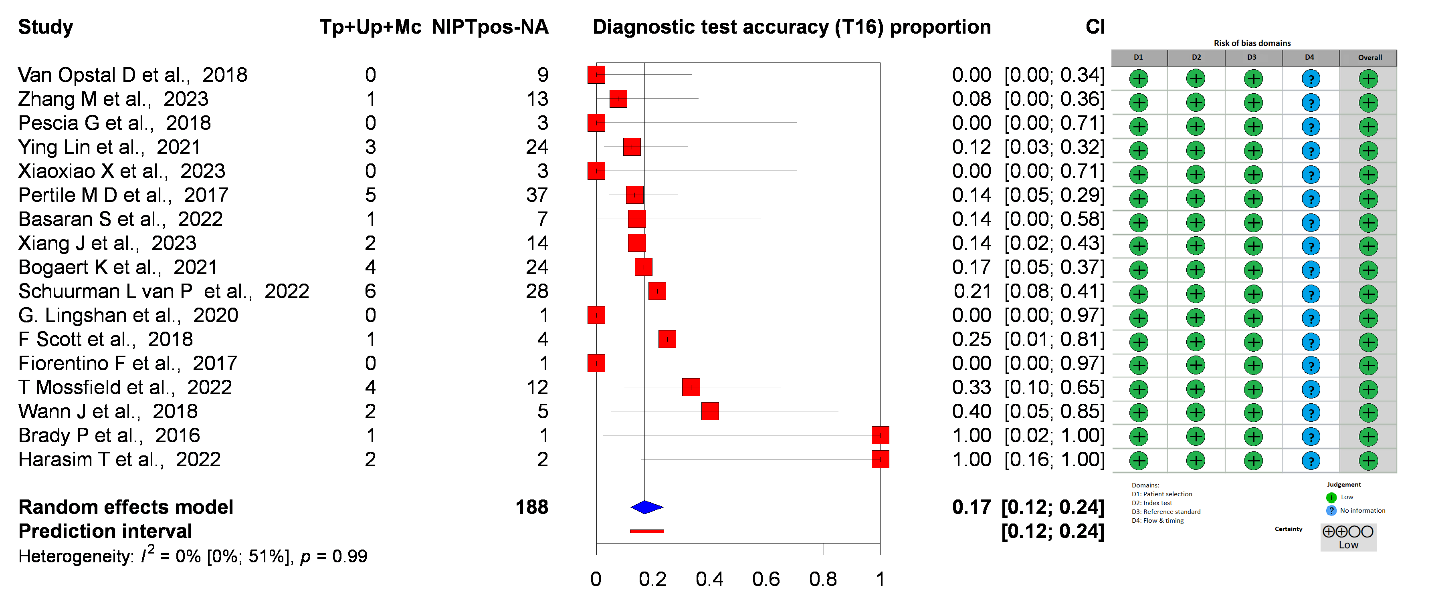


**Figure S16a.** Forest plots representing the accuracy of NIPT test for trisomy chromosome 17 using a confirmed method. The confirmed methodology is to consider only what has been confirmed by genetic testing as a true positive case.


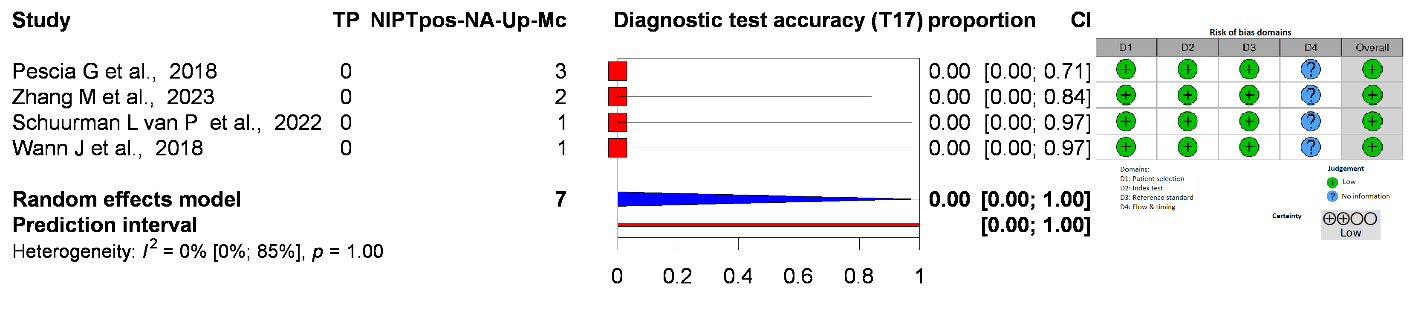


**Figure S16b.** Forest plots representing the accuracy of NIPT test for trisomy chromosome 17 using an extended method. The extended methodology is to consider as true positive cases, in addition to those confirmed by genetic testing, intrauterine fetal death and cases terminated by ultrasound.


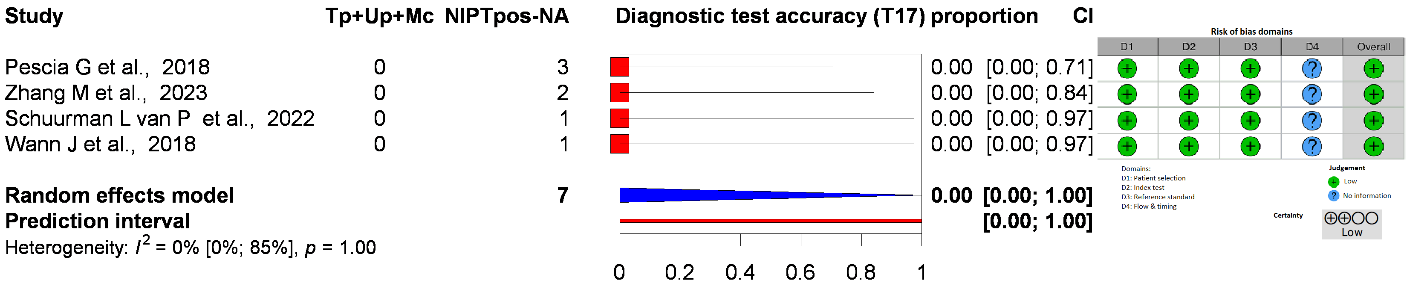


**Figure S17a.** Forest plots representing the accuracy of NIPT test for trisomy chromosome 19 using a confirmed method. The confirmed methodology is to consider only what has been confirmed by genetic testing as a true positive case.


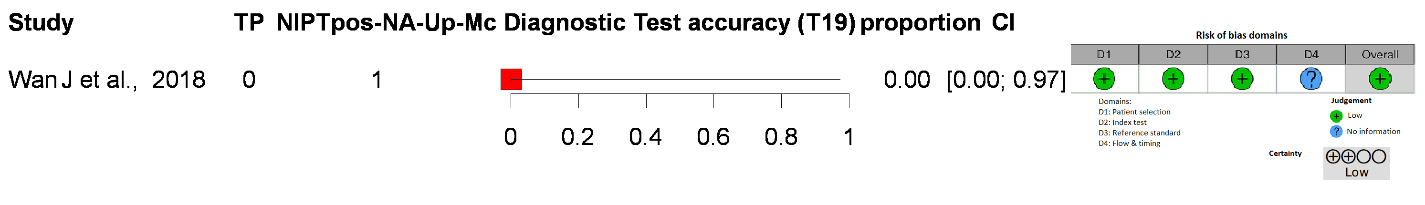


**Figure S17b.** Forest plots representing the accuracy of NIPT test for trisomy chromosome 19 using an extended method. The extended methodology is to consider as true positive cases, in addition to those confirmed by genetic testing, intrauterine fetal death and cases terminated by ultrasound.

**
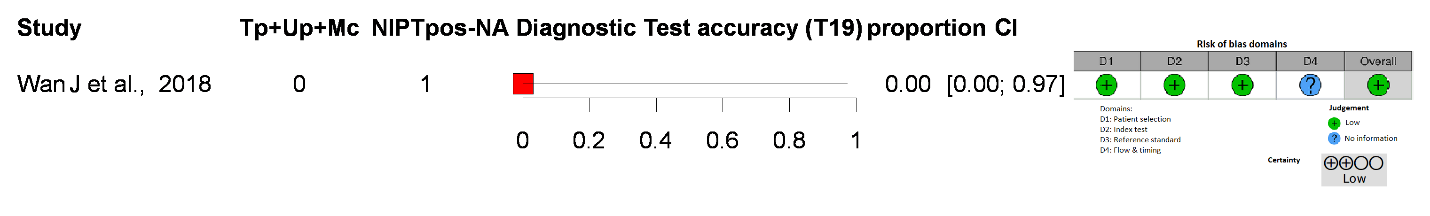
**

**Figure S18a.** Forest plots representing the accuracy of NIPT test for trisomy chromosome 20 using a confirmed method. The confirmed methodology is to consider only what has been confirmed by genetic testing as a true positive case.


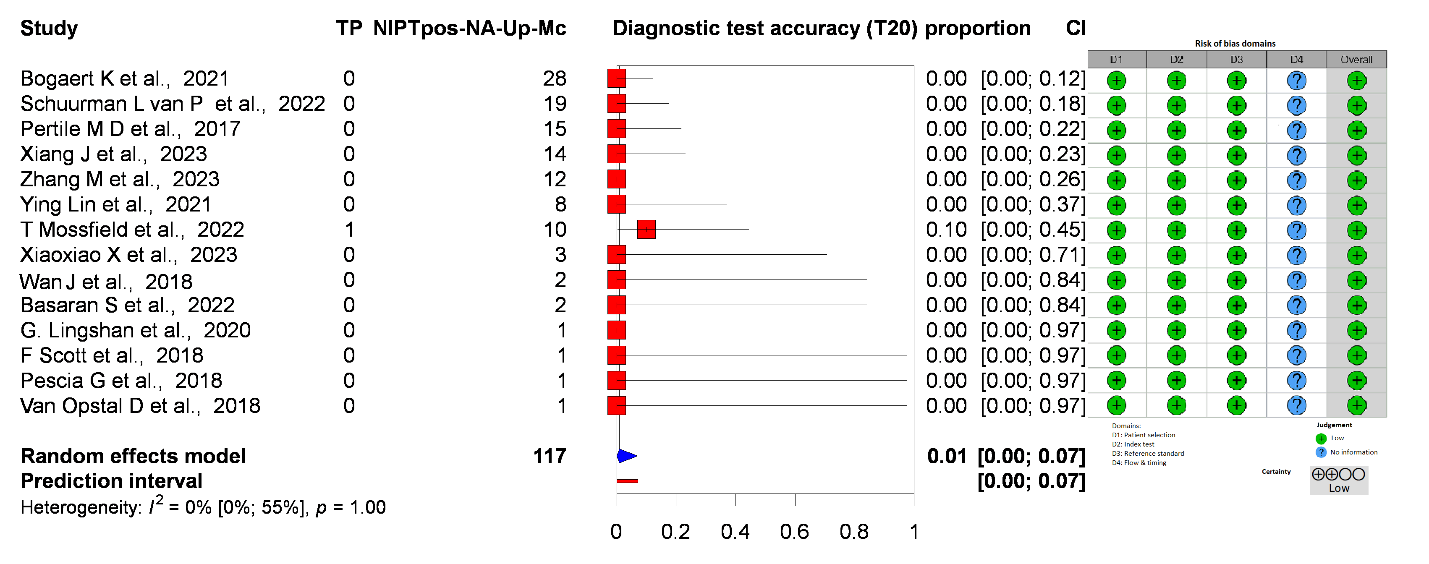


**Figure S18b.** Forest plots representing the accuracy of NIPT test for trisomy chromosome 20 using an extended method. The extended methodology is to consider as true positive cases, in addition to those confirmed by genetic testing, intrauterine fetal death and cases terminated by ultrasound.


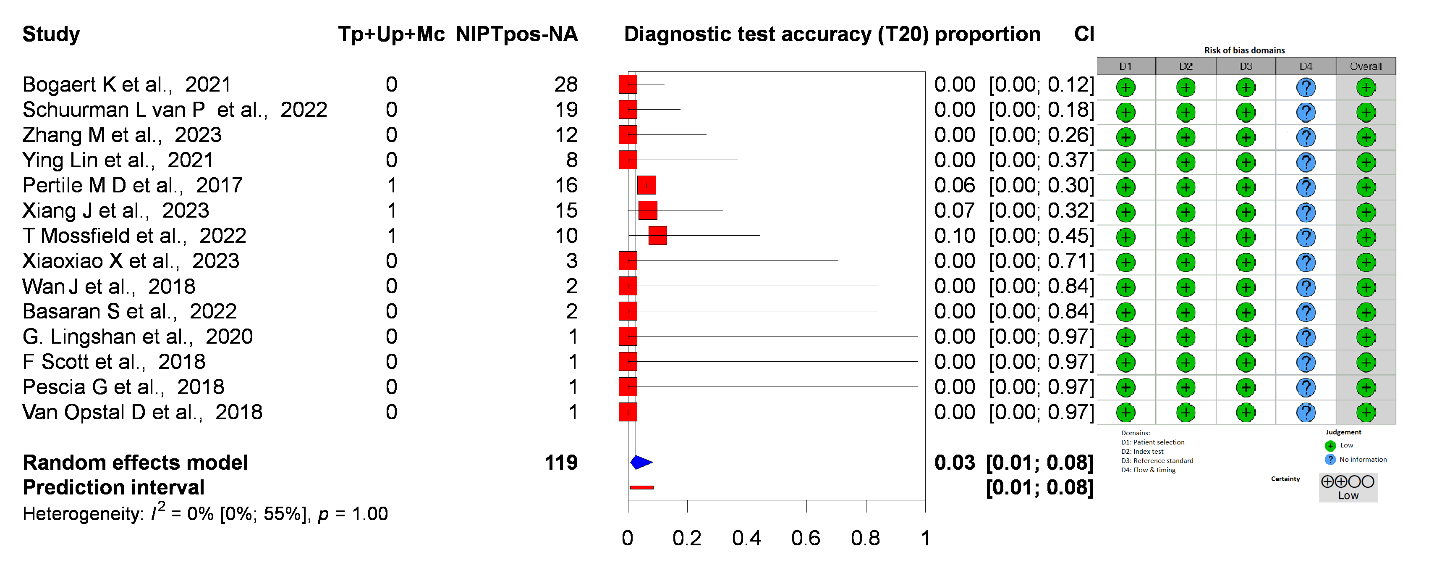


**Figure S19a.** Forest plots representing the accuracy of NIPT test for trisomy chromosome 22 using a confirmed method. The confirmed methodology is to consider only what has been confirmed by genetic testing as a true positive case.


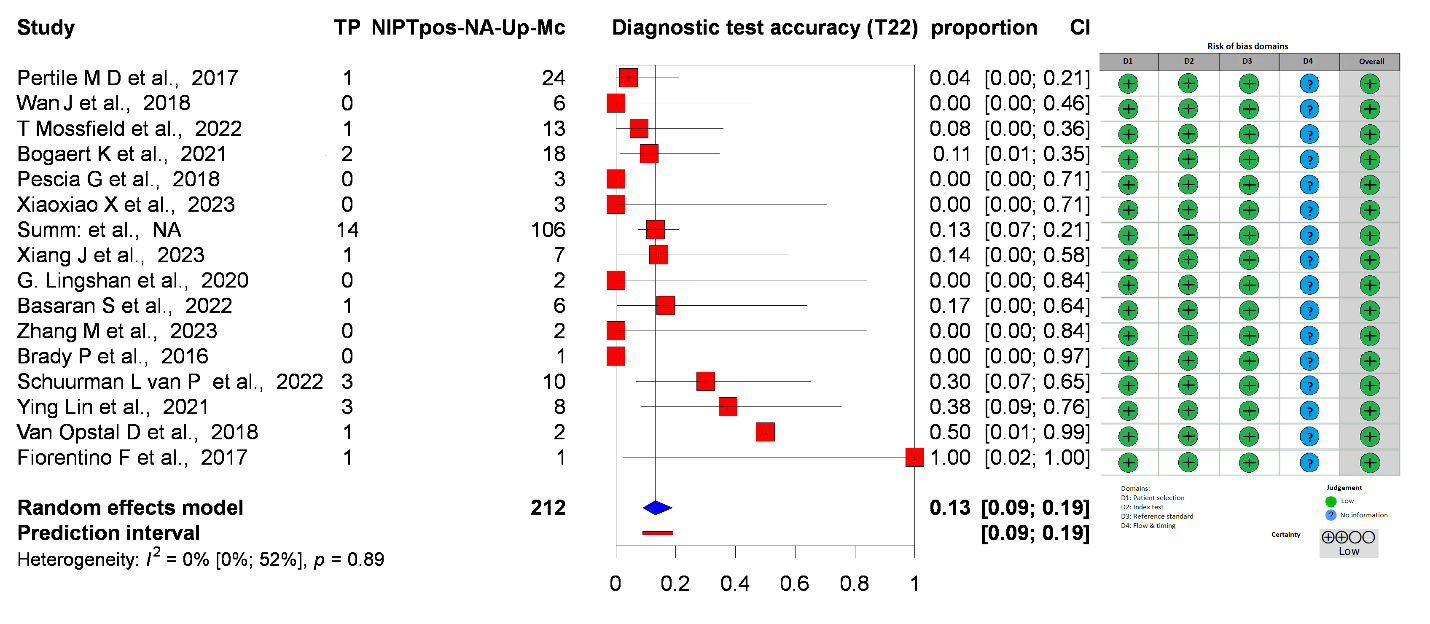


**Figure S19b.** Forest plots representing the accuracy of NIPT test for trisomy chromosome 22 using an extended method. The extended methodology is to consider as true positive cases, in addition to those confirmed by genetic testing, intrauterine fetal death and cases terminated by ultrasound.


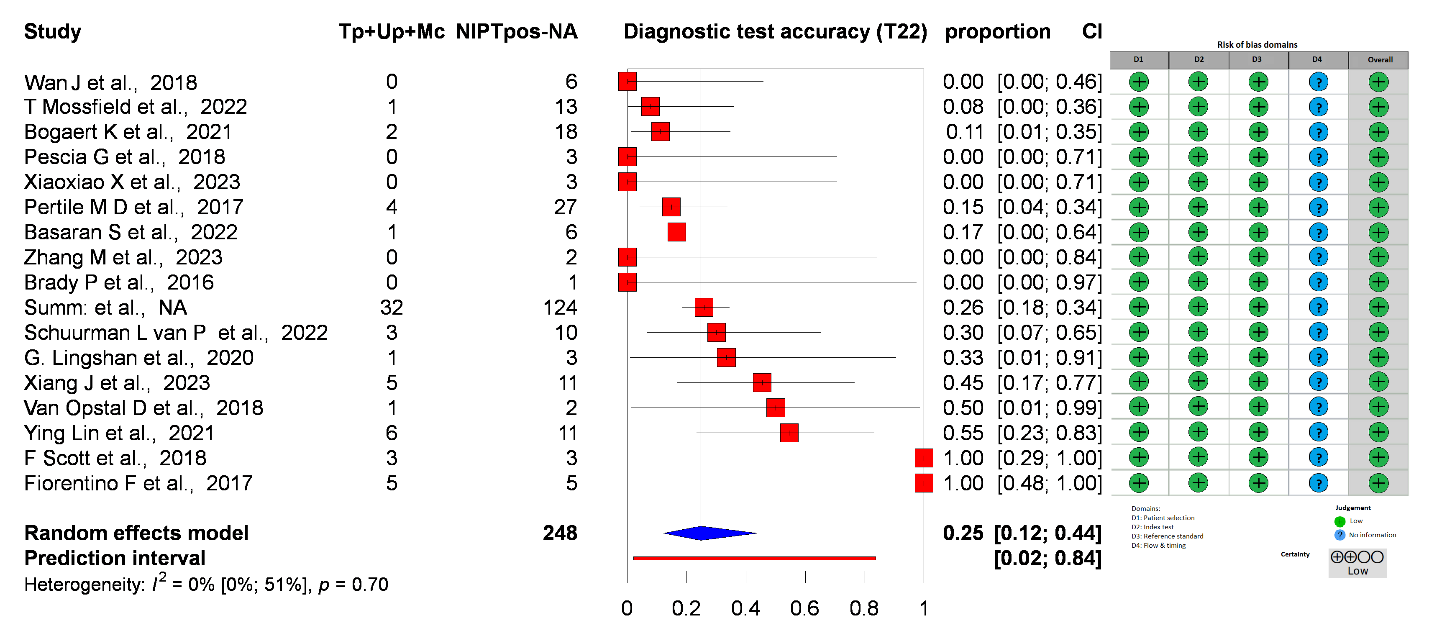


**Figure S20.** Forest plots representing the frequency of NIPT test for trisomy chromosome 1.


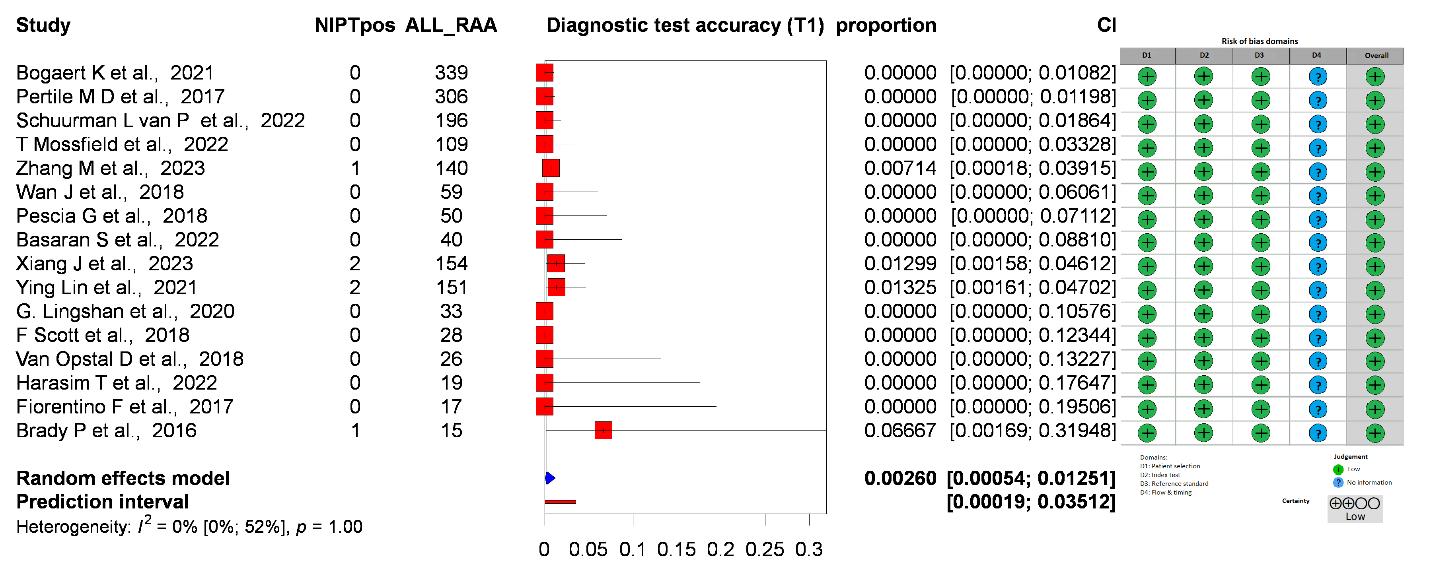


**Figure S21.** Forest plots representing the frequency of NIPT test for trisomy chromosome 2.


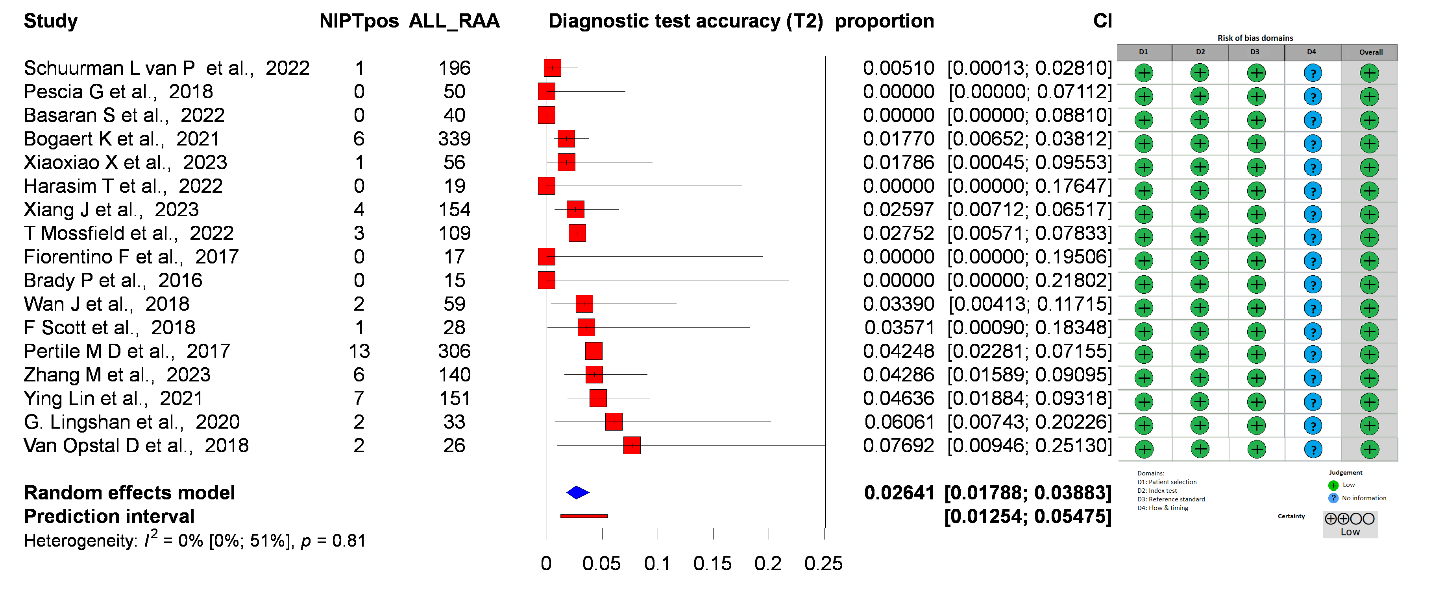


**Figure S22.** Forest plots representing the frequency of NIPT test for trisomy chromosome 3.


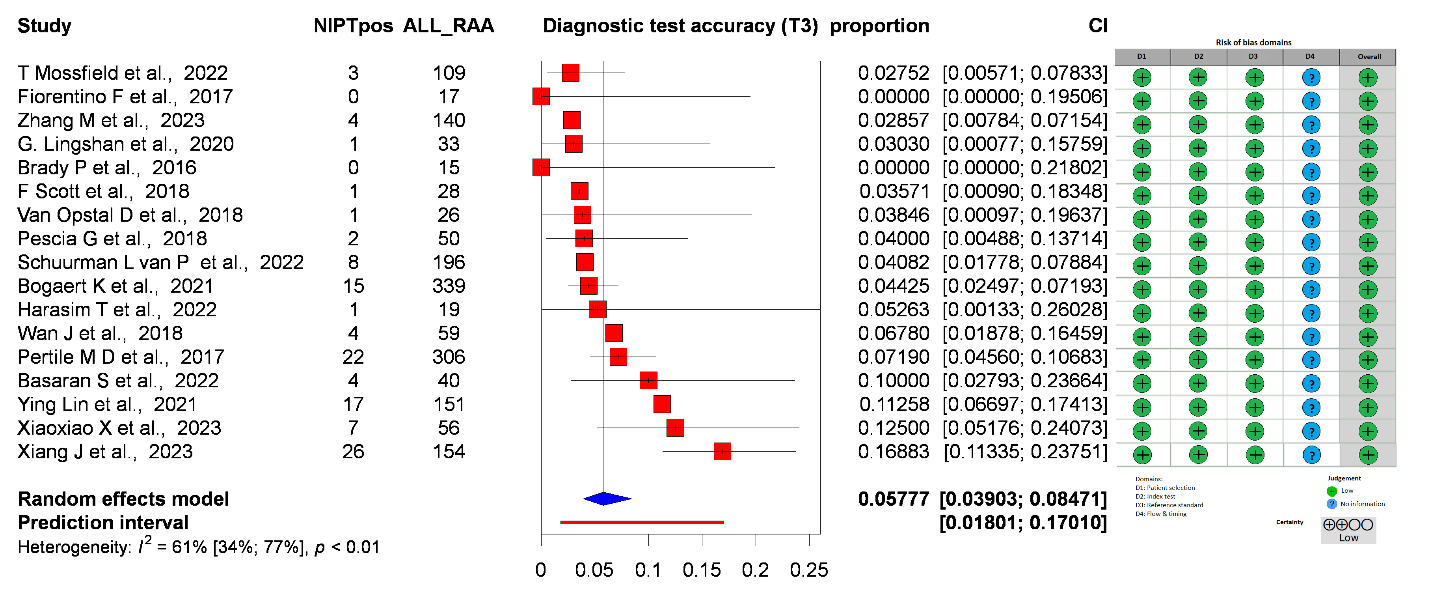


**Figure S23.** Forest plots representing the frequency of NIPT test for trisomy chromosome 4.


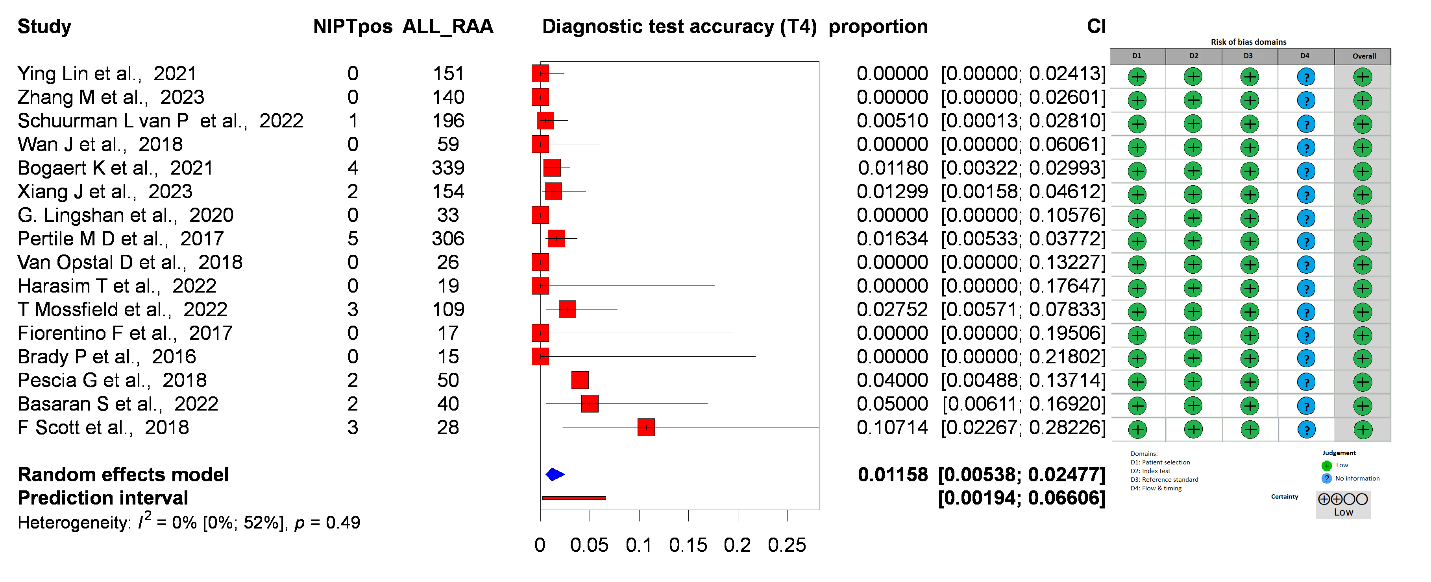


**Figure S24.** Forest plots representing the frequency of NIPT test for trisomy chromosome 5.


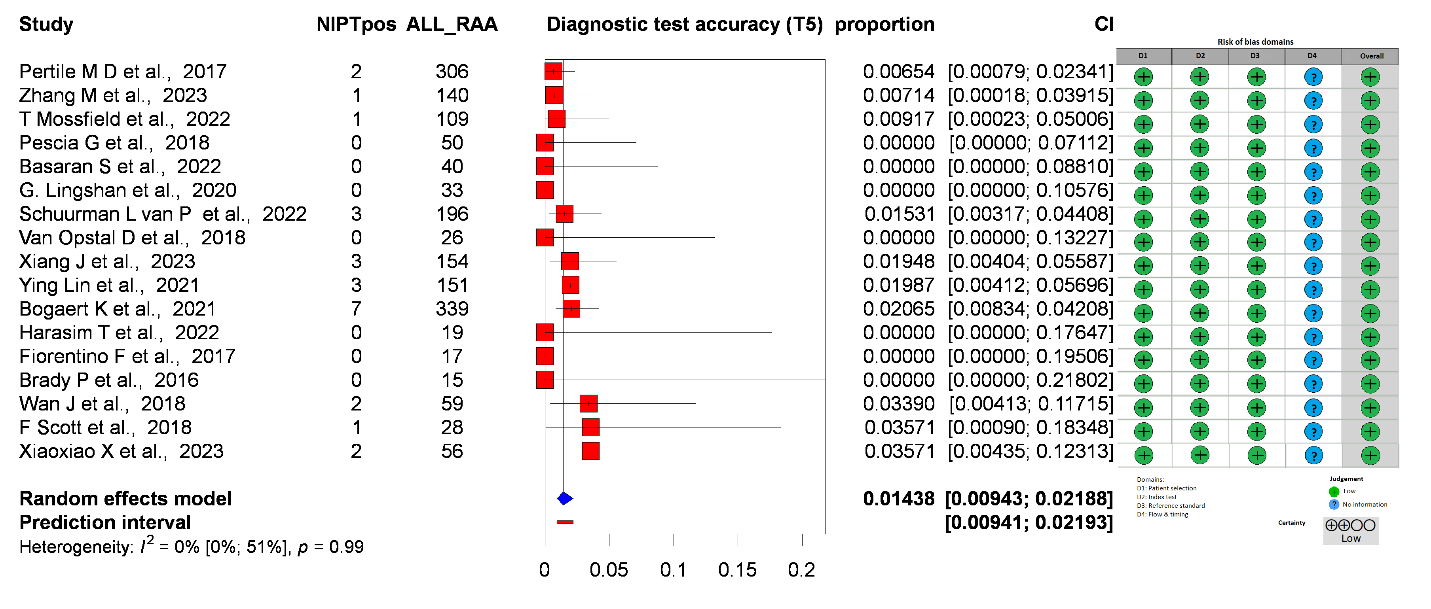


**Figure S25.** Forest plots representing the frequency of NIPT test for trisomy chromosome 6.


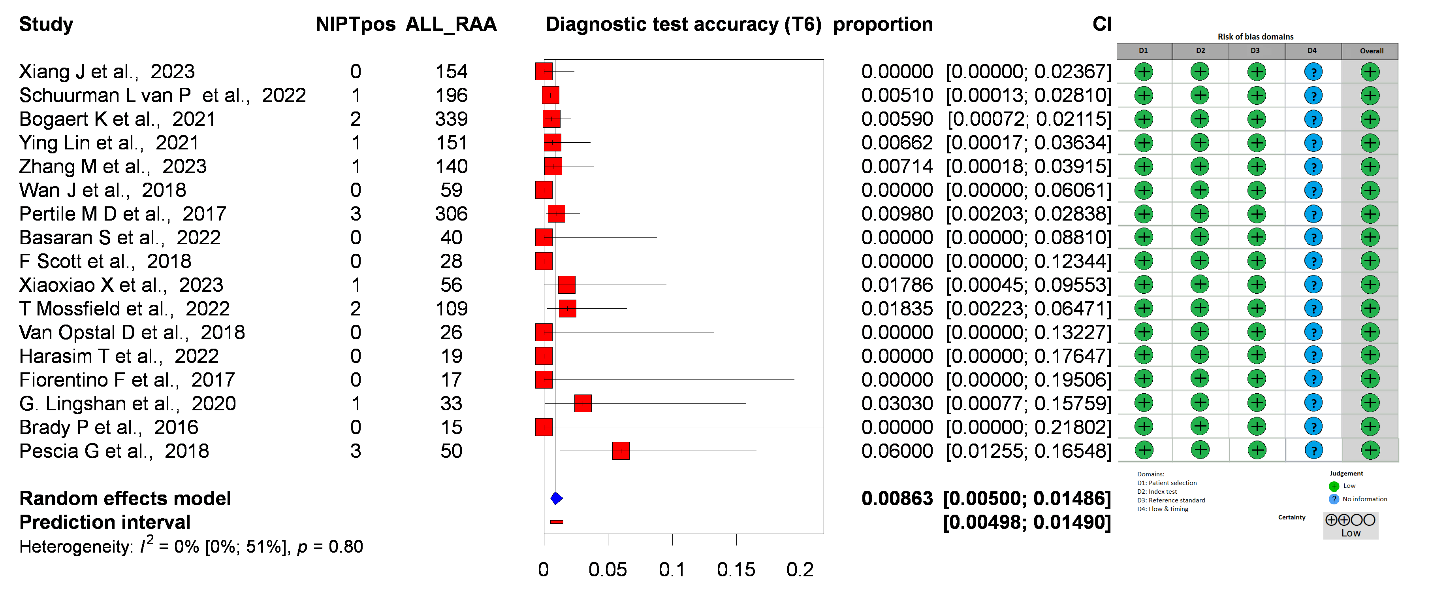


**Figure S26.** Forest plots representing the frequency of NIPT test for trisomy chromosome 7.


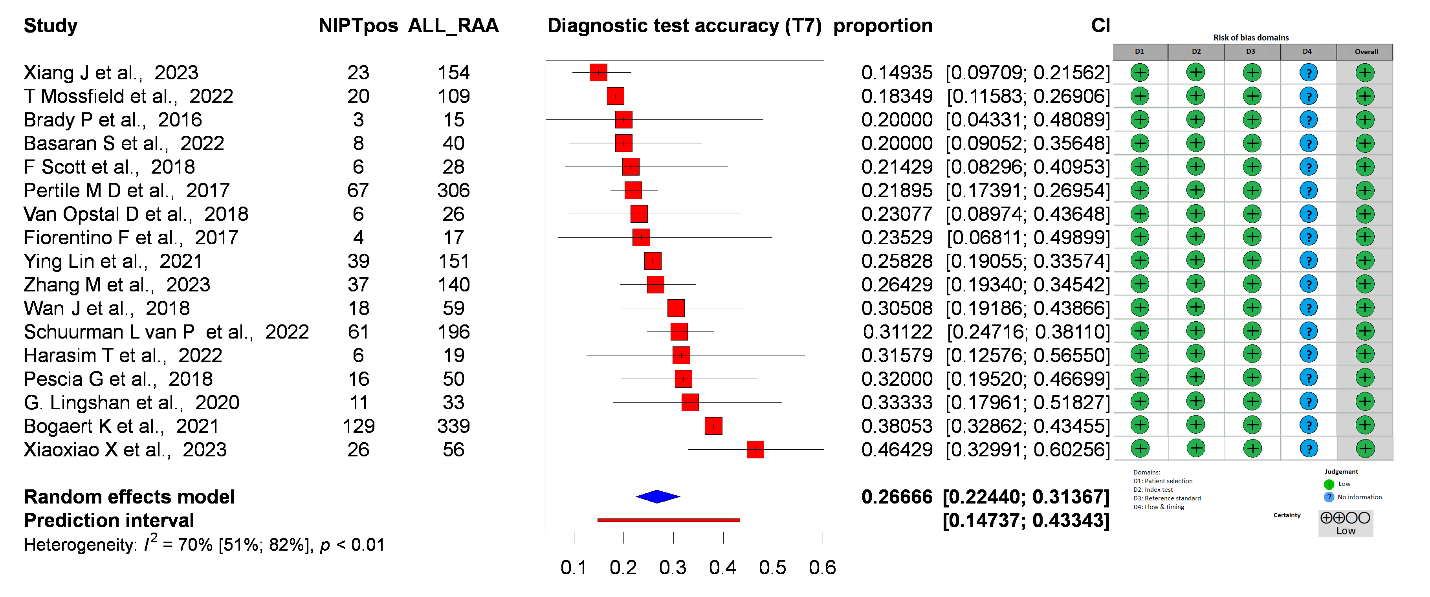


**Figure S27.** Forest plots representing the frequency of NIPT test for trisomy chromosome 8.


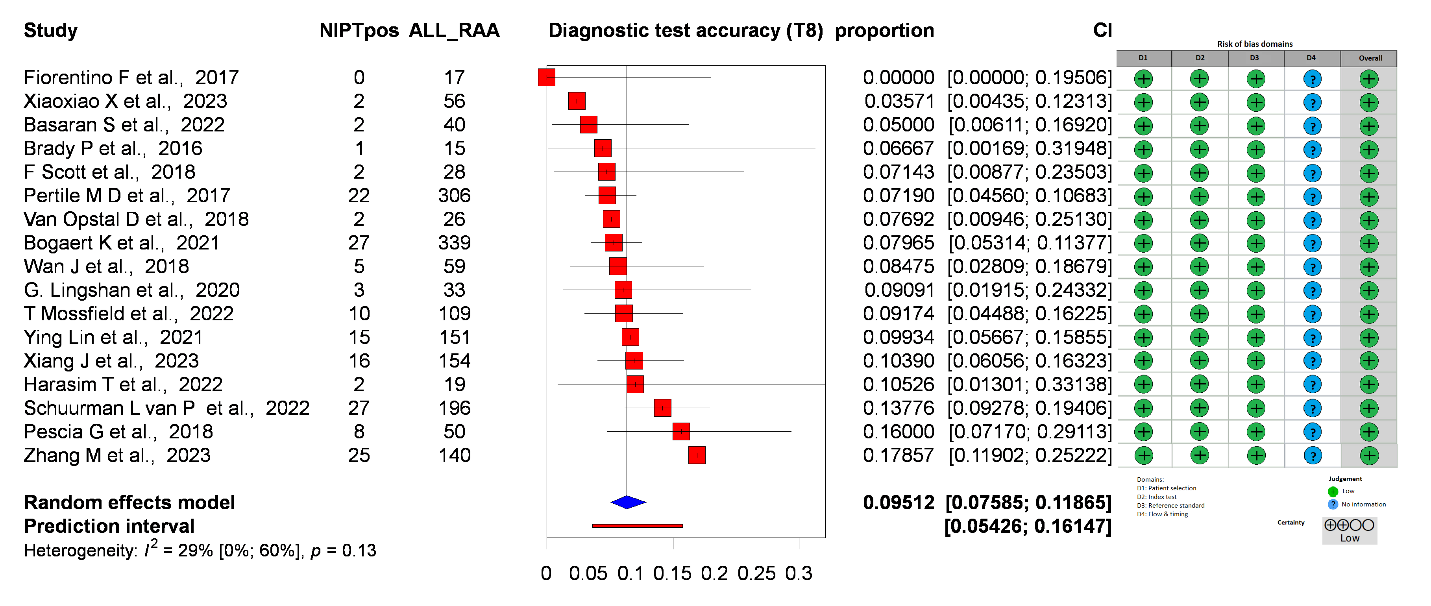


**Figure S28.** Forest plots representing the frequency of NIPT test for trisomy chromosome 9.


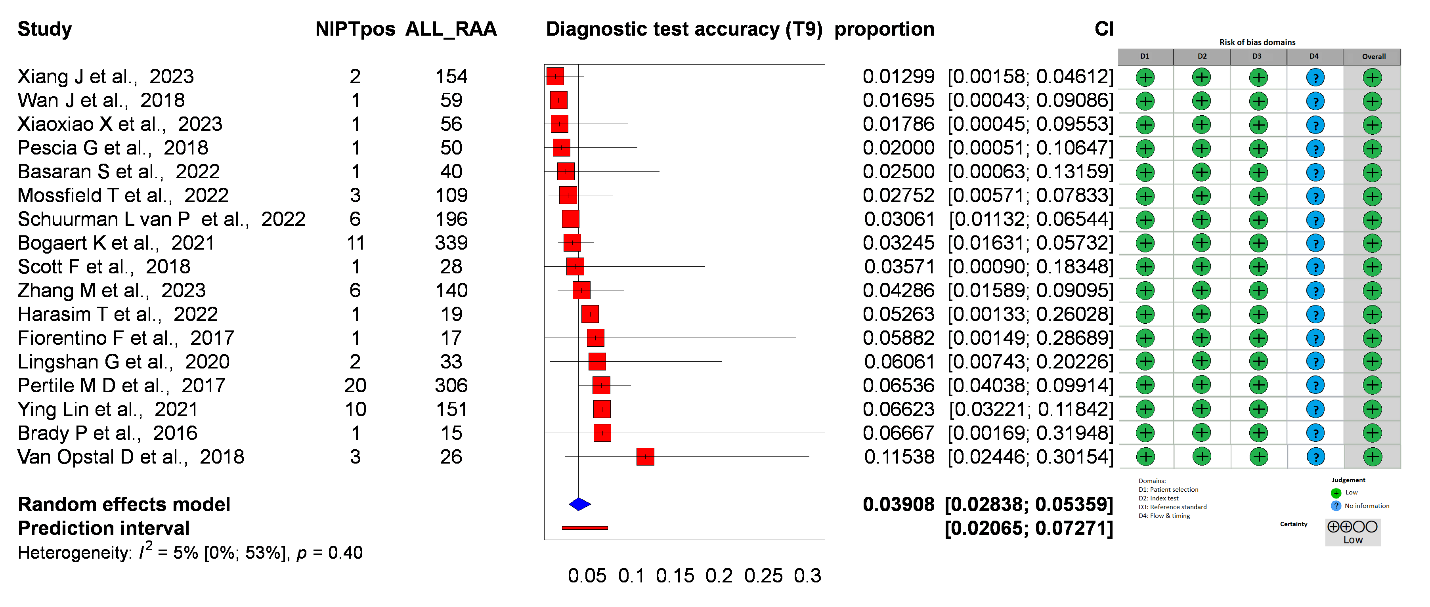


**Figure S29.** Forest plots representing the frequency of NIPT test for trisomy chromosome 10.


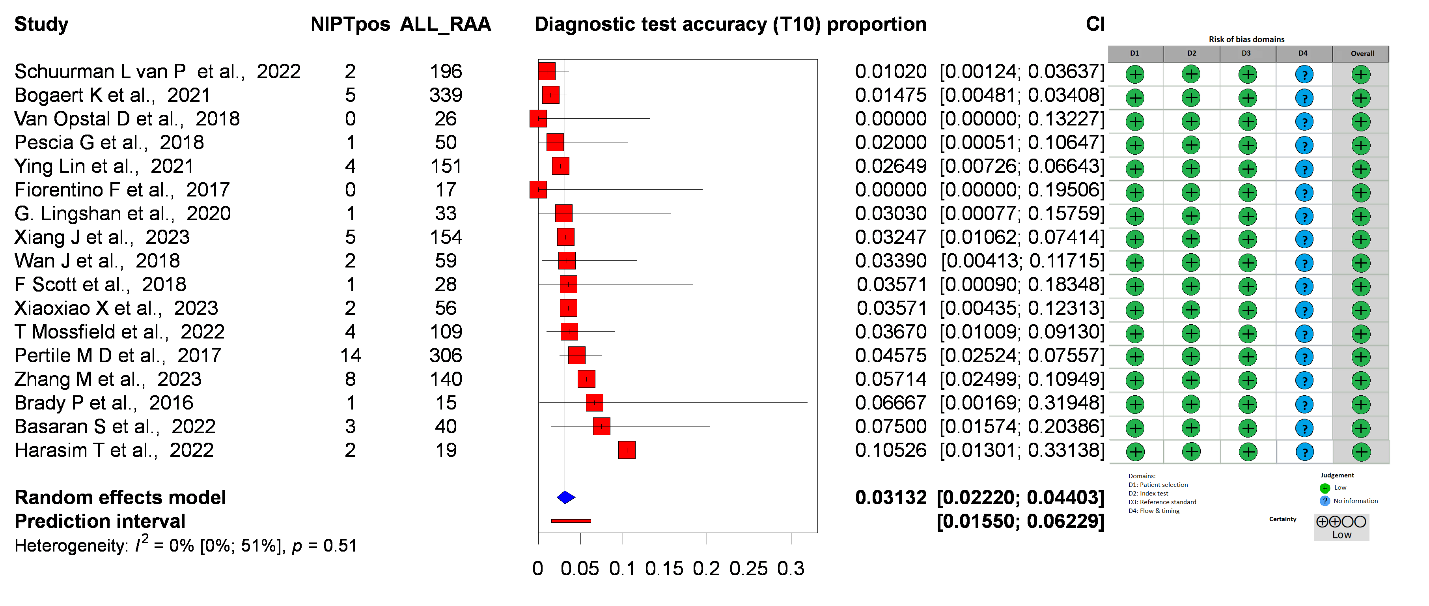


**Figure S30.** Forest plots representing the frequency of NIPT test for trisomy chromosome 11.


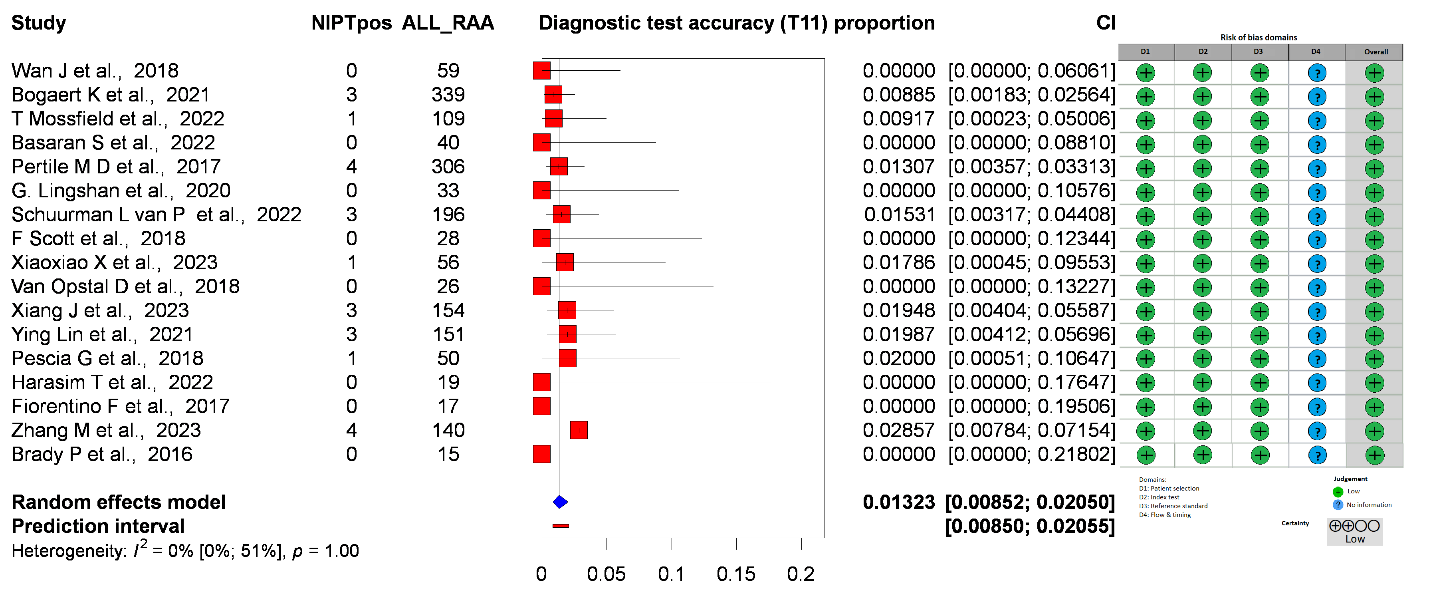


**Figure S31.** Forest plots representing the frequency of NIPT test for trisomy chromosome 12.


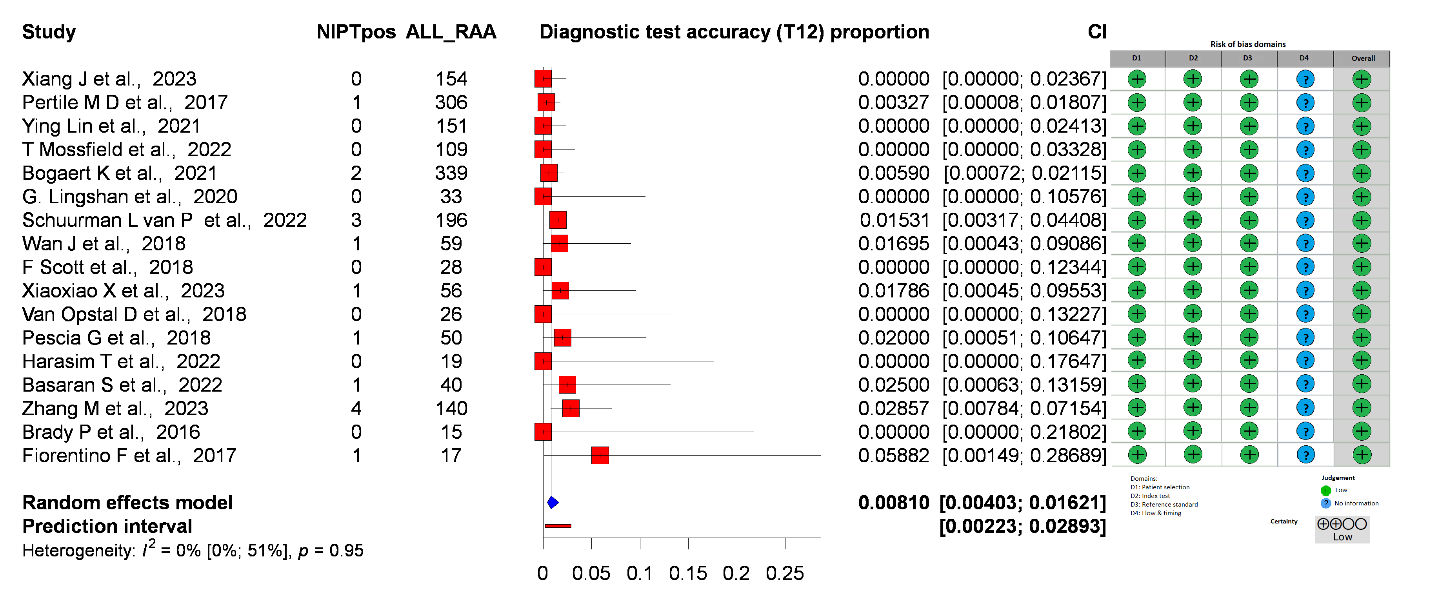


**Figure S32.** Forest plots representing the frequency of NIPT test for trisomy chromosome 14.


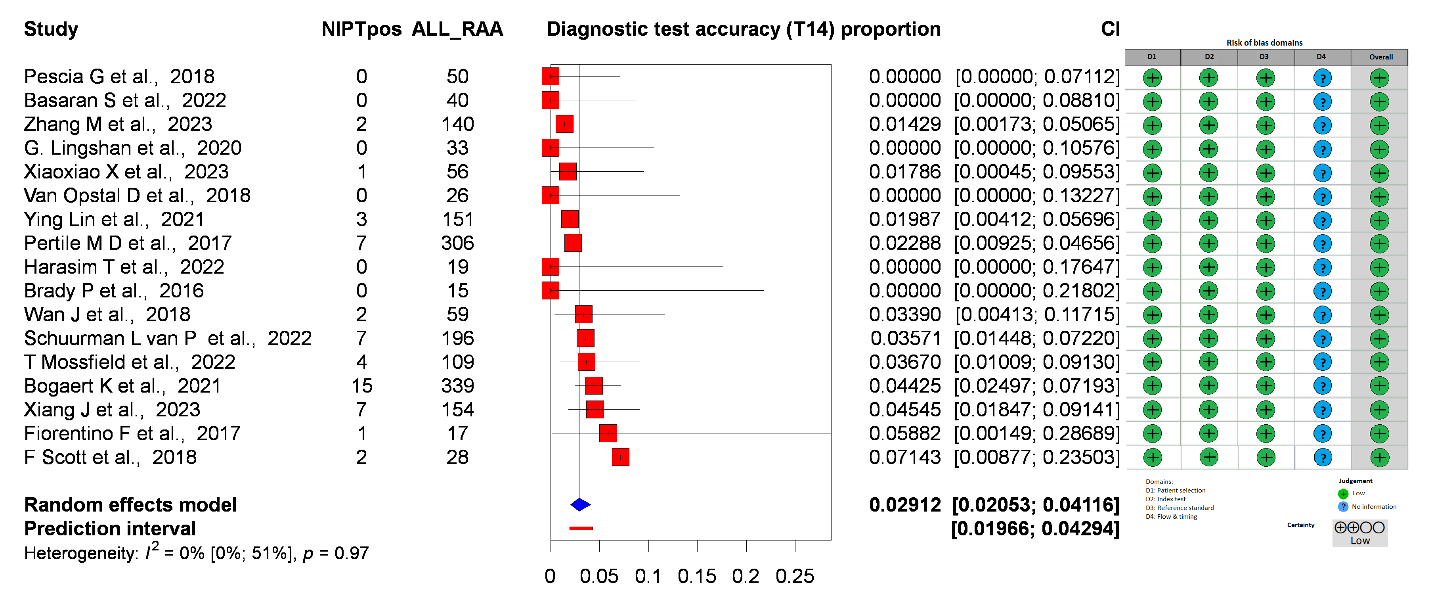


**Figure S33.** Forest plots representing the frequency of NIPT test for trisomy chromosome 15.


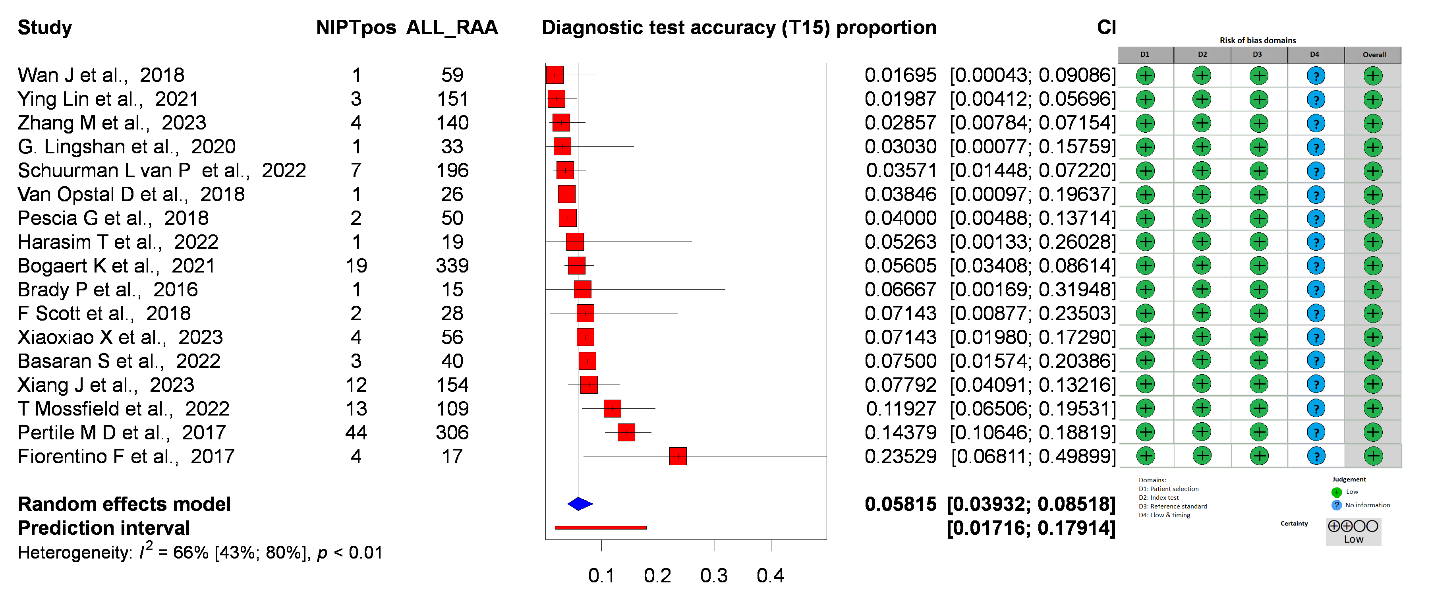


**Figure S34.** Forest plots representing the frequency of NIPT test for trisomy chromosome 16.


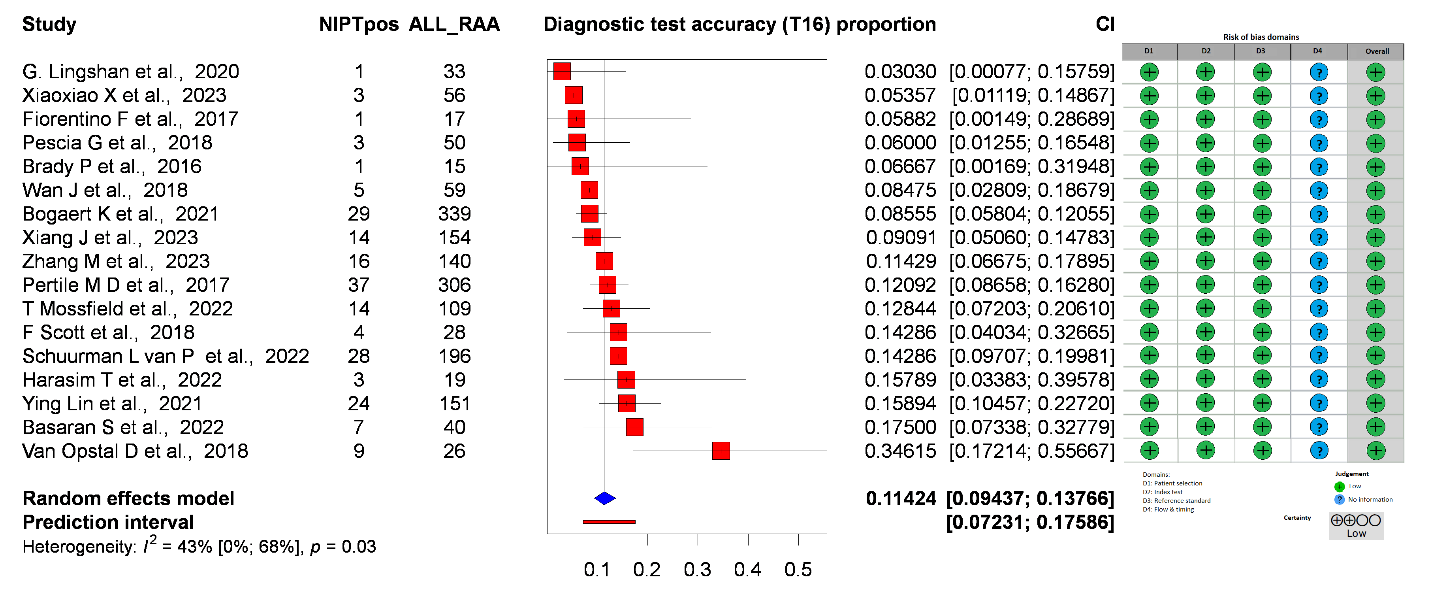


**Figure S35.** Forest plots representing the frequency of NIPT test for trisomy chromosome 17.


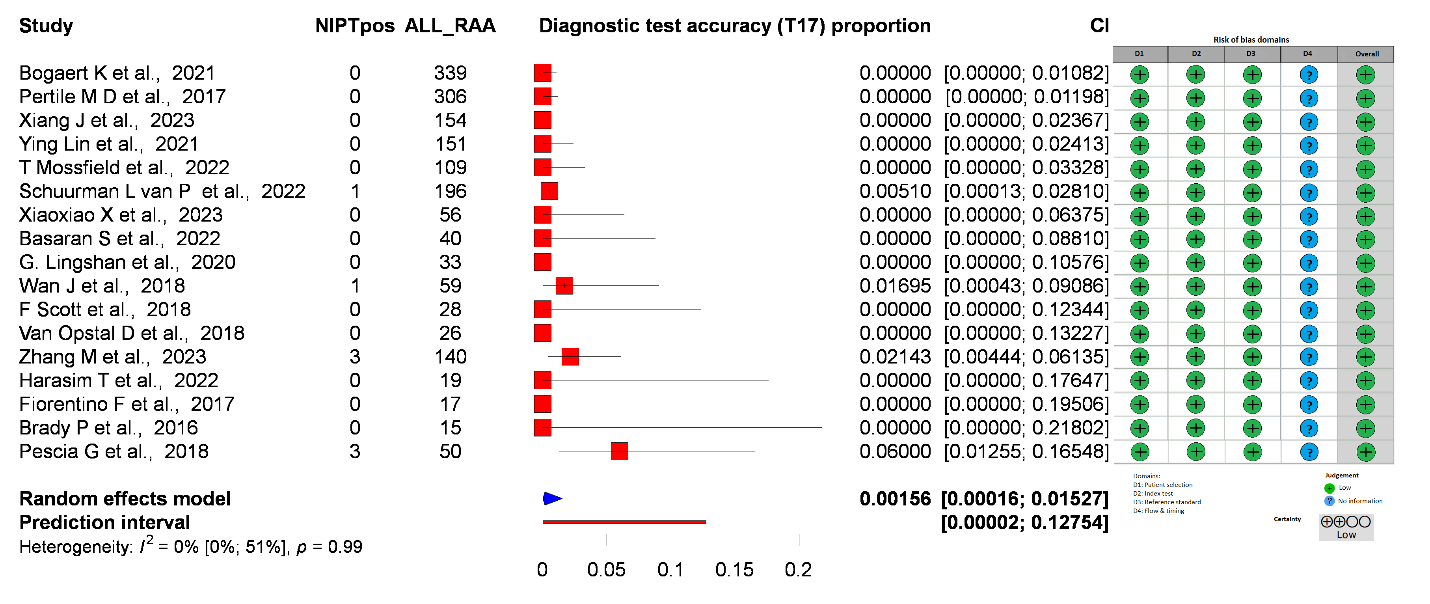


**Figure S36.** Forest plots representing the frequency of NIPT test for trisomy chromosome 19.


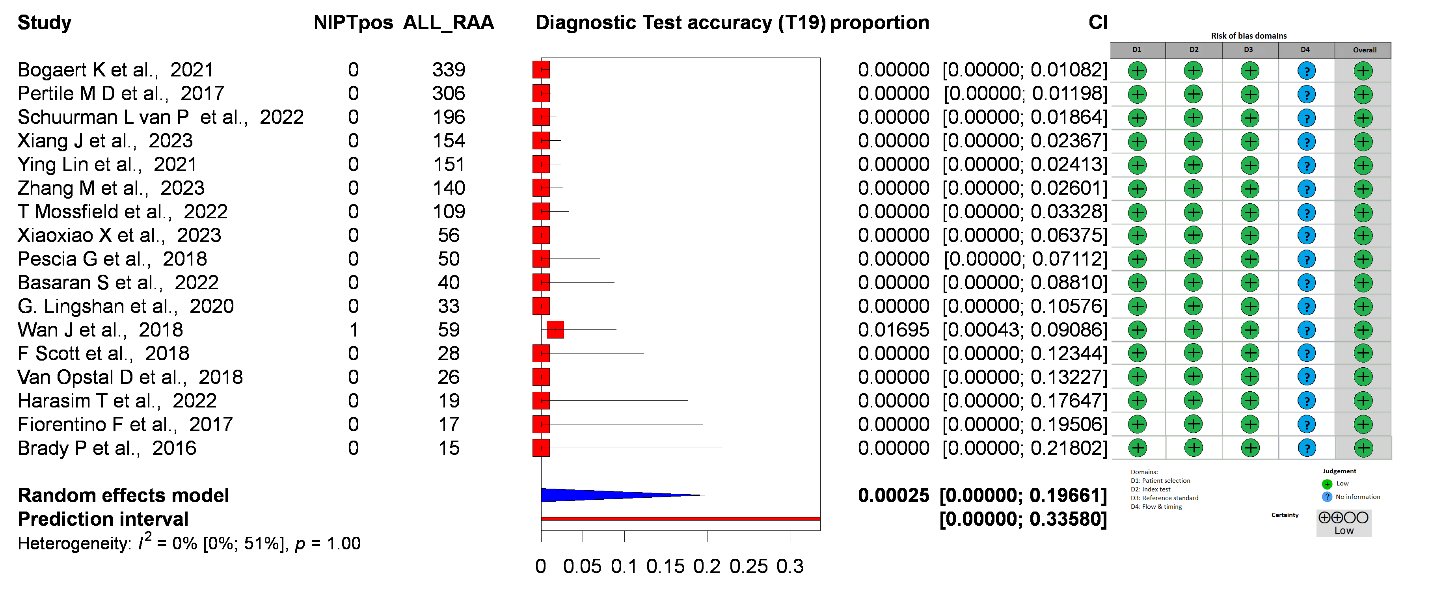


**Figure S37.** Forest plots representing the frequency of NIPT test for trisomy chromosome 20.


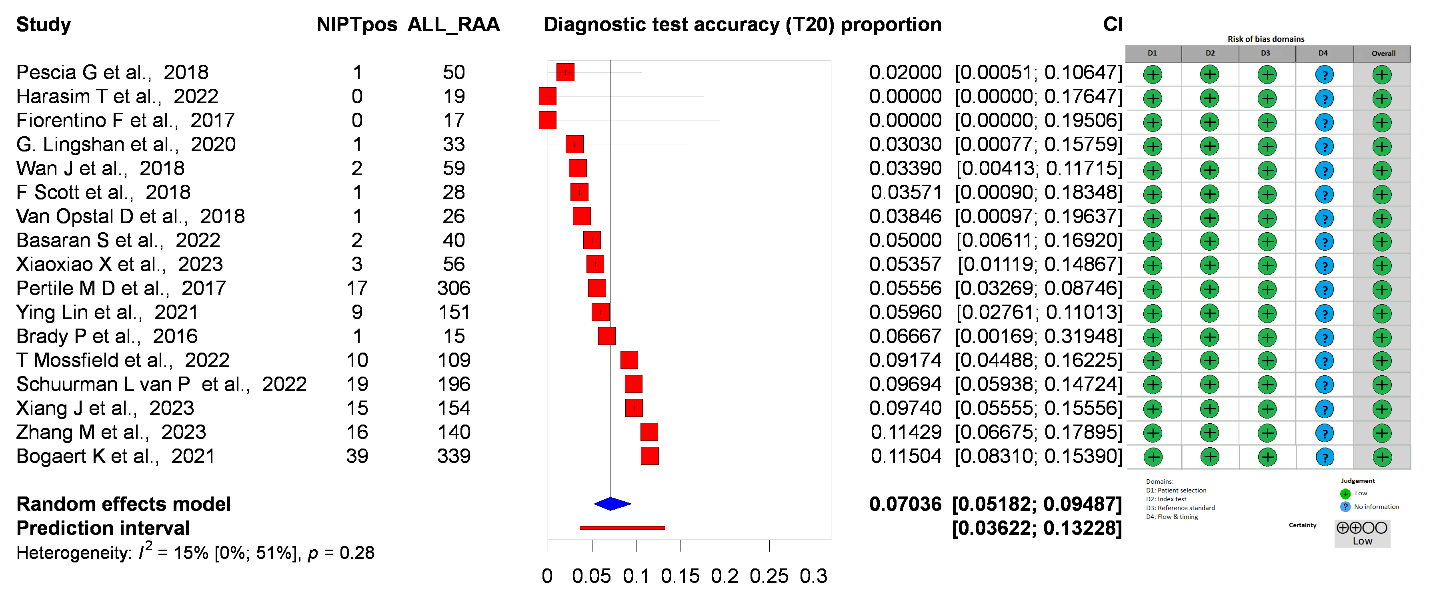


**Figure S38.** Forest plots representing the frequency of NIPT test for trisomy chromosome 22.


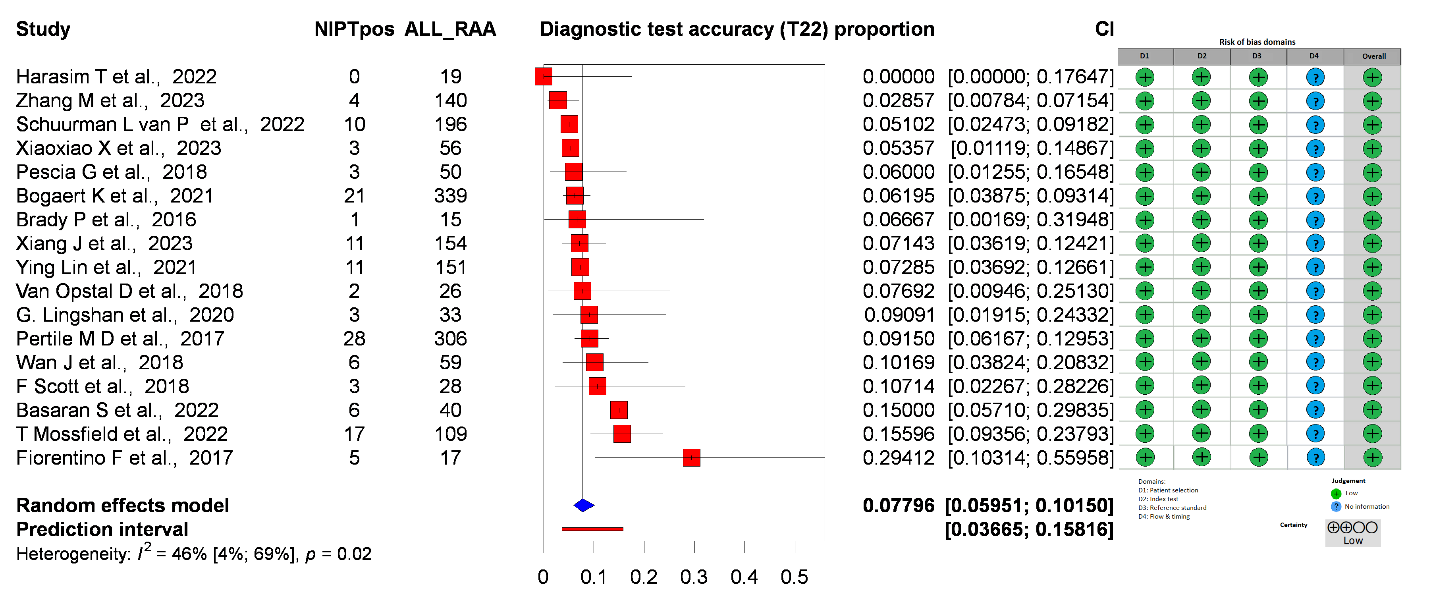


**Figure S39.** Forest plot for individual analysis for gestational age at sampling with confirmed methods


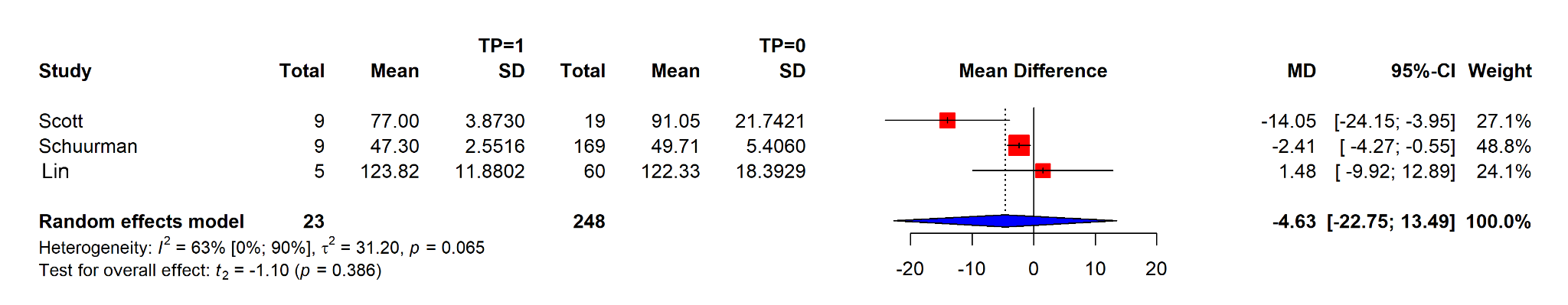


**Figure S40.** Forest plot for individual analysis for gestational age at sampling with extended methods


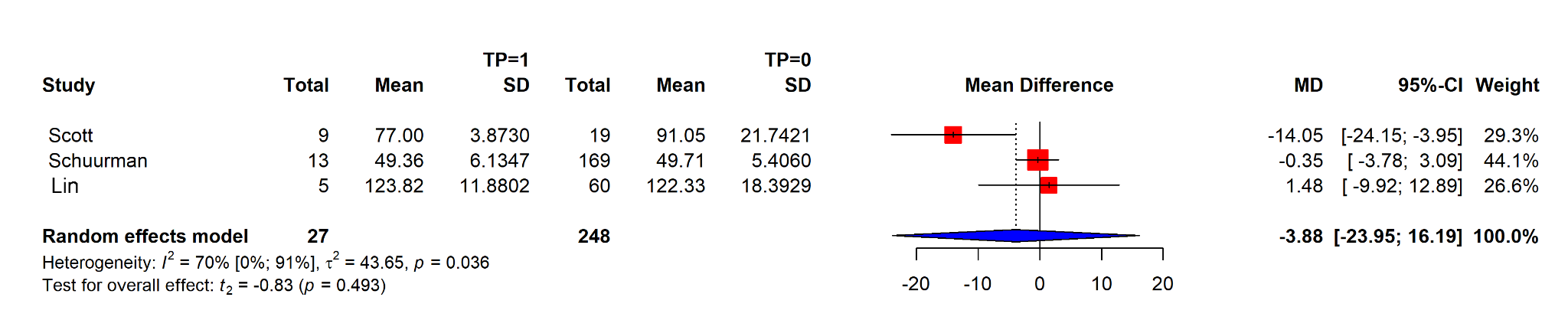


**Figure S41.** Forest plot for individual analysis for maternal age at sampling with confirmed methods


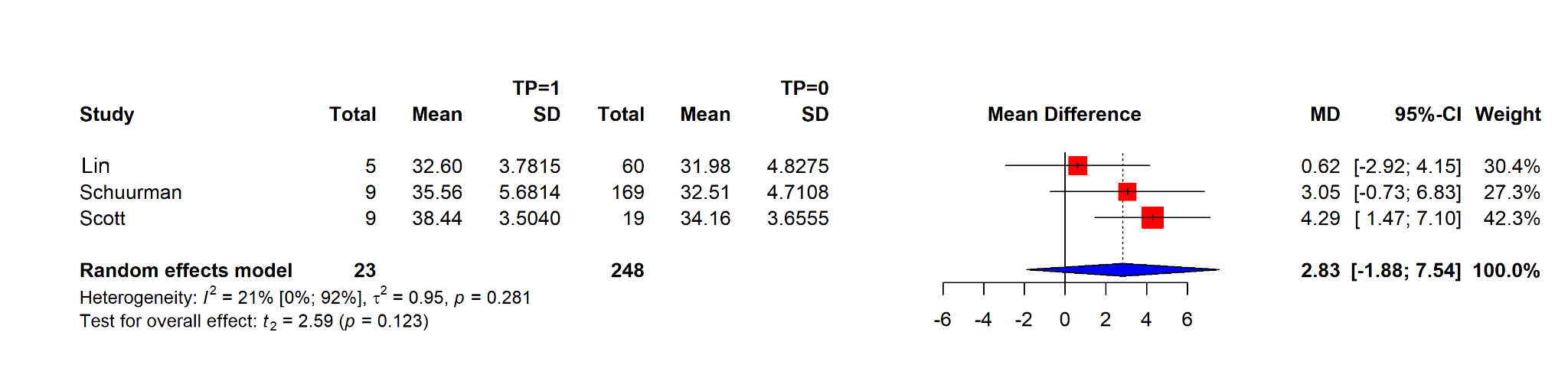


**Figure S42.** Forest plot for individual analysis for maternal age at sampling with extended methods


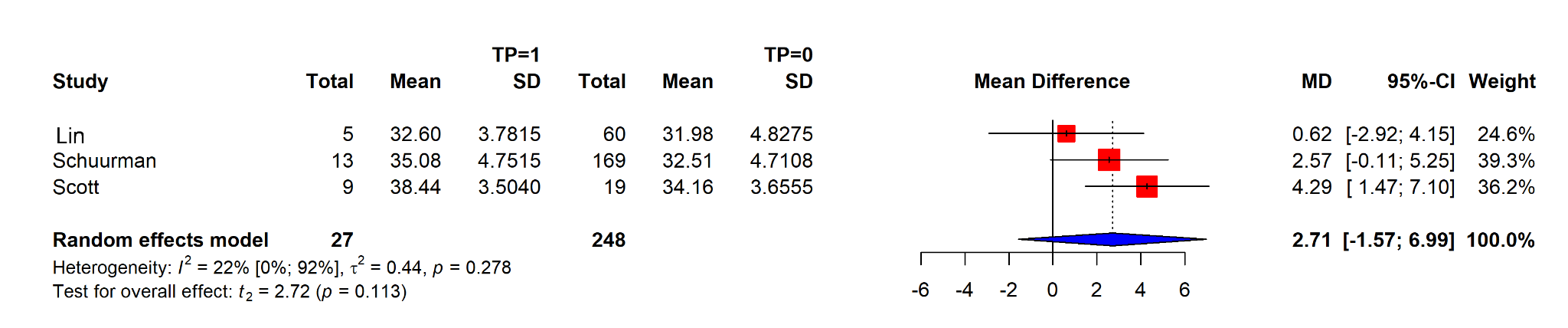


**Figure S43.** Linear regression test of funnel plot asymmetry for confirmed method


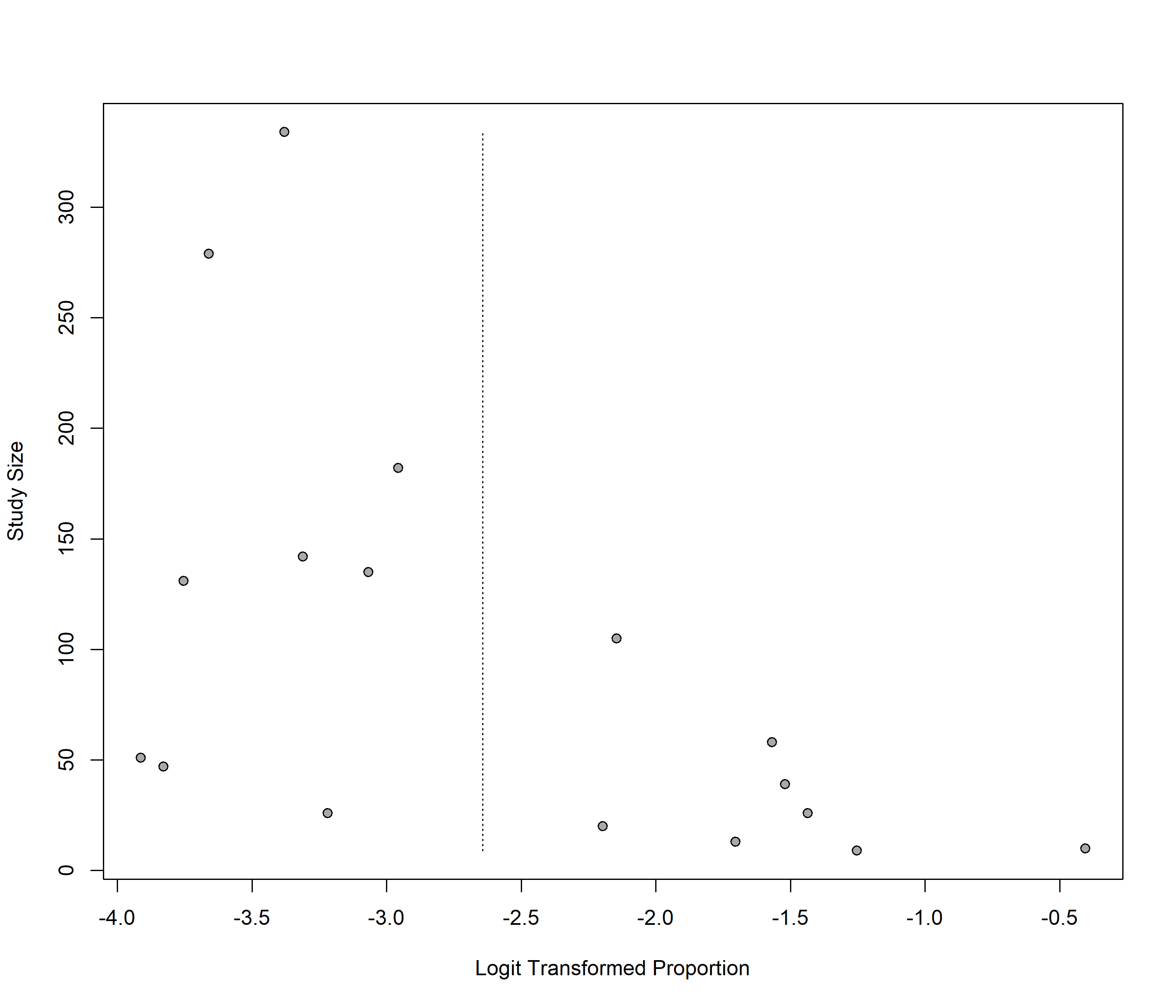


**Figure S44.** Linear regression test of funnel plot asymmetry for confirmed method without the largest outlier


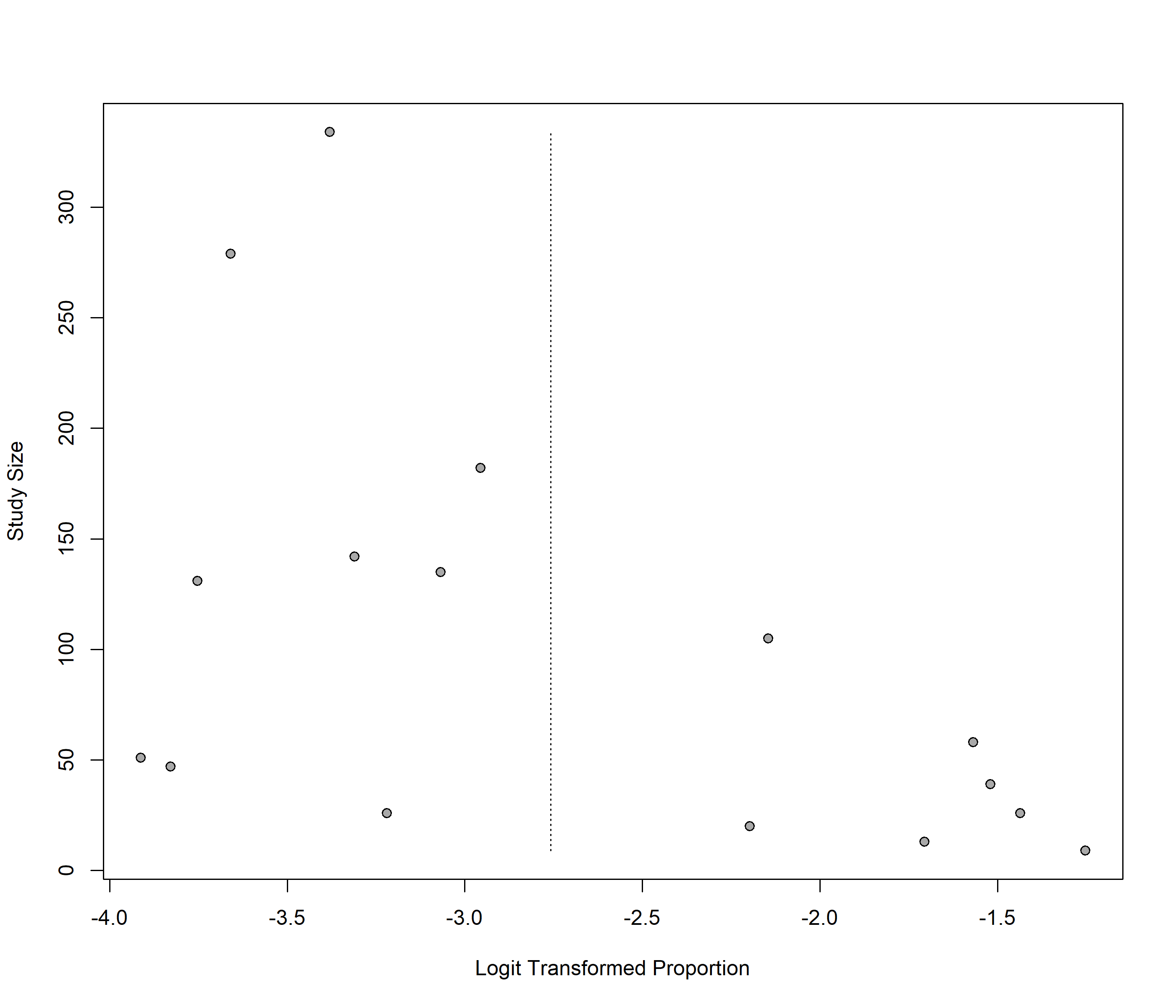


**Figure S45.** Linear regression test of funnel plot asymmetry for extended method


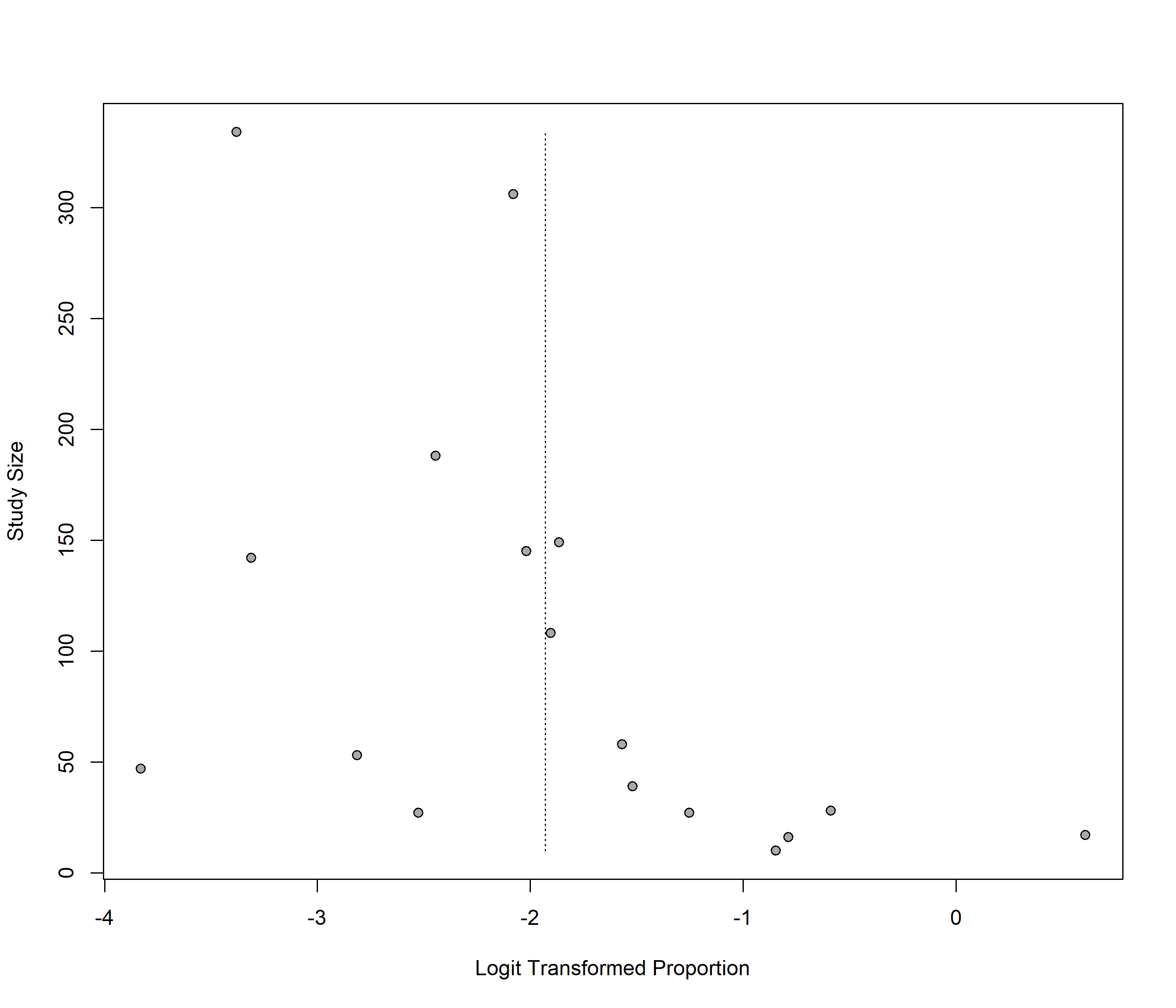


**Figure S46.** Linear regression test of funnel plot asymmetry for confirmed method without the largest outlier


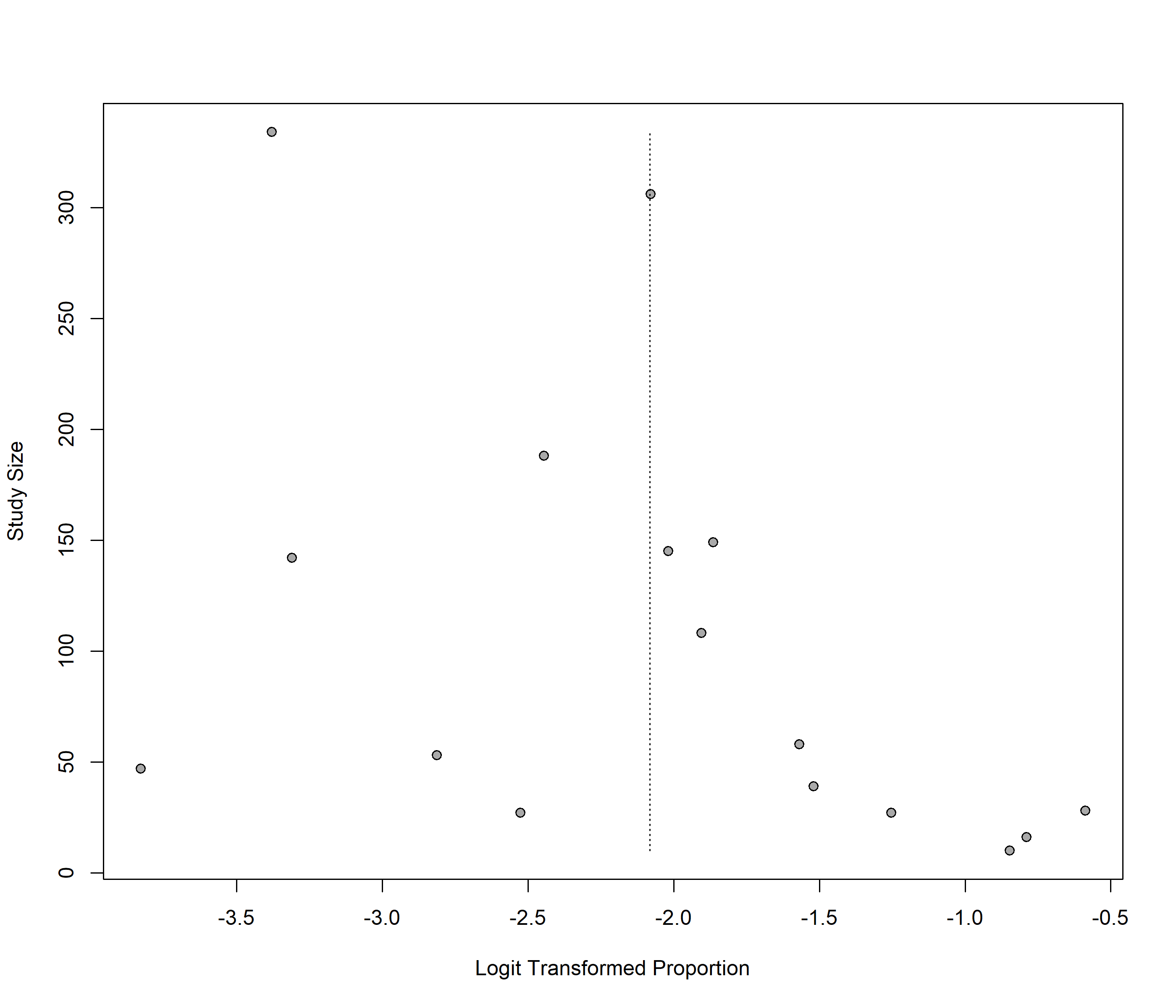


**Table S1.** Eligibility criteria of each included studies

| **Study (year)** | **Inclusion criteria** | **Exclusion criteria** |
| --- | --- | --- |
| G Lingshan, 2020 (1) | 18,016 pregnancies  undergoing NIPT at a single center in China from March 2017 to February 2020 | some abnormal  pregnancies might have been excluded by pre-test ultrasound before performing NIPT |
| T Mossfield, 2022(2) | study cohort included a  total of 109 cases from five different sites from Australia, Canada, Argentina, and South Africa | - |
| F Scott, 2018 (3) | patients was based on a singleton pregnancy, with no obvious abnormality, at a minimum of 10 weeks' gestation at sample collection | Two samples with multiple chromosomes with CCV levels above 1.02 were excluded |
| T Harasim , 2022(4) | minimal gestational age of 9 + 0 weeks, singleton or (vanishing) twin pregnancies | - |
| Schuurman L van P , 2022(5) | All women in the Dutch general obstetric population | 1. Ultrasound abnormalities in the fetus (including a nuchal translucency measurement ≥ 3.5 mm).  2. A non-vital pregnancy.  3. Carrier status (and/or her partner) of a chromosome aberration.  4. A malignancy at the time of the request.  5. A blood transfusion, stem cell- or organ transplantation or cancer immunotherapy in the past three months.  6. Age under 16 years old.  7. Unable to understand and consent to the purpose of the study, even with the help of an interpreter - in the counselors’ opinion.  8. Pregnancy not monitored by an obstetric care provider in the Netherlands. |
| K Bogaert , 2021 (6) | Pregnant women from 12 weeks | Higher-order pregnancies |
| J Wan , 2018 (7) | Guangzhou Women and Children's Medical Centre in China, from the beginning of the  service in February 2015 to January 2018. The test protocol required that the pregnancy had to be above 12 gestational weeks | major structural abnormalities |
| C Wang, 2021(8) | The inclusion criteria were as follows: maternal serological screening critical-risk value (1/1000 ≤ T21 < 1/270, 1/1000 ≤ T18 < 1/350), maternal serological screening high-risk value (T21 ≥ 1/270, T18 ≥ 1/350), contraindications  of prenatal diagnosis, missed serological screening or requested NIPT, advanced maternal age (35 years or older at the expected due date), abnormal single ultrasonic soft indexes such as unilateral or bilateral choroid plexus cyst, ventricular bright spot, slightly widened  lateral ventricle, critical thickening of the NT value (2.0 ≤ NT ≤ 3.0), IVF, or twin pregnancy. | Exclusion criteria for NIPT were as follows: not in gestational week 12 + 0 ~ 26 + 5, chromosome abnormality in one of the parents, ultrasonography showing structurally  abnormal foetuses, family history of genetic diseases or high-risk of genetic diseases, malignant tumour during pregnancy, allogeneic blood received within one year,  exogenous DNA introduced within 4 weeks, and transplantation and stem cell therapy, among others. |
| M D Pertile, 2017 (9) | Gestational age at time of sampling was greater than or equal to 10 weeks; a value for the NCDQ parameter was available; blood samples had been drawn into nonexpired Streck DNA Blood Collection Tubes (BCT) and had arrived at the laboratory within the time frame required for analysis and with sufficient volume for testing; and if multiple test samples at different gestational ages were received from the same pregnancy, only one blood sample was selected for study. The second (later) sample was selected, unless the data for that sample were incomplete. | gestational age of less than 10 weeks, (ii) inadequate blood volume, and (iii) blood collected into tubes other than Streck DNA BCT. In this cohort, clinical outcome information was only available for five subjects in whom a diagnosis of maternal malignancy was known. These women had previously consented to a WGS review of their NIPT data to participate in a different research study on discordant results due to maternal malignancy |
| G Pescia, 2018 (10) |  | transportation time >48 h, total DNA concentrations ≥4 ng/μl, and visible hemolysis (degree defined by photographic references). |
| Van Opstal D 2018 (11) | Pregnancy screeining between Apr 2014 and Apr 2015 | vanishing twins and are described in more detail in Oepkes et al [47] |
| Y Lin 2022 (12) | pregnancy with a high-risk  RATs report and complete clinical information | loss to follow-up |
| P Brady , 2016(13) |  |  |
| F Fiorentino, 2017 (14) | nonselected series of  pregnant women undergoing conventional cfDNA-based NIPT | low fraction of ffcDNA (<2%), assay failure |
| S Basaran, 2022 (15) | consecutive cases that underwent invasive testing following cfDNA testing |  |
| J Xiang, 2023 (16) | Pregnant women with an NIPT result indicative of RATs | Chromosome 19 was excluded from the analysis because of unreliable results caused by complex genomic characteristics. Trisomy 16 was also excluded. |
| M Zhang, 2023 (17) | The inclusion criteria were: gestational age between 12 + 0 and 26 + 5 weeks; singleton pregnancy; critical risk value of serological screening for pregnant women (1/1000 ≤ T21 < 1/270, 1/1000 ≤ T18 < 1/350) and high-risk value of maternal serological screening (T21 ≥ 1/270, T18 ≥ 1/350); and single fetal soft markers identified by ultrasound. | The exclusion criteria were: gestational age < 12 weeks; multiple pregnancies; fetal structural abnormalities on ultrasound; one or more parents with identified chromosomal abnormalities; pregnant patients who had received an allogeneic blood transfusion, stem cell therapy, transplant, or immunotherapy within the last year; pregnant patients with malignant tumors; and samples with severe hemolysis, blood coagulation, or low cell-free DNA content. |
| X Xiaoxiao, 2023 (18) | high-risk cases of rare chromosomal trisomies |  |

**Table S2.** Intervention and diagnostics in each group

| **Study (year)** | **Intervention** | **NIPT Platform** | **Confirmation methods** |
| --- | --- | --- | --- |
| G. Lingshan, 2020 (1) | GW-NIPT for RAAs | massively parallel sequencing | AC, karyotyping and CMA, follow up |
| T. Mossfield, 2022(2) | GW-NIPT for RAAs | massively parallel sequencing | CVS, AC, POC |
| F Scott, 2018 (3) | GW-NIPT for RAAs | massively parallel sequencing | aCGH from CVS, AC, postnatal blood |
| T Harasim , 2022(4) | GW-NIPT for RAAs and SCAs | massively parallel sequencing | CVS, AC, ultrasound examination, or other diagnostic interventions |
| Schuurman L van P , 2022(5) | GW-NIPT for RAAs and SCAs | massively parallel sequencing | genomic arrays, fluorescent in situ hybridization (FISH), and/or conventional karyotyping from CVS or AC |
| K Bogaert , 2021 (6) | GW-NIPT for RAAs | massively parallel sequencing | CNV analysis was performed on DNA extracted from chorionic villus  sampling (CVS) or amniotic fluid (AF) using the Agilent ISCA 60 K or 44 K  array (Agilent), Cytoscan 750 K array (Affymetrix), HumanCytoSNP-12 v2.1  BeadChip kit (Illumina), or by shallow genome sequencing (CNVSeq).  Fluorescence in situ hybridization (FISH) was performed following standard  procedures. Subsequently, when a discrepancy between the NIPS and the  invasive genetic test result was detected, women were requested to  donate the placenta upon delivery. |
| J Wann , 2018 (7) | GW-NIPT for RAAs | massively parallel sequencing | Karyotyping and microarray analysis were provided to subjects who  chose invasive prenatal testing. Chromosomal karyotyping was performed  according to the standard procedure. G‐banding karyotypes  of cultured amniotic fluid cells were performed at 320 to 400 band  level with a resolution of around 10 Mb and 25 metaphases counted.  Microarray analysis of the fetal genomic DNA extracted from fresh  amniotic fluid was performed by Cytoscan 750K (Affymetrix, Santa  Clara, CA) according to the manufacturer's instruction, and the  reporting threshold of the copy number result was set at 200 kb with  marker count ≥50. Data were visualized and analyzed with the Chromosome  Analysis Suite (ChAS) software (Affymetrix, Santa Clara, CA)  based on the GRCh37/hg19 assembly. |
| C Wang, 2021(8) | GW-NIPT for RAAs and SCAs | massively parallel sequencing | All high-risk pregnant women  were advised to undergo amniocentesis and confirmatory  prenatal diagnosis. We conducted a series of tests,  such as foetal ultrasound and clinical examination of  new-borns, for low-risk cases and patients with positive  results who refused a prenatal diagnosis.  Karyotyping and/or aCGH |
| M D Pertile, 2017 (9) | GW-NIPT for RAAs and SCAs | massively parallel sequencing | Flagged cases were evaluated by follow-up diagnostic testing using one or more of the following biologic samples: chorionic villi, amniocytes, POC, placental biopsies, or maternal peripheral blood. Analysis was performed using standard karyotyping, interphase fluorescence in situ hybridization, CMAs using SNPs, or a combination of these techniques. |
| G Pescia, 2018 (10) | GW-NIPT for RAAs and SCAs | massively parallel sequencing | Amniocentesis, array-CGH |
| Van Opstal D 2018 (11) | GW-NIPT for RAAs and SCAs | massively parallel sequencing | Chorionic villi were investigated with array using  DNA isolated from cytotrophoblast and mesenchymal core  separately.18 Uncultured amniotic fluid cells (AF) cells were  investigated with fluorescent in situ hybridization or array, and  cultured cells with conventional karyotyping, fluorescent in situ  hybridization, or array |
| Y Lin 2022 (12) | GW-NIPT for RAAs | massively parallel sequencing | amniocentesis and array-CGH, ultrasound, pregnancy follow up |
| P Brady , 2016(13) | GW-NIPT for RAAs and SCAs | massively parallel sequencing | Conventional chromosome analysis from amniocentesis |
| F Fiorentino, 2017 (14) | GW-NIPT for RAAs and SCAs | massively parallel sequencing | metaphase and/or array-CGH-based karyotyping after an invasive prenatal diagnostic procedure  or from products of conception, in the case of a spontaneous miscarriage, pregnancy outcome follow up. |
| S Basaran, 2022 (15) | GW-NIPT for RAAs and SCAs | massively parallel sequencing | All patients consented to an invasive procedure, i.e.,CVS, AC, or fetal blood sampling (FBS). |
| J Xiang, 2023 (16) | GW-NIPT for RAAs | massively parallel sequencing | Women with an NIPT result indicative of RATs were counseled to undergo confirmatory testing via amniocentesis. Maternal leukocyte and placenta biopsy were used to analyze the origin of falsepositive NIPT results. |
| M Zhang, 2023 (17) | GW-NIPT for RAAs and SCAs | massively parallel sequencing | The high-risk samples were analyzed using amniotic fluid karyotype and chromosome microarray analysis (CMA), and the pregnancy outcomes were followed up. |
| X Xiaoxiao, 2023 (18) | GW-NIPT for RAAs | massively parallel sequencing | Invasive prenatal diagnostic tests including chromosome karyotype analysis, chromosome microarray analysis, copy number variation sequencing, and fluorescence in situ hybridization were performed in all the cases after clinical counseling. |

**Table S3.** Risk of bias assessment using the Risk of Bias Quadas-2 tool


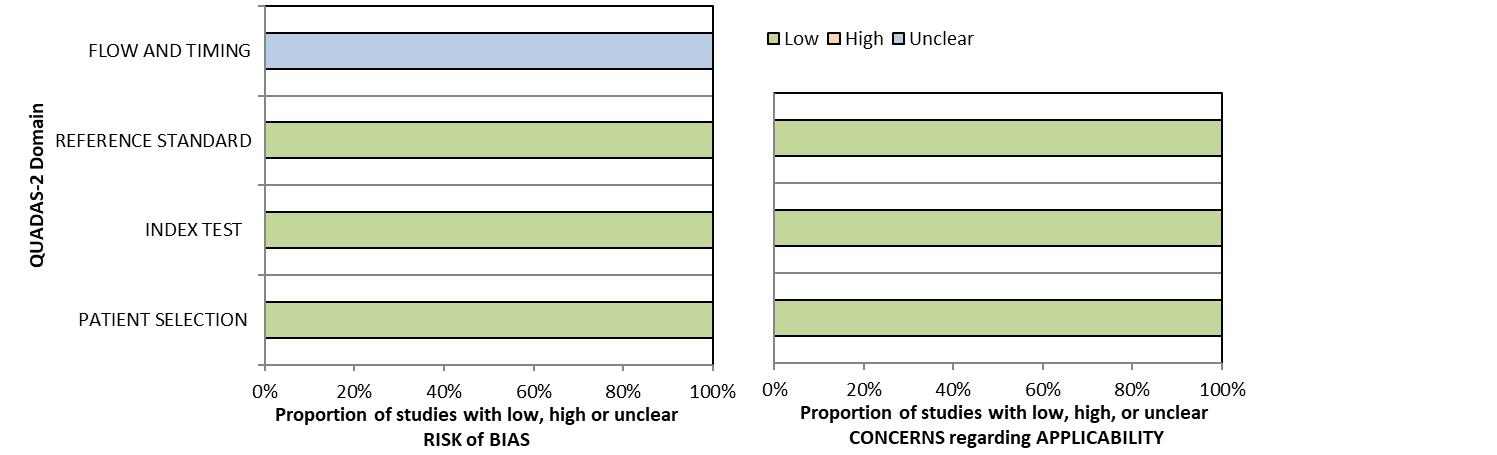

Supplement: S1 File — (DOCX) [file pone.0308008.s002.docx]
